# Synthesis of pyrazolopyrimidinones using a “one-pot” approach under microwave irradiation

**DOI:** 10.3762/bjoc.14.104

**Published:** 2018-05-28

**Authors:** Mark Kelada, John M D Walsh, Robert W Devine, Patrick McArdle, John C Stephens

**Affiliations:** 1 Department of Chemistry, Maynooth University, Maynooth, Co. Kildare, Ireland; 2 Department of Chemistry, National University of Ireland Galway, Co. Galway, Ireland

**Keywords:** microwave-assisted synthesis, nitrogen-fused heterocycle, one-pot, pyrimidinone

## Abstract

A simple one-pot method for the microwave-assisted synthesis of substituted pyrazolo[1,5-*a*]pyrimidinones, a core scaffold in many bioactive and pharmaceutically relevant compounds, has been established. A variety of substituents was tolerated at the 2 and 5 positions, including functionalized aryls, heterocycles, and alkyl groups.

## Introduction

**Figure 1:**
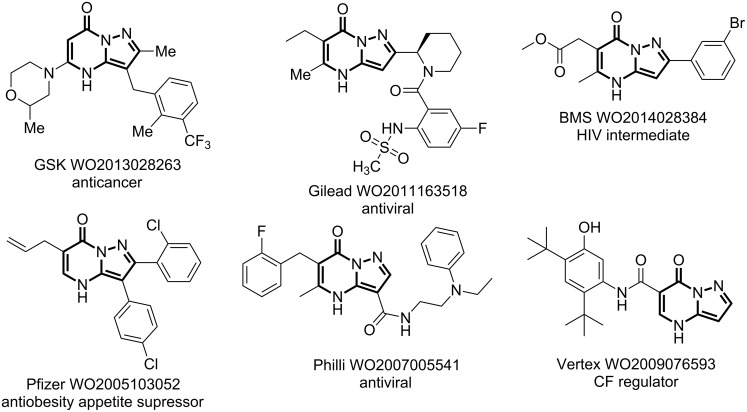
Bioactive pyrazolo[1,5-*a*]pyrimidinones.

The pyrazolo[1,5-*a*]pyrimidinone is a fused nitrogen-containing heterocyclic system and is of interest due to its role as a basic core scaffold in many bioactive and pharmaceutically relevant compounds, as well as its structural similarity to purine [1–4]. Pyrazolo[1,5-*a*]pyrimidinone derivatives have found use in the battle against several illnesses including cancer [5], viral infections [6–8], obesity [9], and cystic fibrosis [10] (Figure 1). It is these pharmacological properties, coupled with the pharmaceutical and fine chemical industries interest in synthetic processes that utilize cleaner and more efficient technology, which has stimulated the search for improved synthetic procedures for their generation.

Consequently, strategies for the synthesis of compounds of value, such as the pyrazolo[1,5-*a*]pyrimidinones, that employ one-pot syntheses and/or processes have been encouraged and have become a major focus for many synthetic chemists [11]. As such, a convenient and general method of preparing the scaffold would be of significant interest to the pharmaceutical industry. Herein, we report such a strategy for the simple synthesis of functionalized pyrazolo[1,5-*a*]pyrimidinones that employs a one-pot microwave-assisted approach.

## Findings

A number of synthetic approaches have been developed for the synthesis of privileged nitrogen-fused bicyclic systems such as the pyrazolo[3,4-*b*]pyridines, pyrazolo[1,5-*a*]pyrimidines and pyrazolo[1,5-*a*]pyrimidinones [12–18]. The majority of methods used in the generation of the pyrazolo[1,5-*a*]pyrimidinones employ a two-step process, which first requires the synthesis and isolation of the intermediate 5-aminopyrazoles followed by their reaction with an appropriate β-dicarbonyl compound [1–4,19–29]. A very recent and excellent review by Aggarwal et al. covers the use and versatility of 5-aminopyrazoles in the synthesis of a range of pyrazoloazines [30].

As part of a study into the development of novel biologically active compounds based on the pyrazolopyrimidinone scaffold, we sought to develop a simple one-pot synthesis of the nitrogen-fused bicyclic system. In order to establish reaction conditions for this one-pot synthesis, we began by seeking general reaction conditions for the microwave-assisted generation of the intermediate 5-aminopyrazoles. Once established, this methodology could be expanded to generate a one-pot synthesis of pyrazolo[1,5-*a*]pyrimidinones.

Typical methods for preparing 5-aminopyrazoles require conventional heating of the appropriate β-ketonitrile in the presence of a hydrazine [1–4,19–29], with Rao et al. [31] and Bagley et al. [32] reporting microwave-assisted protocols, the former requiring an acid catalyst. In order to conduct a solvent screen we chose β-ketonitrile **1a** as a model substrate and reacted it with hydrazine, employing a constant temperature of 150 °C and a reaction time of 5 min. Results from the solvent screen can be found in Table 1, with high isolated yields of the product 5-aminopyrazole **2a** in all cases. As expected, a significant improvement in reaction time was observed under microwave conditions in comparison with conventional heating (Table 1, entries 7, 8 and 9). Methanol was subsequently selected as the solvent of choice, due to the higher yield generated and its lower cost when compared with acetonitrile. Temperature and time were then varied with the best combination of temperature and time found to be 150 °C for 5 min (Table 2, entry 5).

**Table 1:** Solvent screen for the synthesis of 5-aminopyrazole **2a**.^a^

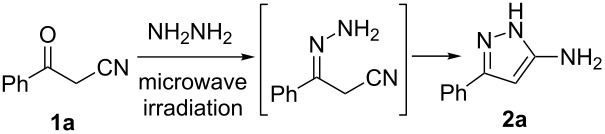			

Entry	Heating method	Solvent	Yield^b^ %

1	MW	DCM	82
2	MW	toluene	83
3	MW	Pet ether	88
4	MW	EtOAc	92
5	MW	MeCN	96
6	MW	petroleum ether/MeOH 9:1	99
7	MW	MeOH	99
8^c^	convent.	MeOH	60
9^d^	convent.	MeOH	30

^a^Reaction conditions: ketonitrile (0.9 mmol, 1.0 equiv), hydrazine (1.2 mmol, 1.3 equiv), solvent (1 mL) were heated under microwave conditions (100 W, 150 °C) for 5 min. ^b^Isolated yield. ^c^Reaction conditions: ketonitrile (0.9 mmol, 1.0 equiv), hydrazine (1.2 mmol, 1.3 equiv), solvent (1 mL) were heated under conventional heating conditions at reflux for 17 h. ^d^Reaction conditions: ketonitrile (0.9 mmol, 1.0 equiv), hydrazine (1.2 mmol, 1.3 equiv), solvent (1 mL) were heated under conventional heating conditions at reflux for 5 min.

**Table 2:** Temperature and time variations for the synthesis of 5-aminopyrazole **2a**.^a^

Entry	*T* (°C)	Time (min)	Yield^b^ %

1	120	40	84
2	130	30	96
3	130	5	53
4	140	20	98
5	150	5	99

^a^Reaction conditions: ketonitrile (0.9 mmol, 1.0 equiv), hydrazine (1.2 mmol, 1.3 equiv), MeOH (1 mL) were heated under microwave conditions (100 W). ^b^Isolated yield.

**Scheme 1:**
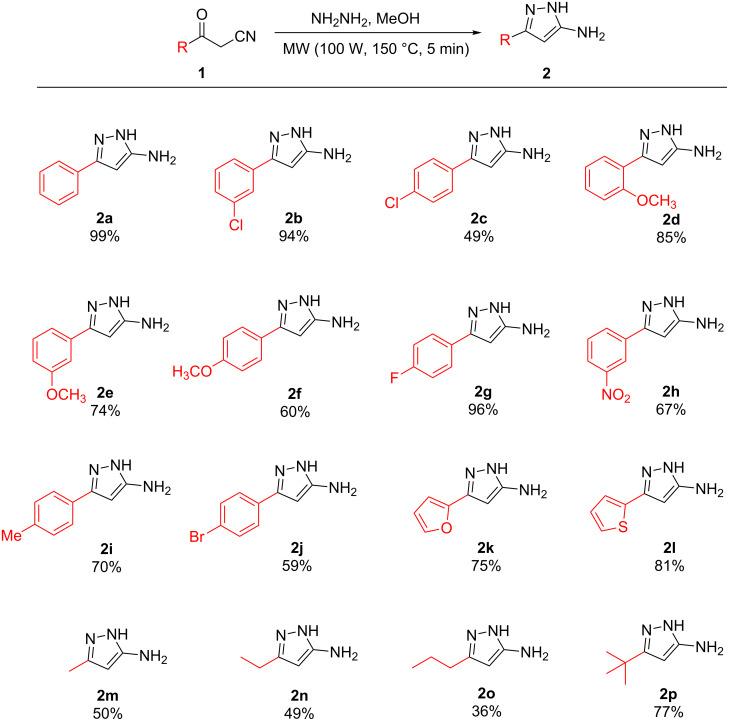
Synthesis of 5-aminopyrazoles. Reaction conditions: ketonitrile (2.0 mmol, 1.0 equiv), hydrazine (2.6 mmol, 1.3 equiv), MeOH (1 mL) were heated under microwave conditions (100 W, 150 °C) for 5 min. Isolated yields.

A substrate scope study showed that a variety of substituents is tolerated. Aromatic groups with electron-withdrawing and electron-donating substituents at the *ortho*, *meta* and *para* positions were successfully explored (Scheme 1). The heterocyclic furan and thiophene substituents allowed generation of the desired 5-aminopyrazoles in 75% and 81% yields, respectively (Scheme 1). Alkyl groups appeared to be less well tolerated, where the corresponding alkyl-substituted 5-aminopyrazoles were isolated in lower yields.

With a convenient and general microwave-assisted method for the synthesis of the 5-aminopyrazoles in hand, we next focused our attention on its application to the one-pot synthesis of pyrazolo[1,5-*a*]pyrimidinones.

**Scheme 2:**
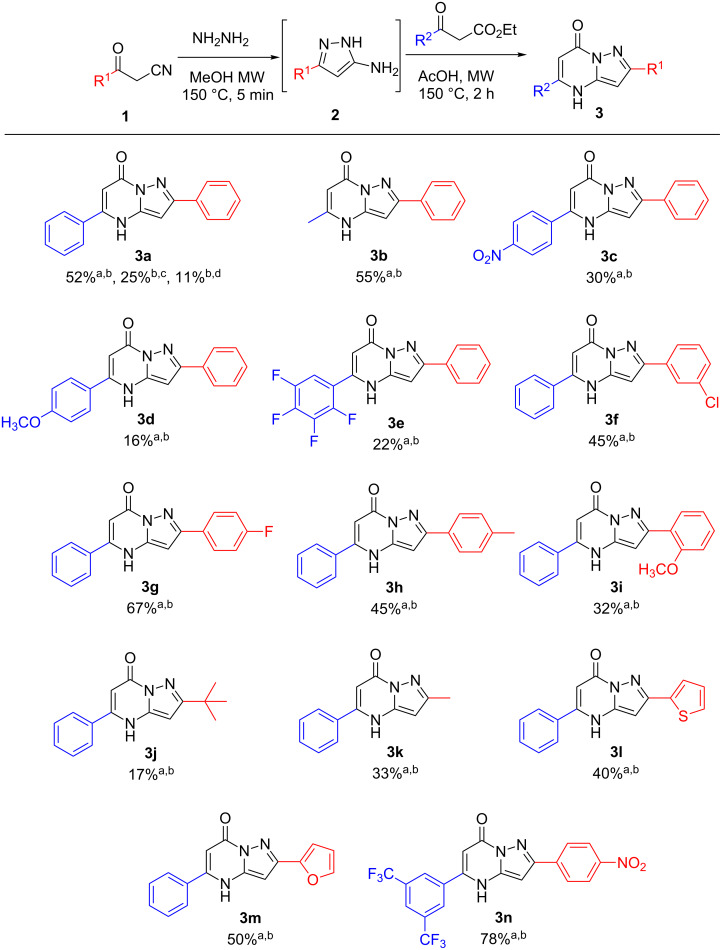
One-pot synthesis of pyrazolo[1,5-*a*]pyrimidinones. ^a^Reaction conditions: ketonitrile (0.9 mmol, 1.0 equiv), hydrazine (1.2 mmol, 1.3 equiv), MeOH (1 mL) were heated under microwave conditions (100 W, 150 °C) for 5 min followed by the addition of AcOH (0.5 mmol, 0.6 equiv) and ketoester (0.9 mmol, 1.0 equiv) and heated under microwave conditions (100 W, 150 °C) for a further 2 h. ^b^Isolated yield. ^c^Reactions conditions: isolated aminopyrazole (0.9 mmol, 1.0 equiv), AcOH (0.5 mmol, 0.6 equiv), ketoester (0.9 mmol, 1.0 equiv) in methanol (2 mL) were heated using conventional heating conditions at reflux for 18 h. ^d^Reactions conditions: isolated aminopyrazole (0.9 mmol, 1.0 equiv), AcOH (0.5 mmol, 0.6 equiv), ketoester (0.9 mmol, 1.0 equiv) in methanol (2 mL) were heated using conventional heating conditions at reflux for 2 h.

The synthesis of pyrazolo[1,5-*a*]pyrimidinone **3a** was chosen as the model reaction (Scheme 2). Initial reaction conditions were chosen to match the already developed microwave-assisted synthesis of the 5-aminopyrazoles. A solution of the β-ketonitrile in methanol was treated with hydrazine and heated to 150 °C under microwave irradiation for 5 min. The β-ketoester and acetic acid were then simply added to the pot and the reaction heated at the same temperature, 150 °C, under microwave irradiation for a further 2 h. The target pyrazolo[1,5-*a*]pyrimidinone was subsequently isolated in an overall yield of 52%. Once more, a significant improvement was observed using microwave conditions in comparison with conventional heating.

When a mixture of previously isolated 5-aminopyrazole **2a**, β-ketoester, and acetic acid were heated under conventional refluxing conditions for 18 h, the product pyrazolo[1,5-*a*]pyrimidinone **3a** could only be isolated in a 25% yield. As expected, heating the reaction mixture for 2 h at reflux gave a lower isolated yield of 11%. A one-pot procedure under conventional refluxing conditions was also carried out in direct comparison with the microwave method, i.e., a solution of the β-ketonitrile in methanol was treated with hydrazine and refluxed for 5 min. The β-ketoester and acetic acid were then added to the pot and the reaction refluxed for a further 2 h. A complex mixture resulted consisting mostly of starting β-ketonitrile.

The superior performance of the microwave reaction, in terms of yield and reaction time, could result from the higher temperature and pressure achieved. The substrate scope for the one-pot reaction was then explored, with variations of both R^1^ and R^2^ groups at the pyrazolo[1,5-*a*]pyrimidinone core (Scheme 2).

Pyrazolo[1,5-*a*]pyrimidinones containing aromatic groups with electron-withdrawing and electron-donating substituents at the *ortho*, *meta* and *para* positions were generated, including the multi-substituted **3n**, as were those with heterocyclic furyl and thienyl substituents (Scheme 2). Alkyl-substituted pyrazolo[1,5-*a*]pyrimidinones were also synthesized using this one-pot method (Scheme 2).

**Figure 2:**
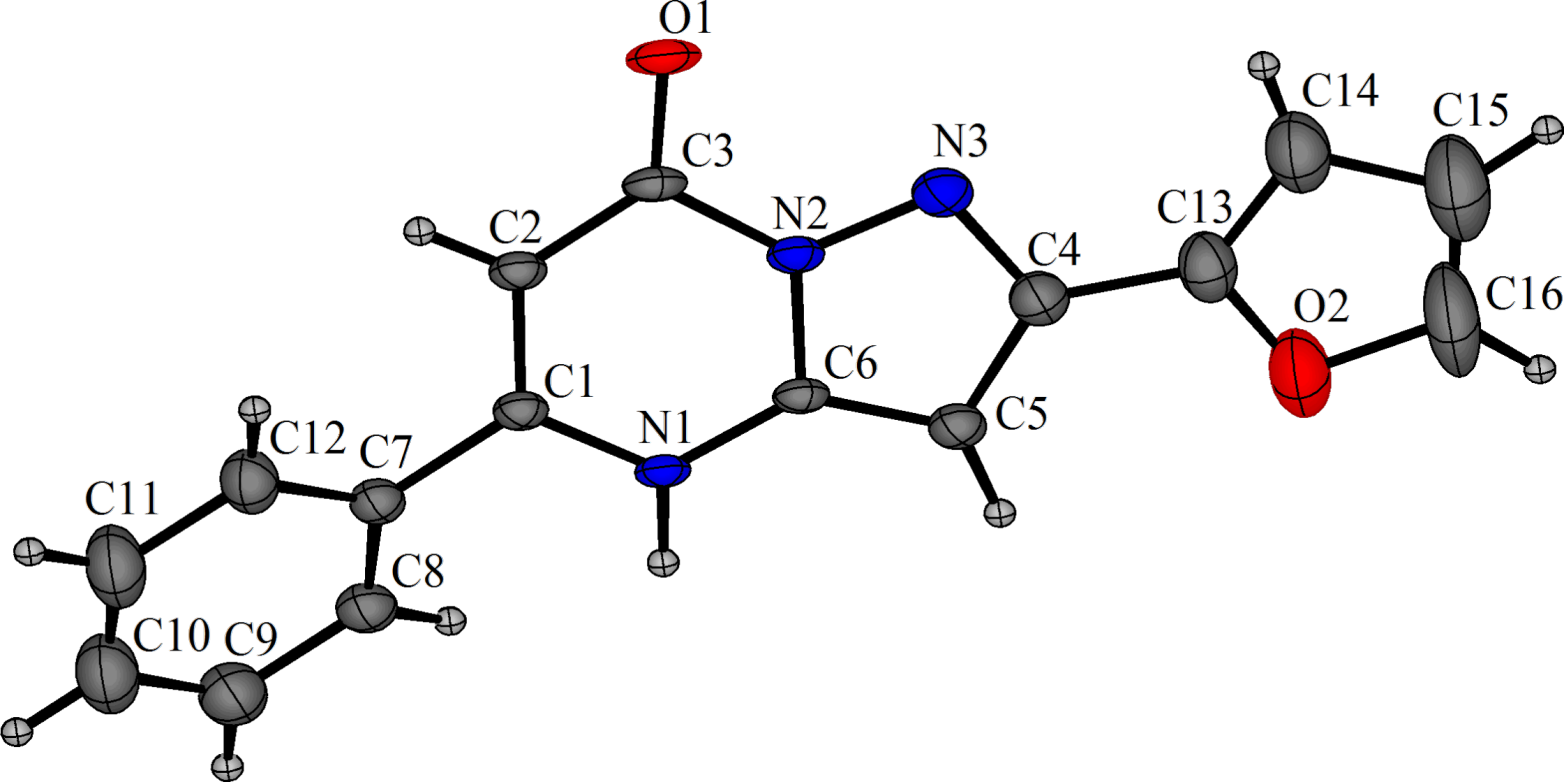
X-ray crystal structure of pyrazolo[1,5-*a*]pyrimidinone **3m** with ellipsoids at 50% probability.

The structures of the pyrazolo[1,5-*a*]pyrimidinones were characterized using ^1^H and ^13^C NMR spectral data, HRMS, and IR spectroscopy. In addition, an X-ray crystal structure was obtained for pyrazolo[1,5-*a*]pyrimidinone **3m** and is shown in Figure 2.

## Conclusion

In conclusion, we have developed a new facile method for the one-pot microwave-assisted synthesis of substituted pyrazolo[1,5-*a*]pyrimidinones, a core scaffold in many bioactive and pharmaceutically relevant compounds. A variety of functional groups was tolerated at the 2 and 5 positions, including functionalized aryls, heterocycles, and alkyl groups. Furthermore, an efficient and general microwave-assisted synthesis of versatile 5-aminopyrazoles is reported.

## Supporting Information

CCDC-1588686 contains the crystallographic data for **3m**. The data can be obtained from The Cambridge Crystallographic Data Centre (CCDC) via http://www.ccdc.cam.ac.uk/data_request/cif.

File 1 Experimental section, NMR spectra of all synthesized compounds and crystallographic data of compound **3m**.
