# Supplementary material for: Synthesis of pyrazolopyrimidinones using a “one-pot” approach under microwave irradiation
Source: Beilstein J Org Chem. 2018 May 28;14:1222–8. doi: 10.3762/bjoc.14.104 (PMC6009099; doi:10.3762/bjoc.14.104)
Supplement: File 1 — Experimental section, NMR spectra of all synthesized compounds and crystallographic data of compound 3m. [file Beilstein_J_Org_Chem-14-1222-s001.pdf]

Supporting Information

for

Synthesis of pyrazolopyrimidinones using a “one-pot” approach under microwave irradiation

Mark Kelada[1], John M. D. Walsh[1], Robert W. Devine[1], Patrick McArdle[2] and John C. Stephens[1,*]

Address: [1]Department of Chemistry, Maynooth University, Maynooth, Co. Kildare, Ireland and [2]Department of Chemistry, National University of Ireland Galway, Co. Galway, Ireland

Email: John C. Stephens* - john.stephens@mu.ie

* Corresponding author

Experimental section, NMR spectra of all synthesized compounds and crystallographic data of compound 3m

Table of Contents

1. Experimental **S4**

1.1. General information **S4**

1.2. General procedure of microwave synthesis of pyrazoles **S4**

1.2.1. 3-Phenyl-1*H*-pyrazol-5-amine **2a** S5

1.2.2. 3-(3-Chlorophenyl)-1*H*-pyrazol-5-amine **2b** S8

1.2.3. 3-(4-Chlorophenyl)-1*H*-pyrazol-5-amine **2c** S10

1.2.4. 3-(2-Methoxyphenyl)-1*H*-pyrazol-5-amine **2d** S11

1.2.5. 3-(3-Methoxyphenyl)-1*H*-pyrazol-5-amine **2e** S13

1.2.6. 3-(4-Methoxyphenyl)-1*H*-pyrazol-5-amine **2f** S15

1.2.7. 3-(4-Fluorophenyl)-1*H*-pyrazol-5-amine **2g** S16

1.2.8. 3-(3-Nitrophenyl)-1*H*-pyrazol-5-amine **2h** S18

1.2.9. 3-(*p*-Tolyl)-1*H*-pyrazol-5-amine **2i** S20

1.2.10. 3-(4-Bromophenyl)-1*H*-pyrazol-5-amine **2j** S22

1.2.11. 3-(Furan-2-yl)-1*H*-pyrazol-5-amine **2k** S23

1.2.12. 3-(Thiophenyl-2-yl)-1*H*-pyrazol-5-amine **2l** S25

1.2.13. 3-Methyl-1*H*-pyrazol-5-amine **2m** S27

1.2.14. 3-Ethyl-1*H*-pyrazol-5-amine **2n** S29

1.2.15. 3-Propyl-1*H*-pyrazol-5-amine **2o** S31

1.2.16. 3-(*tert*-Butyl)-1*H*-pyrazol-5-amine **2p** S33

1.3. General procedure of one pot synthesis of pyrazolopyrimidones **S35**

1.3.1. 2,5-Diphenylpyrazolo[1,5-*a*]pyrimidin-7(4*H*)-one **3a** S36

1.3.2. 5-Methyl-2-phenylpyrazolo[1,5-*a*]pyrimidin-7(4*H*)-one **3b** S39

1.3.3. 5-(4-Nitrophenyl)-2-phenylpyrazolo[1,5-*a*]pyrimidin-7(4*H*)-one **3c** S41

1.3.4. 5-(4-Methoxyphenyl)-2-phenylpyrazolo[1,5-*a*]pyrimidin-7(4*H*)-one **3d** S43

1.3.5. 2-Phenyl-5-(2,3,4,5-tetrafluorophenyl)pyrazolo[1,5-*a*]pyrimidin-7(4*H*)-one **3e** S45

1.3.6. 2-(3-Chlorophenyl)-5-phenylpyrazolo[1,5-*a*]pyrimidin-7(4*H*)-one **3f** S47

1.3.7. 2-(4-Fluorophenyl)-5-phenylpyrazolo[1,5-*a*]pyrimidin-7(4*H*)-one **3g** S49

1.3.8. 5-Phenyl-2-(*p*-tolyl)pyrazolo[1,5-*a*]pyrimidin-7(4*H*)-one **3h** S51

1.3.9. 2-(2-Methoxyphenyl)-5-phenylpyrazolo[1,5-*a*]pyrimidin-7(4*H*)-one **3i** S53

1.3.10. 2-(*tert*-Butyl)-5-phenylpyrazolo[1,5-*a*]pyrimidin-7(4*H*)-one **3j** S55

1.3.11. 2-Methyl-5-phenylpyrazolo[1,5-*a*]pyrimidin-7(4*H*)-one **3k** S57

1.3.12. 5-Phenyl-2-(thiophen-2-yl)pyrazolo[1,5-*a*]pyrimidin-7(4*H*)-one **3l** S59

1.3.13. 2-(Furan-2-yl)-5-phenylpyrazolo[1,5-*a*]pyrimidin-7(4*H*)-one **3m** S61

1.3.14. 5-(3,5-Bis(trifluoromethyl)phenyl)-2-(4-nitrophenyl)pyrazolo[1,5-*a*]pyrimidin-7(4*H*)-one **3n** S61

2. X-ray crystallographic data S65

3. Microwave profiles for compound 3m S73

4. References S75

1. Experimental

1.1. General information

All reagents for synthesis were bought commercially and used without further purification. Reactions were monitored with thin layer chromatography (TLC) on Merck Silica Gel F_{254} plates. NMR spectra were recorded using a Bruker Ascend 500 spectrometer at 293 K. All chemical shifts were referenced relative to the relevant deuterated solvent residual peaks or TMS. Assignments of the NMR spectra were deduced using ^{1}H NMR and ^{13}C NMR, along with 2D experiments (COSY, HSQC and HMBC). Due to poor solubility, ^{13}C NMR spectra could not be obtained for three compounds, **2c**, **2f**, and **2j**. The following abbreviations were used to explain the observed multiplicities: s (singlet), d (doublet), t (triplet), q (quartet), m (multiplet), bs (broad singlet), pt (pseudo triplet). Flash chromatography was performed with Merck Silica Gel 60. Microwave reactions were carried out using a CEM Discover Microwave Synthesizer with a vertically focused floor mounted infrared temperature sensor, external to the microwave tube. The 10 mL reaction vessels used were supplied from CEM and were made of borosilicate glass. High resolution mass spectrometry (HRMS) was performed on an Agilent-LC 1200 Series coupled to a 6210 or 6530 Agilent Time-Of-Flight (TOF) mass spectrometer equipped with an electrospray source in both positive and negative (ESI+/−) modes. Infrared spectra were obtained as KBr disks in the region 4000–400 cm^{-1} on a Perkin Elmer Spectrum 100 FT-IR spectrophotometer.

1.2. General procedure of microwave synthesis of pyrazoles

A microwave tube was charged with ketonitrile (2.0 mmol), methanol (1 mL), and hydrazine monohydrate (2.6 mmol) and subjected to microwave irradiation (100 W, 150 °C) for 5 minutes. Volatiles were subsequently removed under reduced pressure. The residue was purified by either trituration with cold methanol or cyclohexane, or by using column chromatography to give the final product.

1.2.1. 3-Phenyl-1*H*-pyrazol-5-amine (2a)

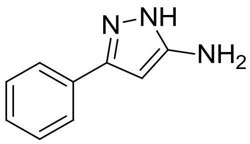

Microwave, 5 mins: 3-Phenyl-1*H*-pyrazol-5-amine (**2a**) was prepared as per the general procedure and purified by column chromatography (1:1 EtOAc: Petroleum Ether). White solid; Yield (0.316 g, 99%); R_f = 0.42 (7:3 EtOAc: Petroleum Ether); ^{1}H NMR (500 MHz, $CDCl_3$): δ 7.59 – 7.47 (m, 2H, Ar), 7.46 – 7.28 (m, 3H, Ar), 5.91 (s, 1H, CH); ^{13}C NMR (126 MHz, $CDCl_3$): δ 154.5 (quaternary), 145.6 (quaternary), 130.3 (quaternary), 128.9, 128.3, 125.4, 90.5; HRMS calcd for $C_9H_{10}N_3$ $[M + H]^+$: 160.0869, found 160.0872. Matches literature data[1].

Reflux, 17 hours: 3-Phenyl-1*H*-pyrazol-5-amine (**2a**) was prepared by heating benzoylacetonitrile (0.29 g, 2.0 mmol), hydrazine monohydrate (0.13 g, 2.6 mmol), and MeOH (2 mL) in a round-bottom flask at reflux for 17 hours. Volatiles were removed under reduced pressure and the product purified by column chromatography. Yield (0.191 g, 60%).

Reflux, 5 min: 3-Phenyl-1*H*-pyrazol-5-amine (**2a**) was prepared by heating benzoylacetonitrile (0.29 g, 2.0 mmol), hydrazine monohydrate (0.13 g, 2.6 mmol), and MeOH (2 mL) in a round-bottom flask at reflux for 5 minutes. Volatiles subsequently removed under reduced pressure and the product purified by column chromatography. Yield (0.094 g, 30%).

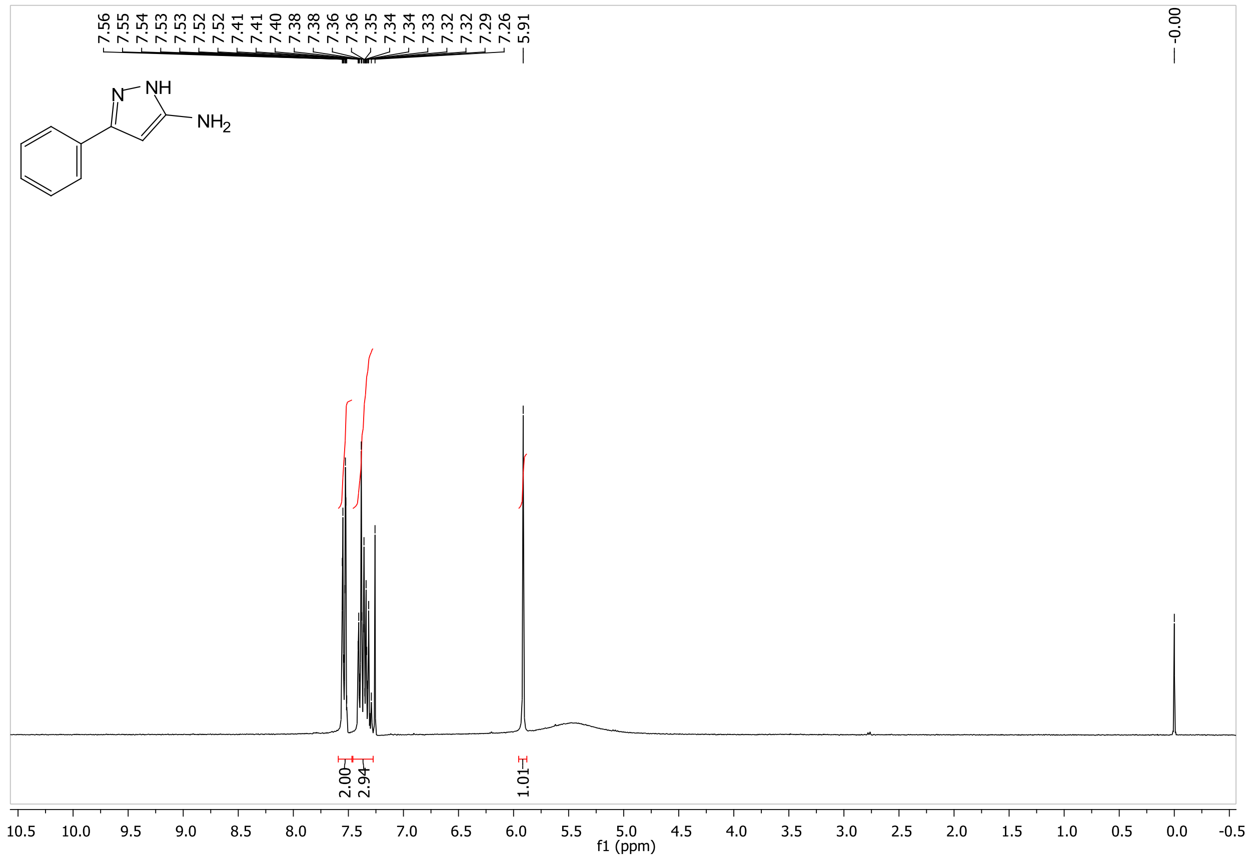

Figure S1. ^{1}H NMR spectrum of 3-phenyl-1*H*-pyrazol-5-amine (**2a**)

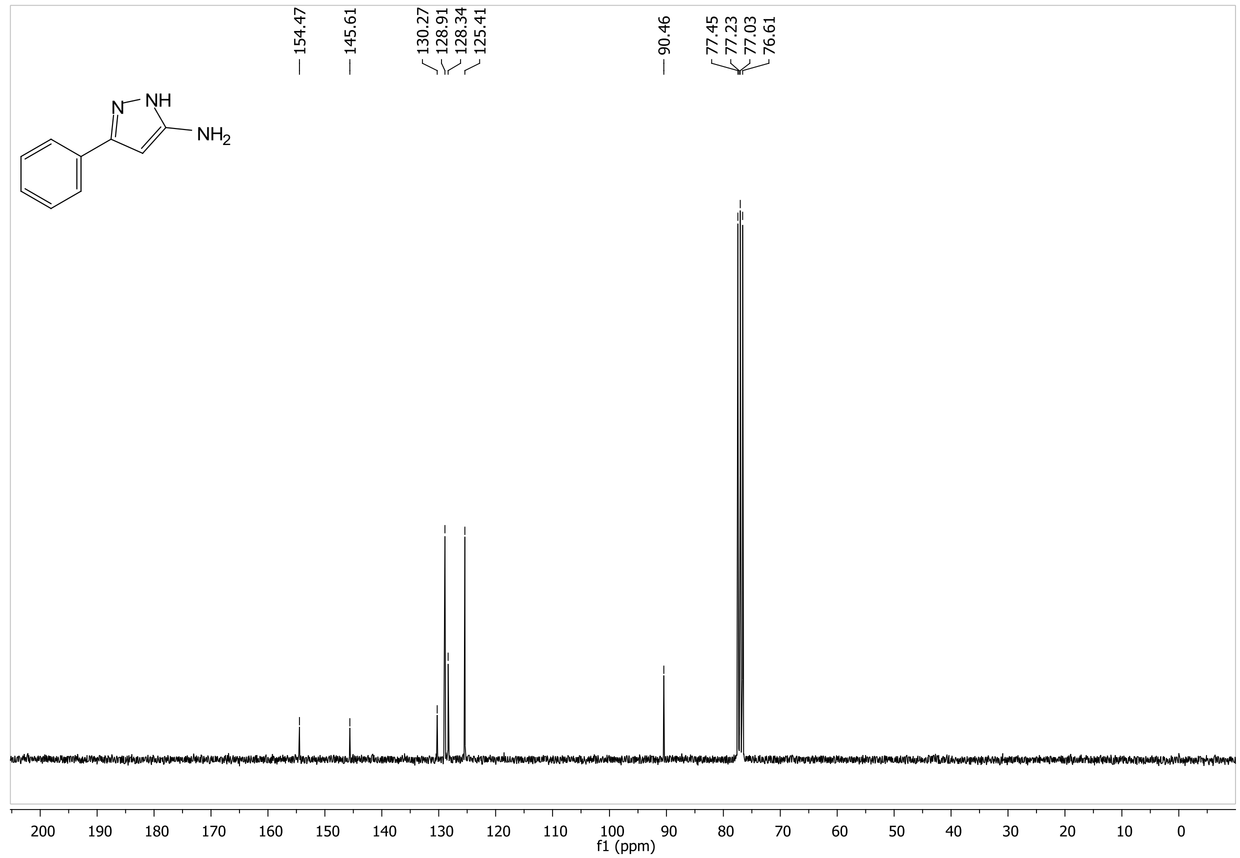

Figure S2. ^{13}C NMR spectrum of 3-phenyl-1*H*-pyrazol-5-amine (**2a**)

1.2.2. 3-(3-Chlorophenyl)-1*H*-pyrazol-5-amine (2b)

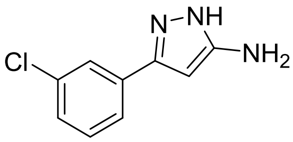

3-(3-Chlorophenyl)-1*H*-pyrazol-5-amine (**2b**) was prepared as per general procedure and purified by column chromatography (1:1 EtOAc:Petroleum ether). Light red solid; Yield (0.364 g, 94%); R_f = 0.11 (1:1 EtOAc:Petroluem ether); ^{1}H NMR (500 MHz, $CDCl_3$) δ 7.53 (m, 1H, Ar), 7.42 (dt, *J* = 7.2, 1.8 Hz, 1H, Ar), 7.35 – 7.27 (m, 2H, Ar), 5.91 (s, 1H, CH). ^{13}C NMR (126 MHz, $CDCl_3$) δ 154.0 (quaternary), 144.8 (quaternary), 134.9 (quaternary), 132.2 (quaternary), 130.2, 128.3, 125.5, 123.4, 90.8. IR (KBr) 3397, 3200, 3142, 1508. HRMS calcd for $C_9H_9ClN_3$ $[M + H]^+$: 194.0480, found 194.0480. Matches literature data[2,4].

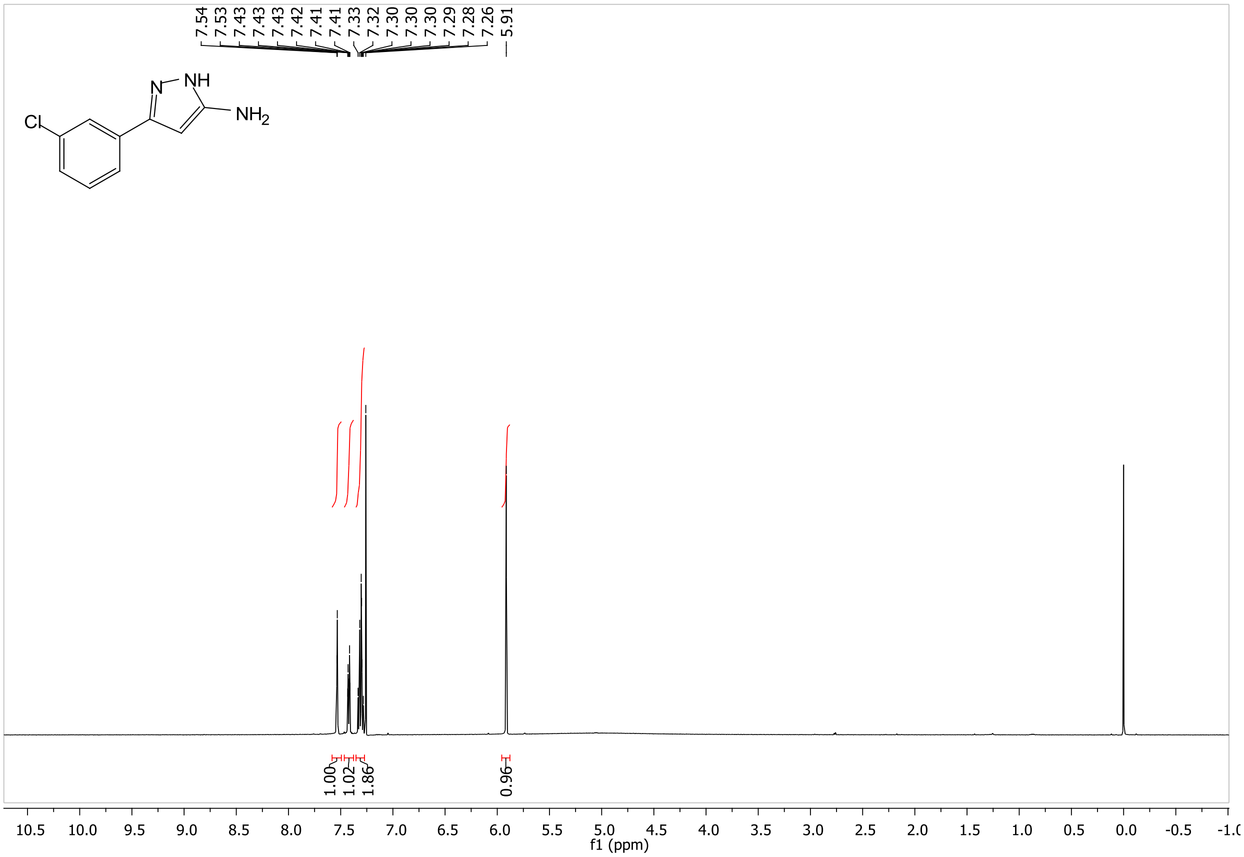

Figure S3. ^{1}H NMR spectrum of 3-(3-chlorophenyl)-1*H*-pyrazol-5-amine (**2b**)

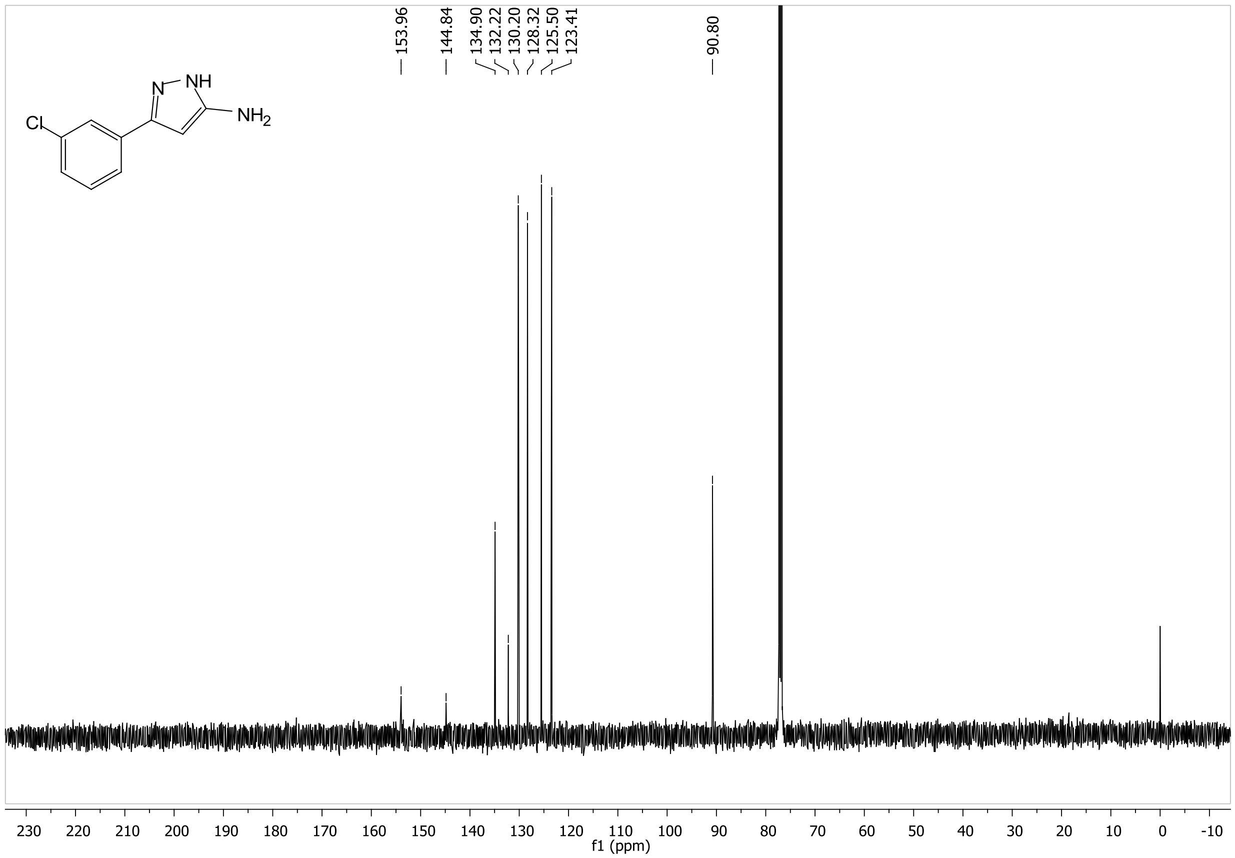

Figure S4. ^{13}C NMR spectrum of 3-(3-chlorophenyl)-1H-pyrazol-5-amine (**2b**)

1.2.3. 3-(4-Chlorophenyl)-1*H*-pyrazol-5-amine (2c)

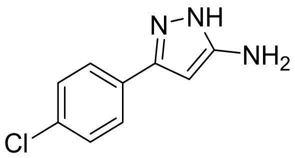

3-(4-Chlorophenyl)-1H-pyrazol-5-amine (**2c**) was prepared as per general procedure and purified by trituration with cold MeOH. Red solid; Yield (0.191 g, 49%); ^{1}H NMR (500 MHz, DMSO) δ 7.67 (d, *J* = 8.6 Hz, 2H, Ar), 7.42 (d, *J* = 8.6 Hz, 2H, Ar), 5.76 (s, 1H, CH), 4.87 (s, 2H, NH_2). HRMS calcd for $C_9H_9ClN_3$ $[M + H]^+$: 194.0480, found 194.0482. IR: 3398, 3138, 1615, 1508. Matches literature data[2].

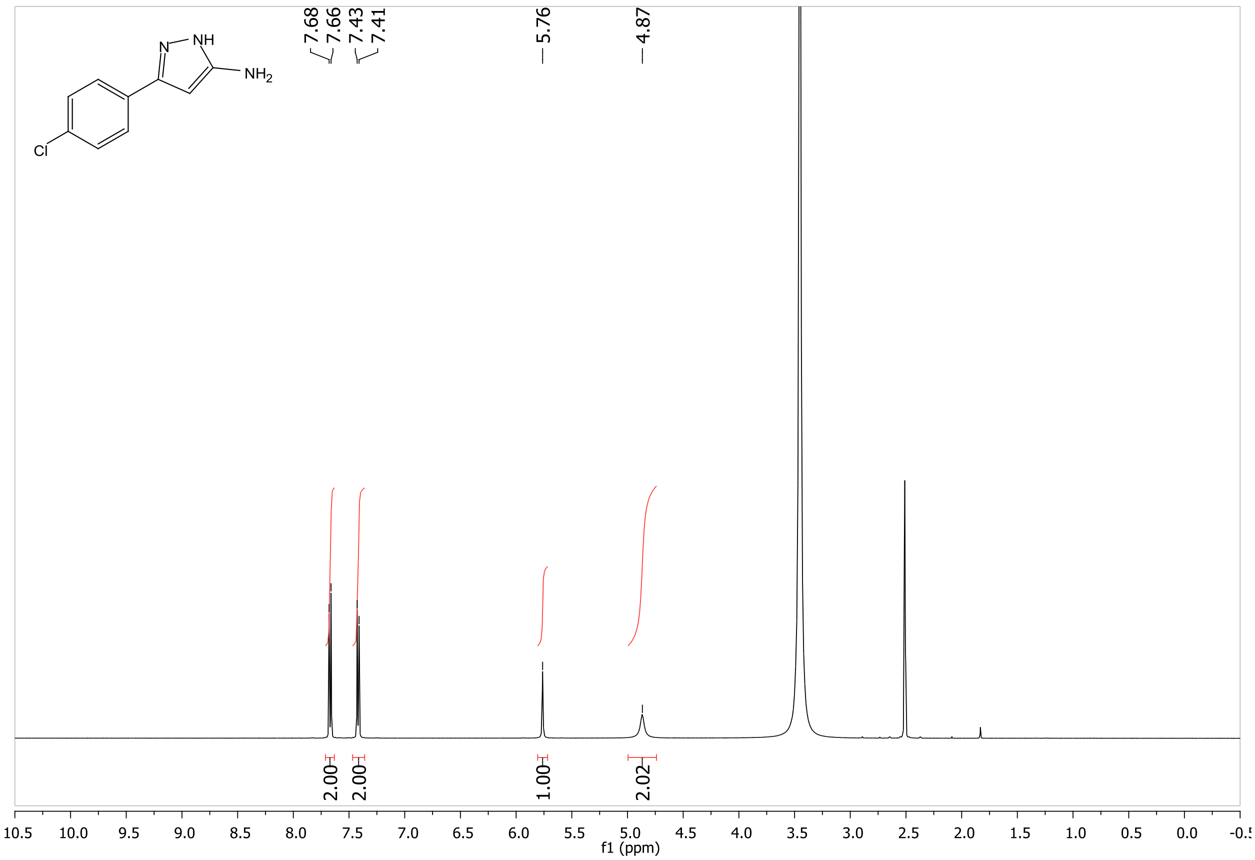

Figure S5. ^{1}H NMR spectrum of 3-(4-chlorophenyl)-1H-pyrazol-5-amine (**2c**)

1.2.4. 3-(2-Methoxyphenyl)-1*H*-pyrazol-5-amine (2d)

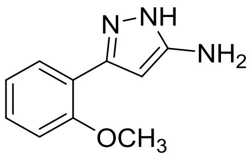

3-(2-Methoxyphenyl)-1H-pyrazol-5-amine (**2d**) was prepared as per general procedure and purified by trituration with cyclohexane. Light yellow solid; Yield (0.322 g, 85%);^{1}H NMR (500 MHz, DMSO) δ 7.63 (dd, *J* = 7.7, 1.4 Hz, 1H, Ar), 7.31 – 7.24 (m, 1H, Ar), 7.08 (d, *J* = 8.0 Hz, 1H, Ar), 6.97 (td, *J* = 7.6, 1.0 Hz, 1H, Ar), 5.90 (s, 1H, CH), 4.59 (bs, 2H, NH_2), 3.85 (s, 3H, CH_3). ^{13}C NMR (126 MHz, DMSO) δ 156.1 (quaternary), 154.4 (quaternary), 140.8 (quaternary), 129.1, 127.7, 121.0, 119.8 (quaternary), 112.3, 91.7, 55.9. HRMS calcd for $C_{10}H_{12}N_3O$ $[M + H]^+$: 190.0975, found 190.0980. Matches literature data[3].

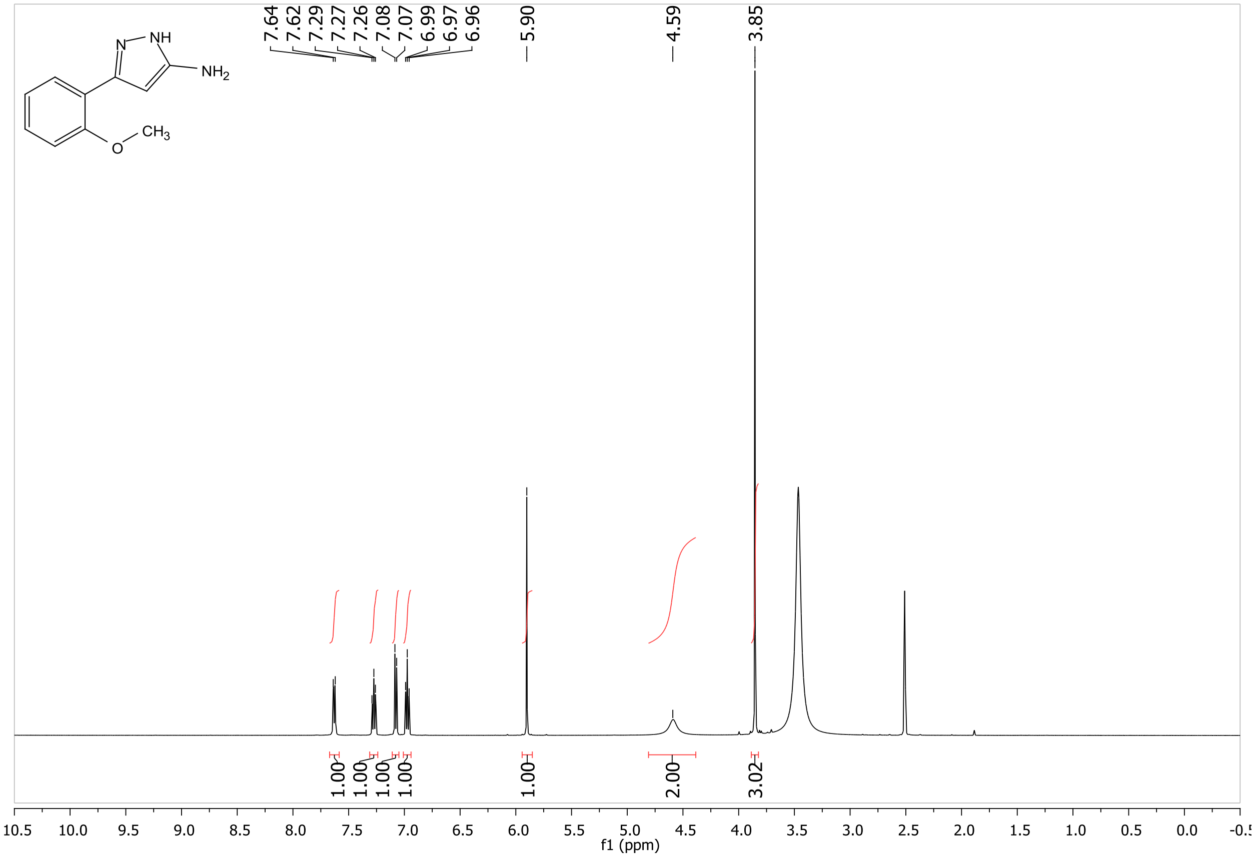

Figure S6. ^{1}H NMR spectrum of 3-(2-methoxyphenyl)-1*H*-pyrazol-5-amine (**2d**)

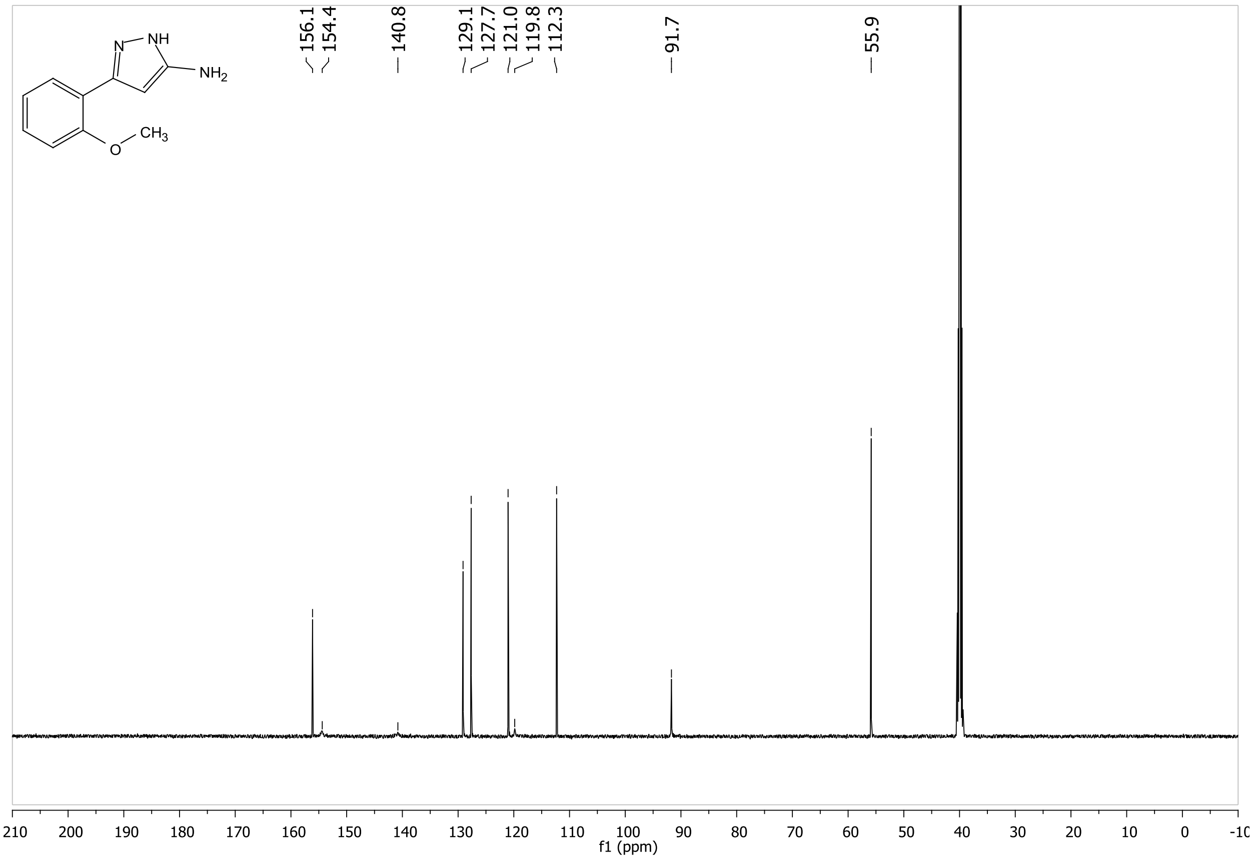

Figure S7. ^{13}C NMR spectrum of 3-(2-methoxyphenyl)-1*H*-pyrazol-5-amine (**2d**)

1.2.5. 3-(3-Methoxyphenyl)-1*H*-pyrazol-5-amine (2e)

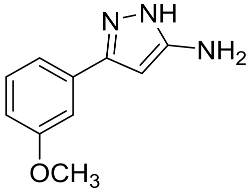

3-(3-Methoxyphenyl)-1*H*-pyrazol-5-amine (**2e**) was prepared as per general procedure and purified by column chromatography (3:2 EtOAc:Petroleum Ether). Red solid; Yield (0.278 g, 74%); R_f 0.18 (3:2 EtOAc:Petroleum Ether); ^{1}H NMR (500 MHz, $CDCl_3$) δ 7.18 (pseudo t, *J* = 8.2 Hz, 1H, Ar), 7.12 – 7.07 (m, 2H, Ar), 6.80 – 6.76 (m, 1H, Ar), 5.79 (s, 1H. CH), 3.68 (s, 3H, OCH_3). ^{13}C NMR (126 MHz, $CDCl_3$) δ 159.8 (quaternary), 153.8 (quaternary), 146.1 (quaternary), 131.9 (quaternary), 129.9, 118.0, 113.9, 110.9, 90.1, 55.2. HRMS calcd for $C_{10}H_{12}N_3O$ $[M + H]^+$: 190.0975, found 190.0979. Matches literature data[4].

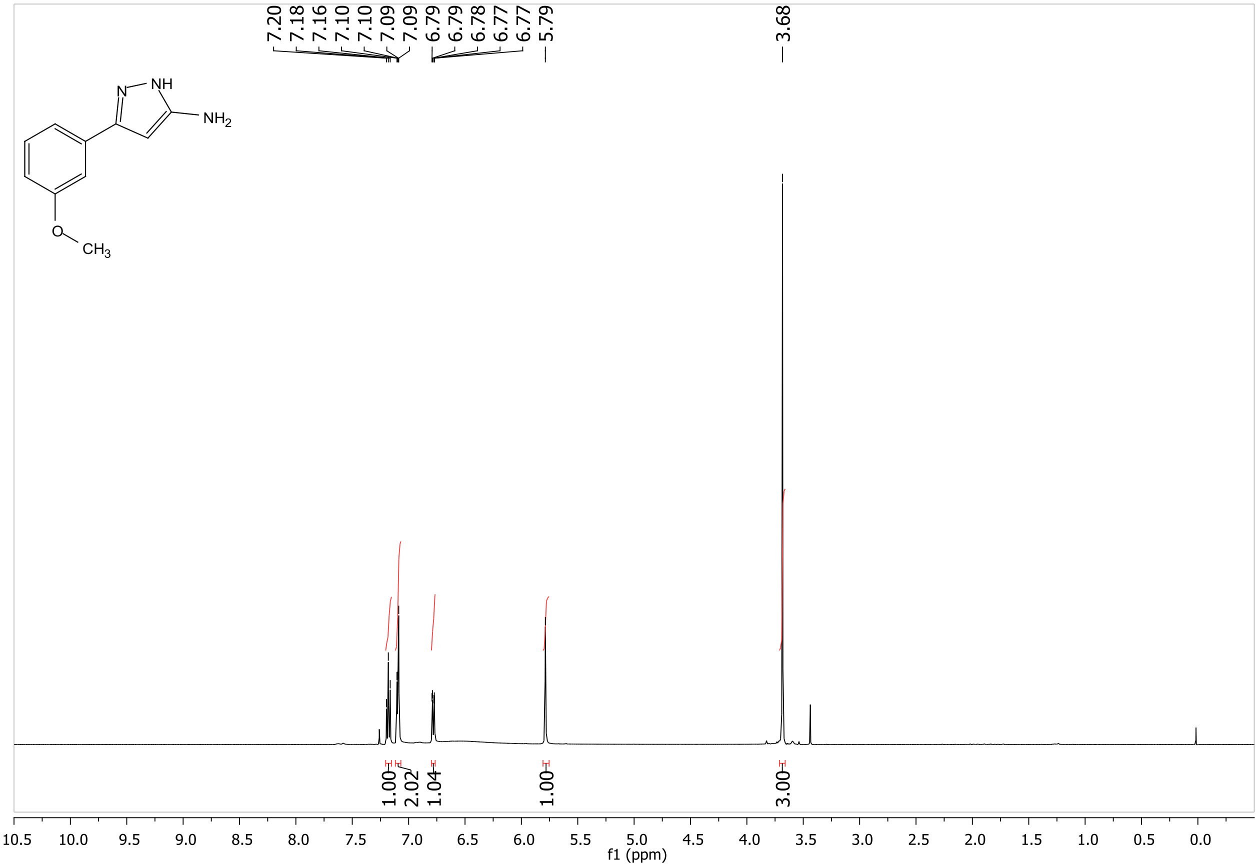

Figure S8. ^{1}H NMR spectrum of 3-(3-methoxyphenyl)-1*H*-pyrazol-5-amine (**2e**)

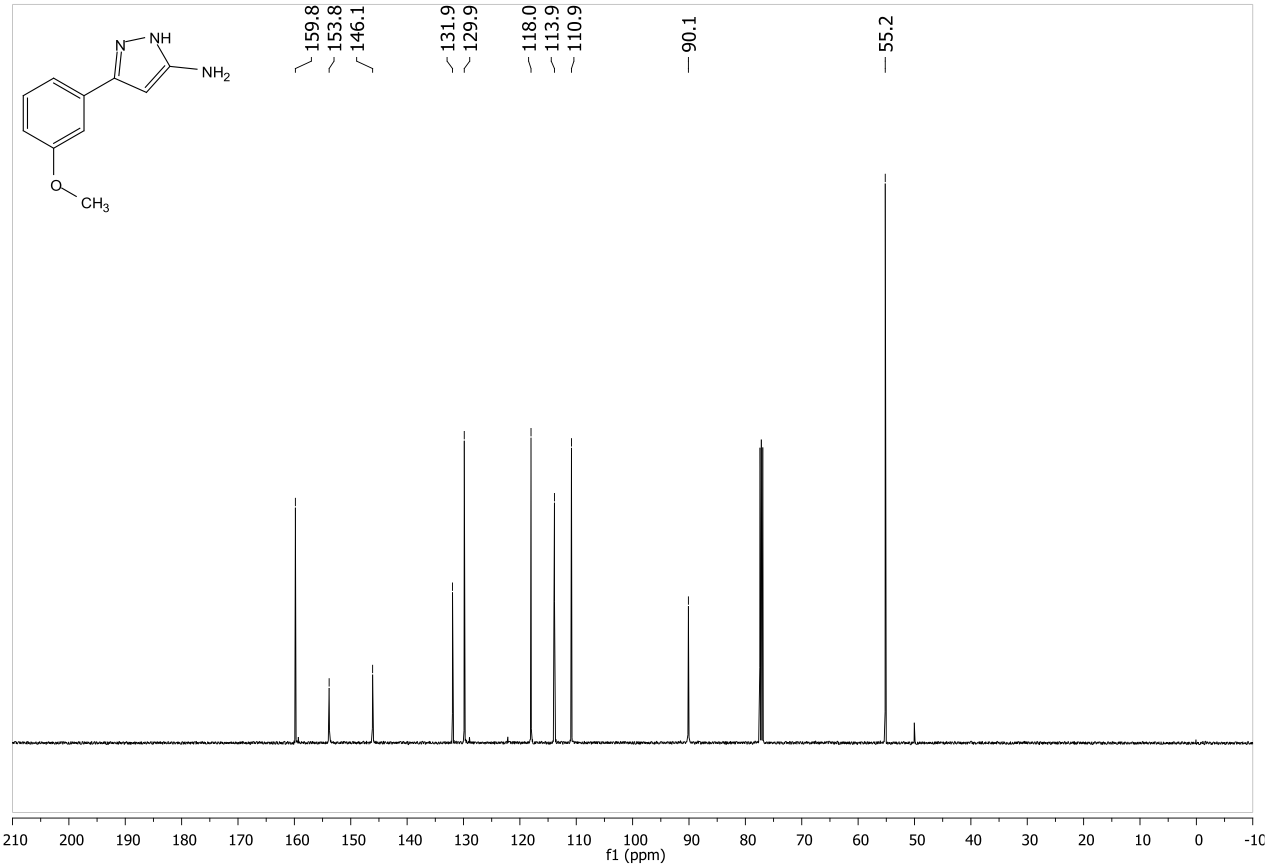

Figure S9. ^{13}C NMR spectrum of 3-(3-methoxyphenyl)-1*H*-pyrazol-5-amine (**2e**)

1.2.6. 3-(4-Methoxyphenyl)-1*H*-pyrazol-5-amine (2f)

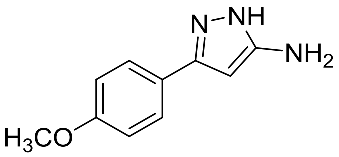

3-(4-Methoxyphenyl)-1*H*-pyrazol-5-amine (**2f**) was prepared as per general procedure and purified by trituration with cold MeOH. Light yellow solid; Yield (0.227 g, 60%); ^{1}H NMR (500 MHz, DMSO) δ 7.57 (d, *J* = 8.8 Hz, 2H, Ar), 6.94 (d, *J* = 8.8 Hz, 2H, Ar), 5.69 (s, 1H,CH), 4.66 (s, 2H, NH_2), 3.77 (s, 3H, OCH_3). HRMS calcd for $C_{10}H_{12}N_3O$ $[M + H]^+$: 190.0975, found 190.0981. IR (KBr) 3435, 3201, 1606, 1499. Matches literature data[2].

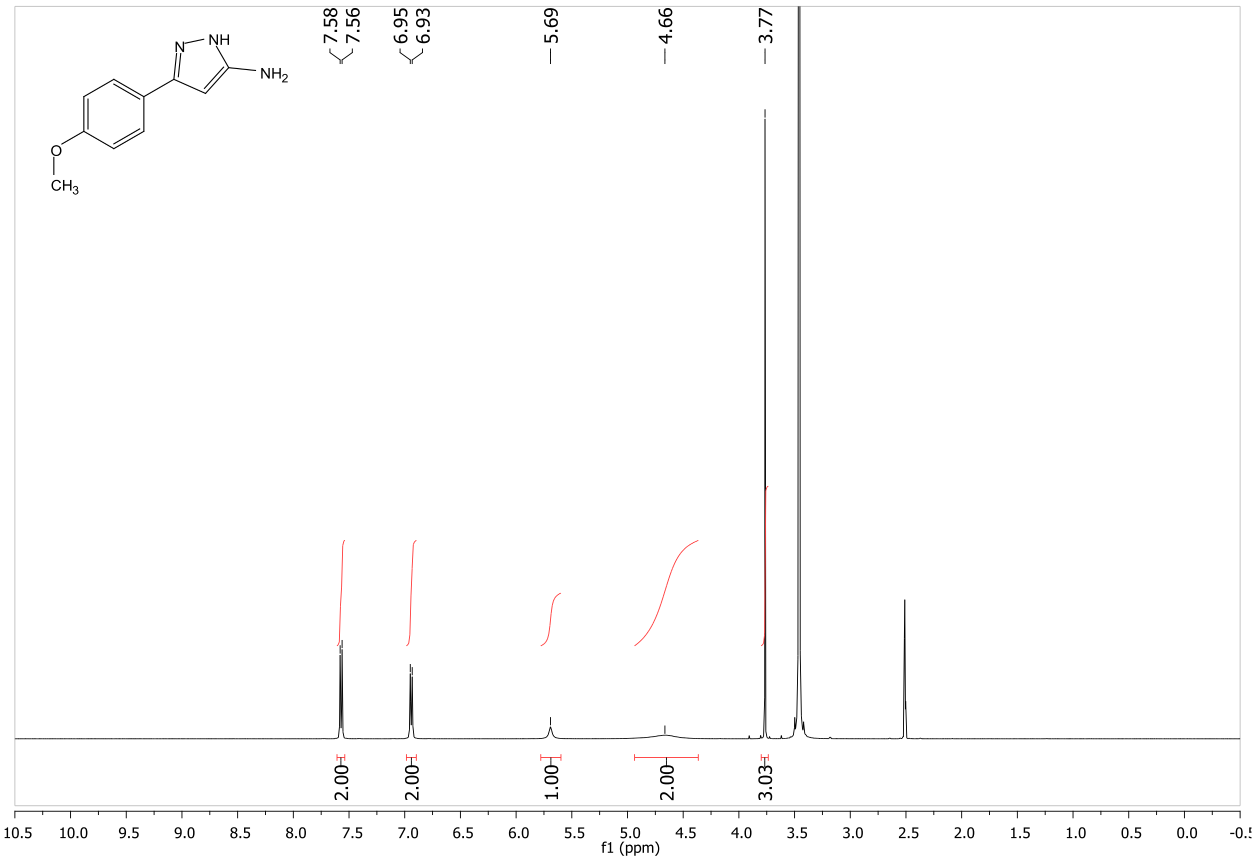

Figure S10. ^{1}H NMR spectrum of 3-(4-methoxyphenyl)-1*H*-pyrazol-5-amine (**2f**)

1.2.7. 3-(4-Fluorophenyl)-1*H*-pyrazol-5-amine (2g)

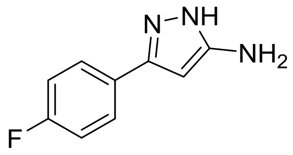

3-(4-Fluorophenyl)-1*H*-pyrazol-5-amine (**2g**) was prepared as per general procedure and purified by trituration with cyclohexane. Light brown solid; Yield (0.339 g, 96%); ^{1}H NMR (500 MHz, $CDCl_3$) δ 7.58 – 7.47 (m, 2H, Ar), 7.12 – 7.00 (m, 2H, Ar), 5.85 (s, 1H, CH). ^{13}C NMR (126 MHz, $CDCl_3$) δ 162.7 (quaternary, d, J_{CF} = 248.6 Hz, CF), 154.0 (quaternary), 145.4 (quaternary), 127.2 (d, J_{CF} = 8.2 Hz,), 126.8 (quaternary, d, J_{CF} = 3.1 Hz), 115.9 (d, J_{CF} = 21.8 Hz), 90.3. HRMS calcd for $C_9H_9FN_3$ $[M + H]^+$: 178.0775, found 178.0770. Matches literature data[2].

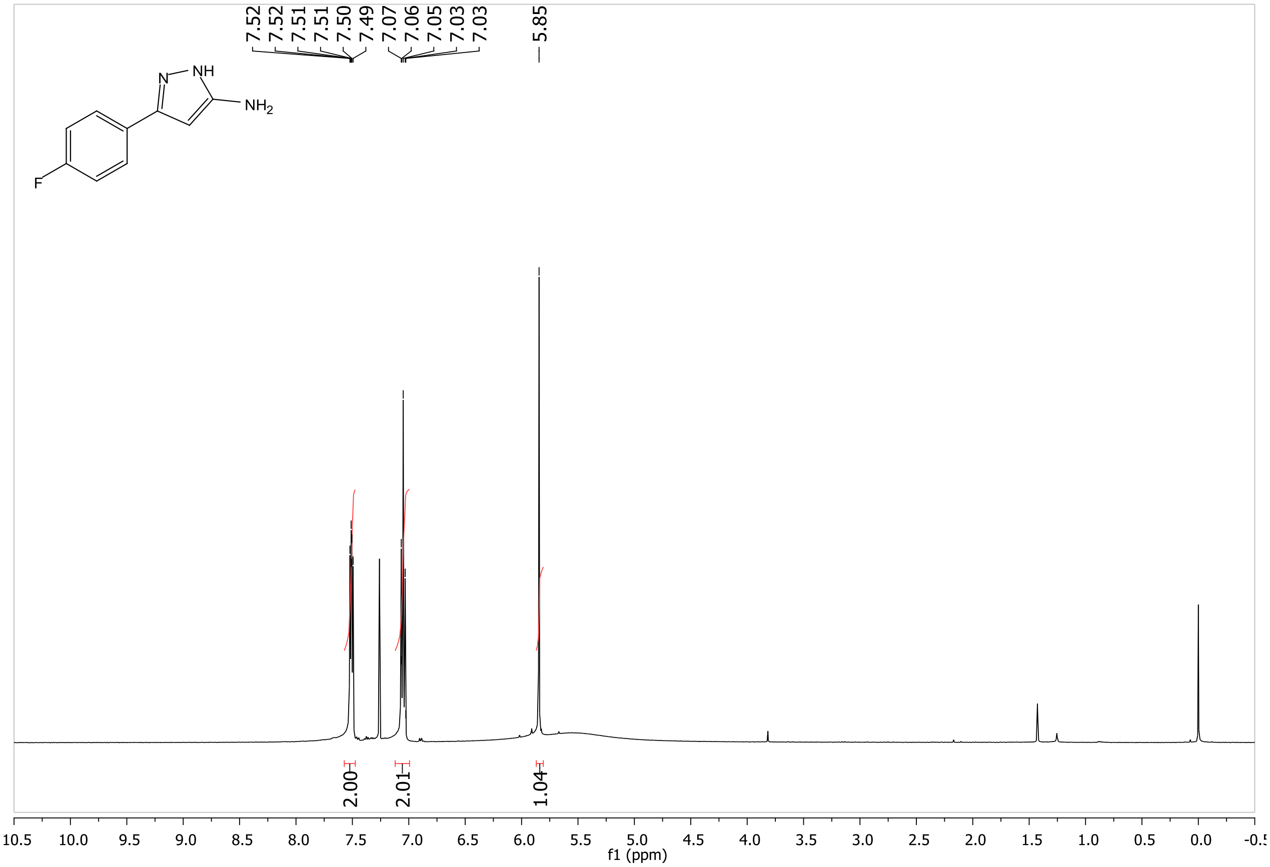

Figure S11. ^{1}H NMR spectrum of 3-(4-fluorophenyl)-1*H*-pyrazol-5-amine (**2g**)

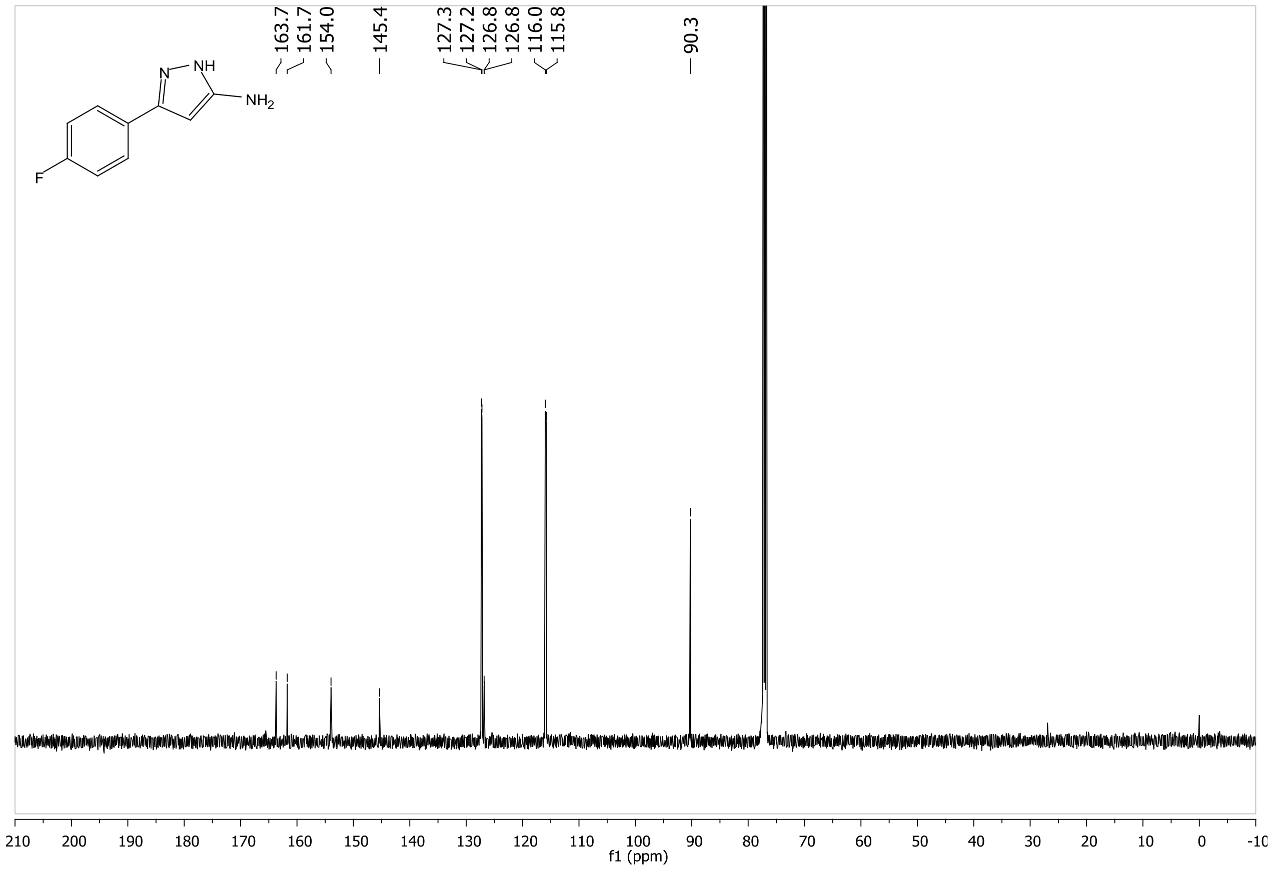

Figure S12. ^{13}C NMR spectrum of 3-(4-fluorophenyl)-1*H*-pyrazol-5-amine (**2g**)

1.2.8. 3-(3-Nitrophenyl)-1*H*-pyrazol-5-amine (2h)

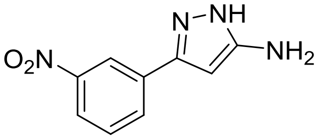

3-(3-Nitrophenyl)-1*H*-pyrazol-5-amine (**2h**) was prepared as per general procedure and purified by column chromatography (3:2 EtOAc:Petroleum Ether). Yellow solid; Yield (0.137 g, 67%); R_f = 0.1 (3:2 EtOAc:Petroleum Ether); ^{1}H NMR (500 MHz, MeOD) δ 8.47 (pseudo t, *J* = 1.8 Hz, 1H, Ar), 8.10 (ddd, *J* = 8.2, 2.3, 1.0 Hz, 1H, Ar), 7.98 (ddd, *J* = 7.9, 1.6, 1.0 Hz, 1H, Ar), 7.57 (pseudo t, *J* = 7.9 Hz, 1H, Ar), 5.98 (s, 1H, CH). ^{13}C NMR (126 MHz, MeOD) δ 152.3 (quaternary), 148.6 (quaternary), 146.3 (quaternary), 133.8 (quaternary), 130.8, 129.6, 121.8, 119.4, 88.3. HRMS calcd for $C_9H_9N_4O_2$ $[M + H]^+$: 205.0720, found 205.0729. Matches literature data[5].

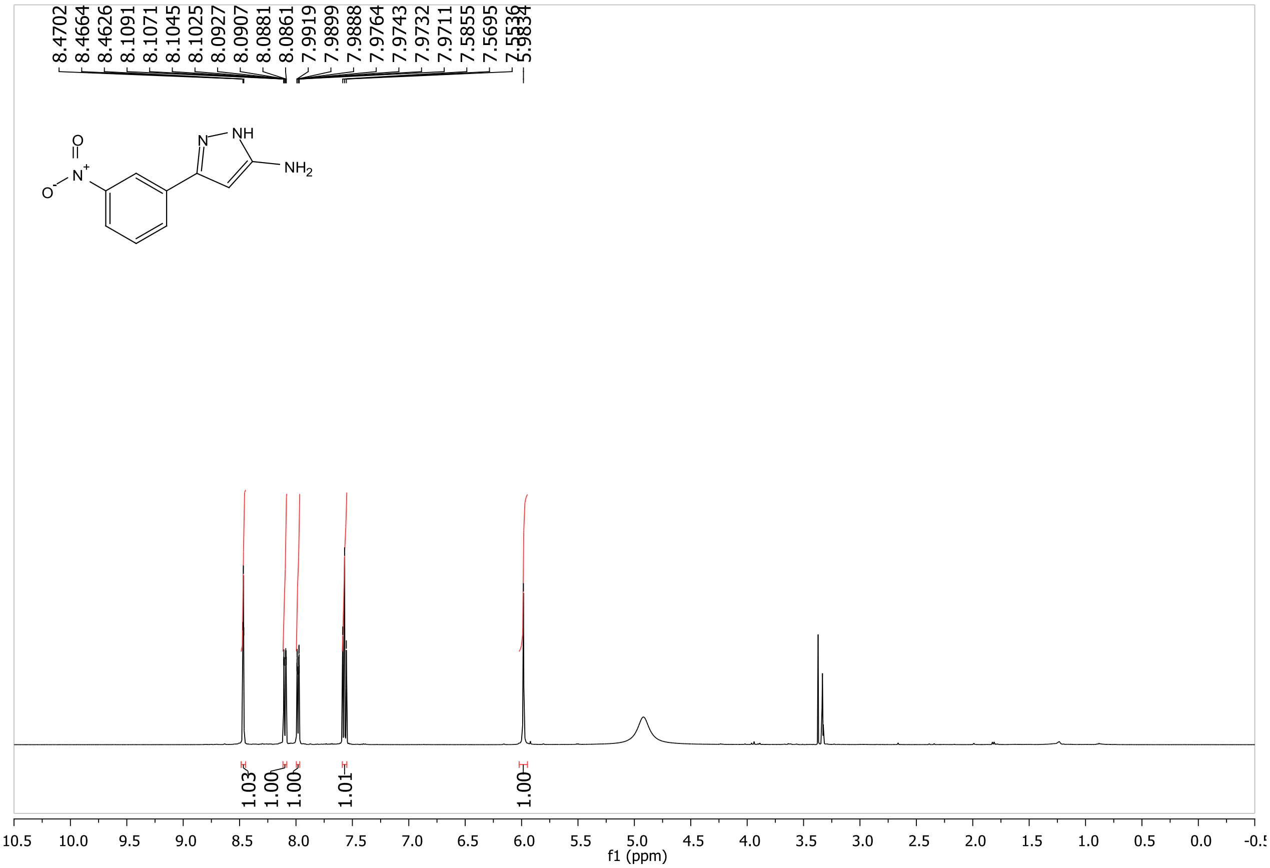

Figure S13. ^{1}H NMR spectrum of 3-(3-nitrophenyl)-1*H*-pyrazol-5-amine (**2h**)

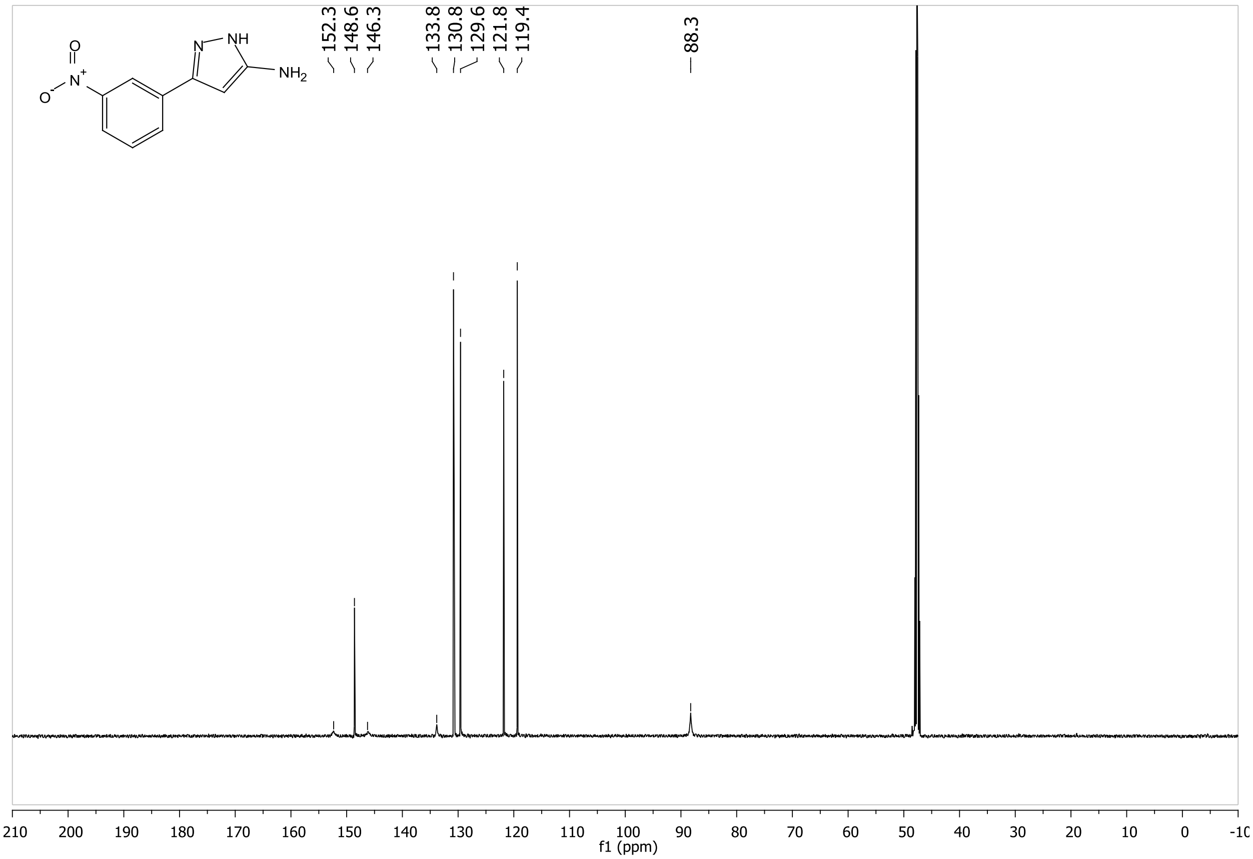

Figure S14. ^{13}C NMR spectrum of 3-(3-nitrophenyl)-1*H*-pyrazol-5-amine (**2h**)

1.2.9. 3-(*p*-Tolyl)-1*H*-pyrazol-5-amine (2i)

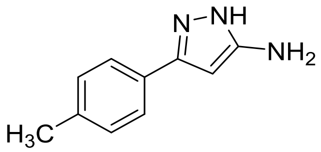

3-(*p*-Tolyl)-1*H*-pyrazol-5-amine (**2i**) was prepared as per general procedure and purified by trituration with cyclohexane. Light brown solid; Yield (0.241 g, 70%); ^{1}H NMR (500 MHz, $CDCl_3$) δ 7.42 (d, *J* = 8.1 Hz, 2H, Ar), 7.20 (d, *J* = 8.1 Hz, 2H, Ar), 5.88 (s, 1H, CH), 2.36 (s, 3H, CH_3). ^{13}C NMR (126 MHz, $CDCl_3$) δ 154.8 (quaternary), 145.4 (quaternary), 138.4 (quaternary), 129.6, 127.3 (quaternary), 125.3, 90.4, 21.3. IR (KBr) 3413, 3360, 3315, 1519. HRMS calcd for $C_{10}H_{12}N_3$ $[M + H]^+$: 174.1026, found 174.1027. Matches literature data[2].

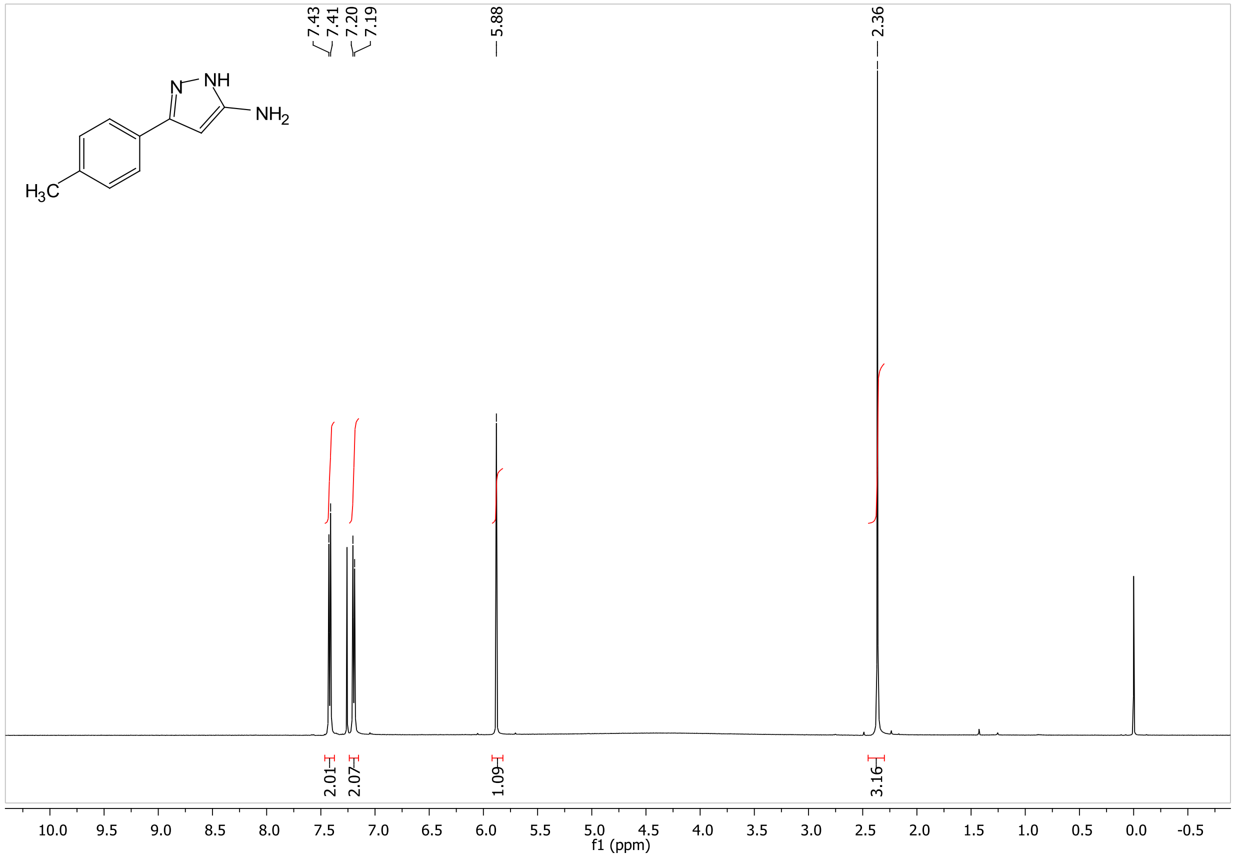

Figure S15. ^{1}H NMR spectrum of 3-(*p*-tolyl)-1*H*-pyrazol-5-amine (**2i**)

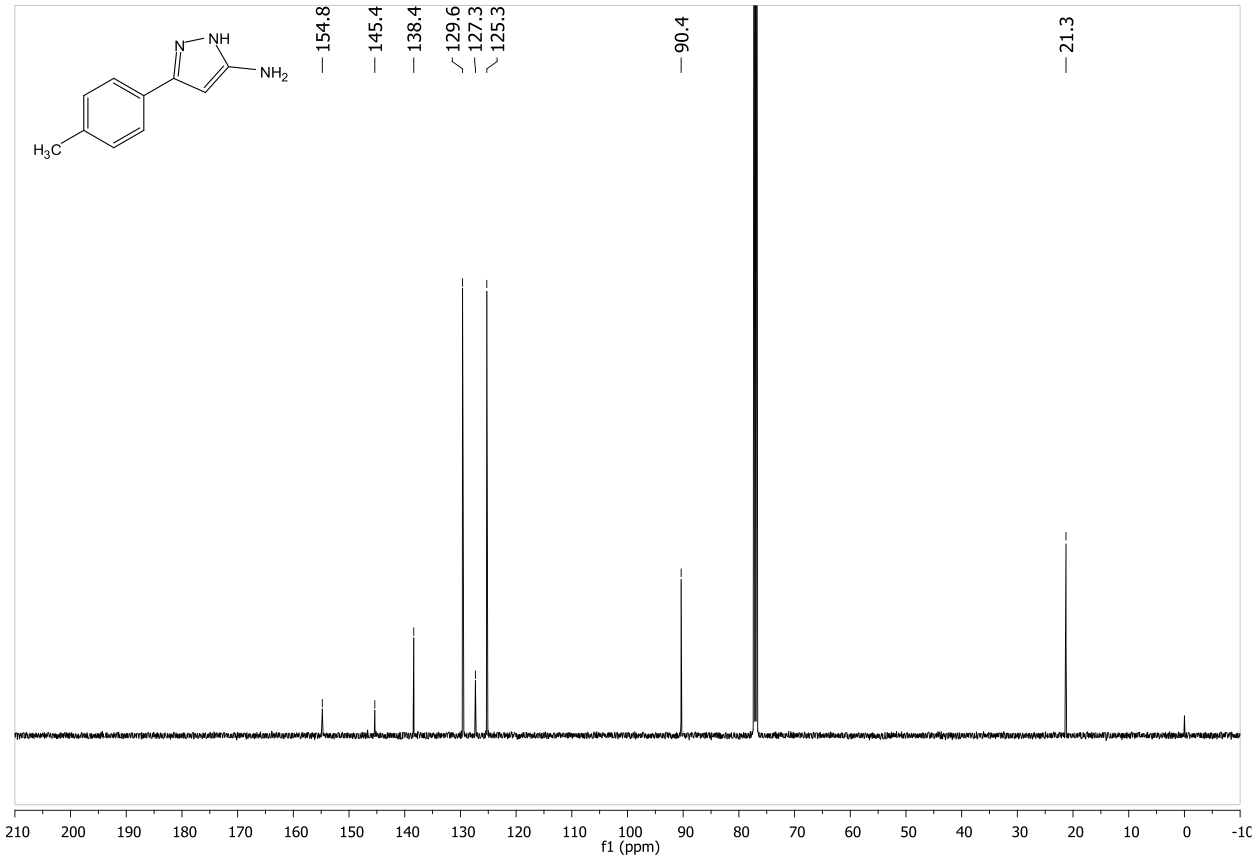

Figure S16. ^{13}C NMR spectrum of 3-(*p*-tolyl)-1*H*-pyrazol-5-amine (**2i**)

1.2.10. 3-(4-Bromophenyl)-1*H*-pyrazol-5-amine (2j)

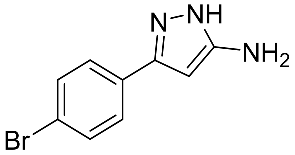

3-(4-Bromophenyl)-1*H*-pyrazol-5-amine (**2j**) was prepared as per general procedure and purified by column chromatography (3:2 EtOAc:Petroleum Ether). Yellow solid; Yield (0.279 g, 59%); R_f = 0.1 (3:2 EtOAc:Petroleum Ether); ^{1}H NMR (500 MHz, DMSO) δ 7.61 (d, *J* = 8.5 Hz, 2H, Ar), 7.55 (d, *J* = 8.5 Hz, 2H, Ar), 5.76 (s, 1H, CH), 4.88 (bs, 2H, NH_2). HRMS calcd for $C_9H_9BrN_3$ [M + H]$^+$: 237.9974, found 237.9973. IR (KBr) 3395, 3172, 1592, 1508, 1480. Matches literature data[2].

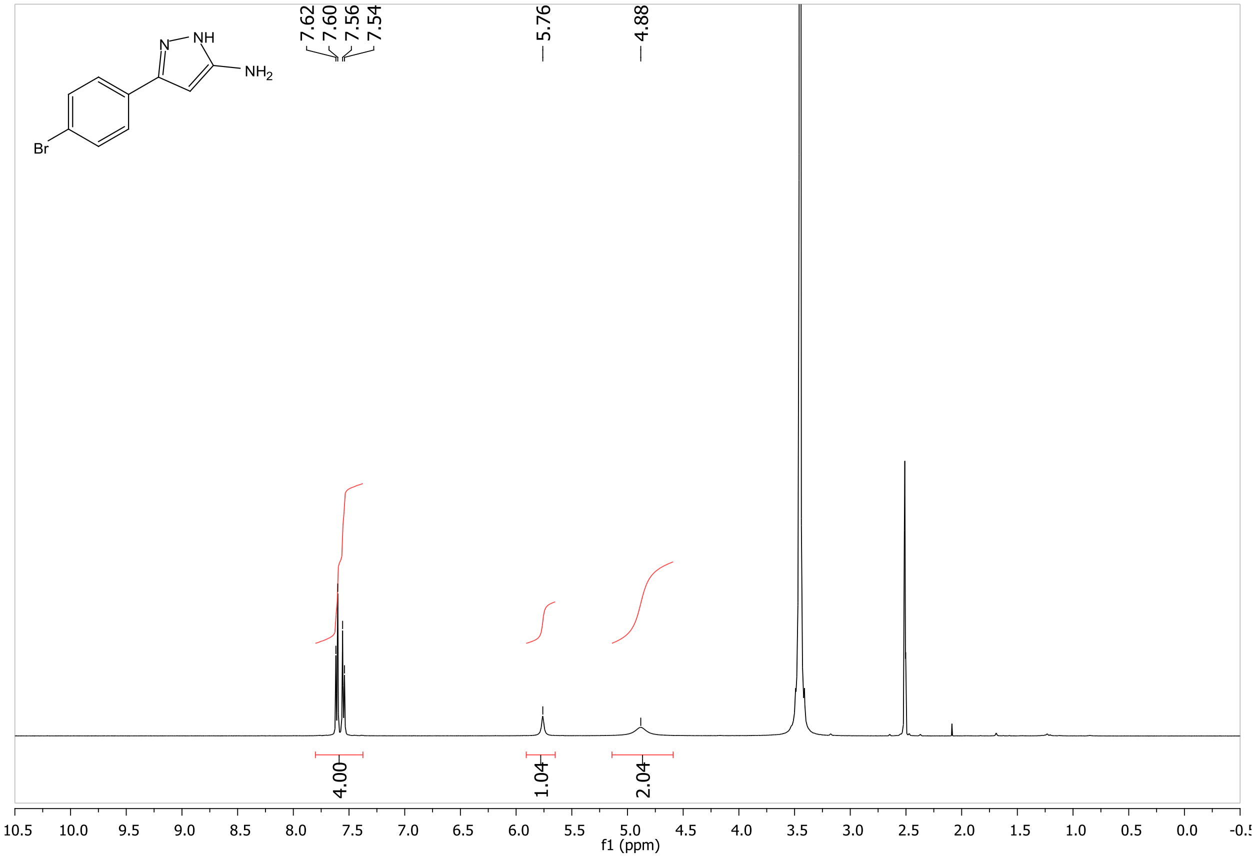

Figure S17. ^{1}H NMR spectrum of 3-(4-bromophenyl)-1*H*-pyrazol-5-amine (**2j**)

1.2.11. 3-(Furan-2-yl)-1*H*-pyrazol-5-amine (2k)

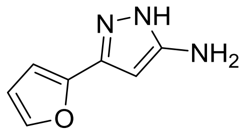

3-(Furan-2-yl)-1*H*-pyrazol-5-amine (**2k**) was prepared as per general procedure and purified by column chromatography (9:1 DCM: MeOH). Red Brown solid; Yield (0.223 g, 75%); R_f = 0.47 (9:1 DCM: MeOH); ^{1}H NMR (500 MHz, $CDCl_3$) δ 7.42 (d, *J* = 1.0 Hz, 1H, furan), 6.53 (d, *J* = 3.3 Hz, 1H, furan), 6.45 (dd, *J* = 3.3, 1.8 Hz, 1H, furan), 5.85 (s, 1H, CH). ^{13}C NMR (126 MHz, $CDCl_3$) δ 154.3 (quaternary), 145.5 (quaternary), 142.1, 136.7 (quaternary), 111.5, 106.4, 89.6. HRMS calcd for $C_7H_8N_3O$ $[M + H]^+$: 150.0662, found 150.0669. Matches literature data[6].

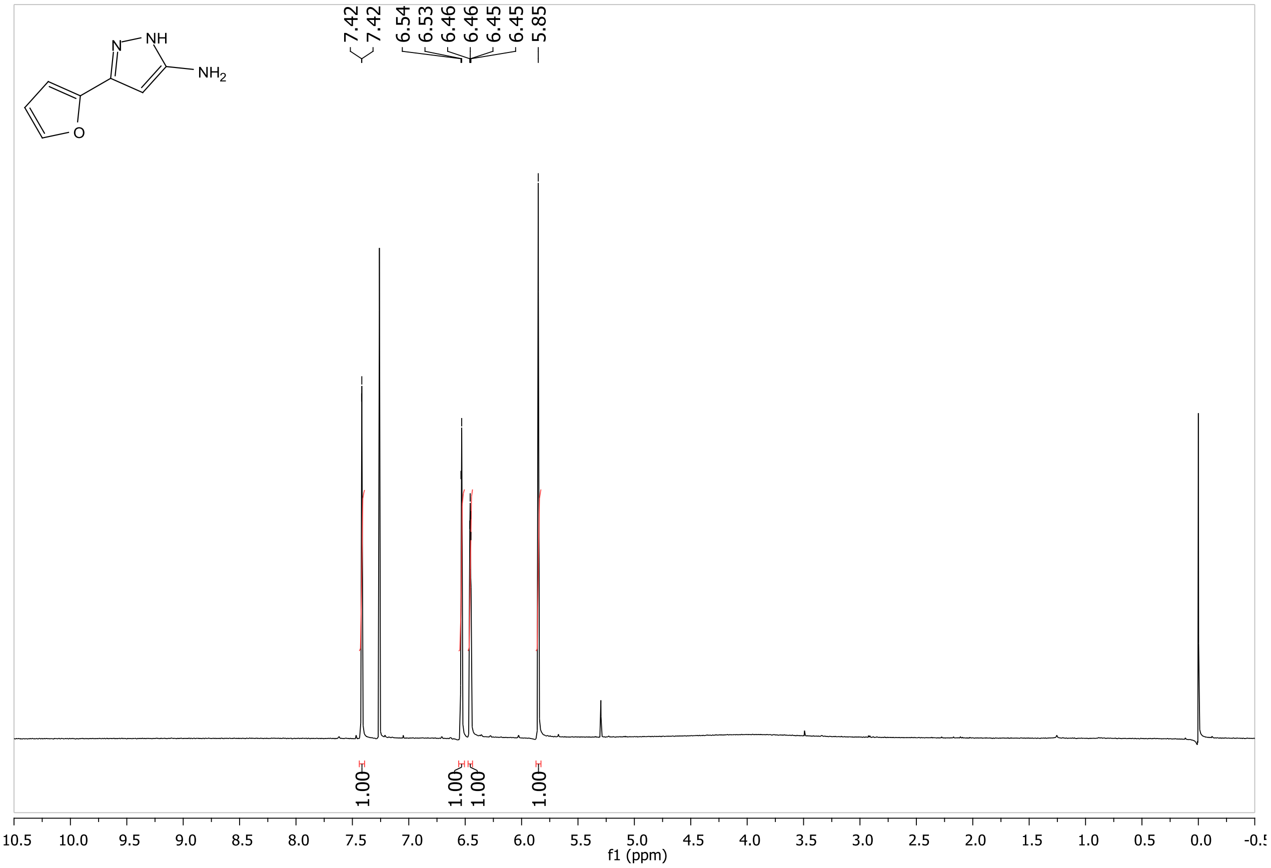

Figure S18. ^{1}H NMR spectrum of 3-(furan-2-yl)-1*H*-pyrazol-5-amine (**2k**)

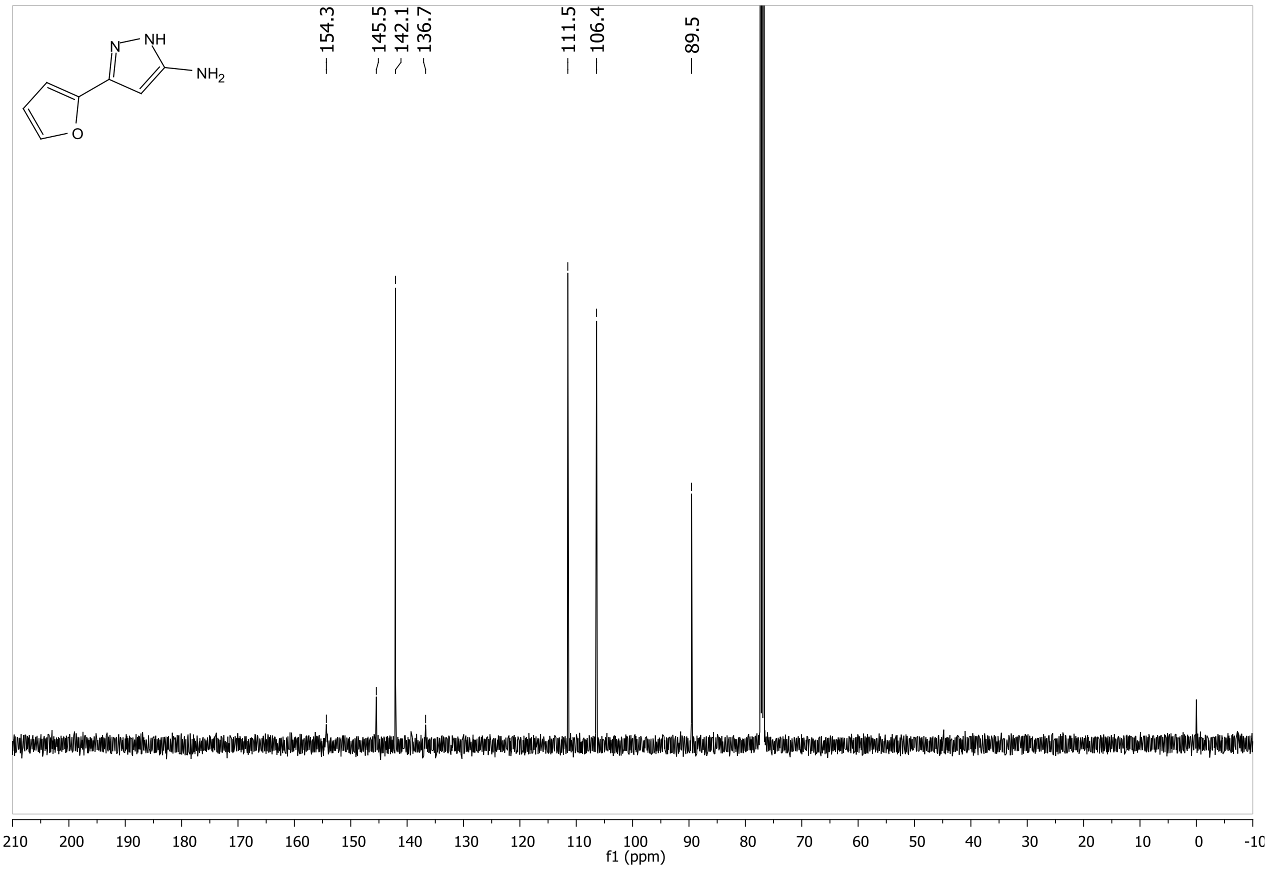

Figure S19. ^{13}C NMR spectrum of 3-(furan-2-yl)-1*H*-pyrazol-5-amine (**2k**)

1.2.12. 3-(Thiophenyl-2-yl)-1*H*-pyrazol-5-amine (2l)

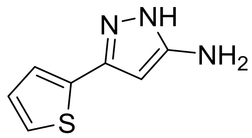

3-(Thiophenyl-2-yl)-1*H*-pyrazol-5-amine (**2l**) was prepared as per general procedure and purified by column chromatography (4:1 EtOAc:Petroleum Ether). Yellow solid; Yield (0.267 g, 81%); R_f 0.1 (4:1 EtOAc:Petroleum Ether); ^{1}H NMR (500 MHz, $CDCl_3$) δ 7.22 (dd, *J* = 5.1, 1.1 Hz, 1H, thiophene), 7.19 (dd, *J* = 3.6, 1.1 Hz, 1H, thiophene), 6.99 (dd, *J* = 5.1, 3.6 Hz, 1H, thjophene), 5.78 (s, 1H, CH). ^{13}C NMR (126 MHz, $CDCl_3$) δ 152.8 (quaternary), 141.6 (quaternary), 133.6 (quaternary), 127.7, 124.9, 124.1, 90.1. HRMS calcd for $C_7H_8N_3S$ $[M + H]^+$: 166.0433, found 166.043. Matches literature data[7].

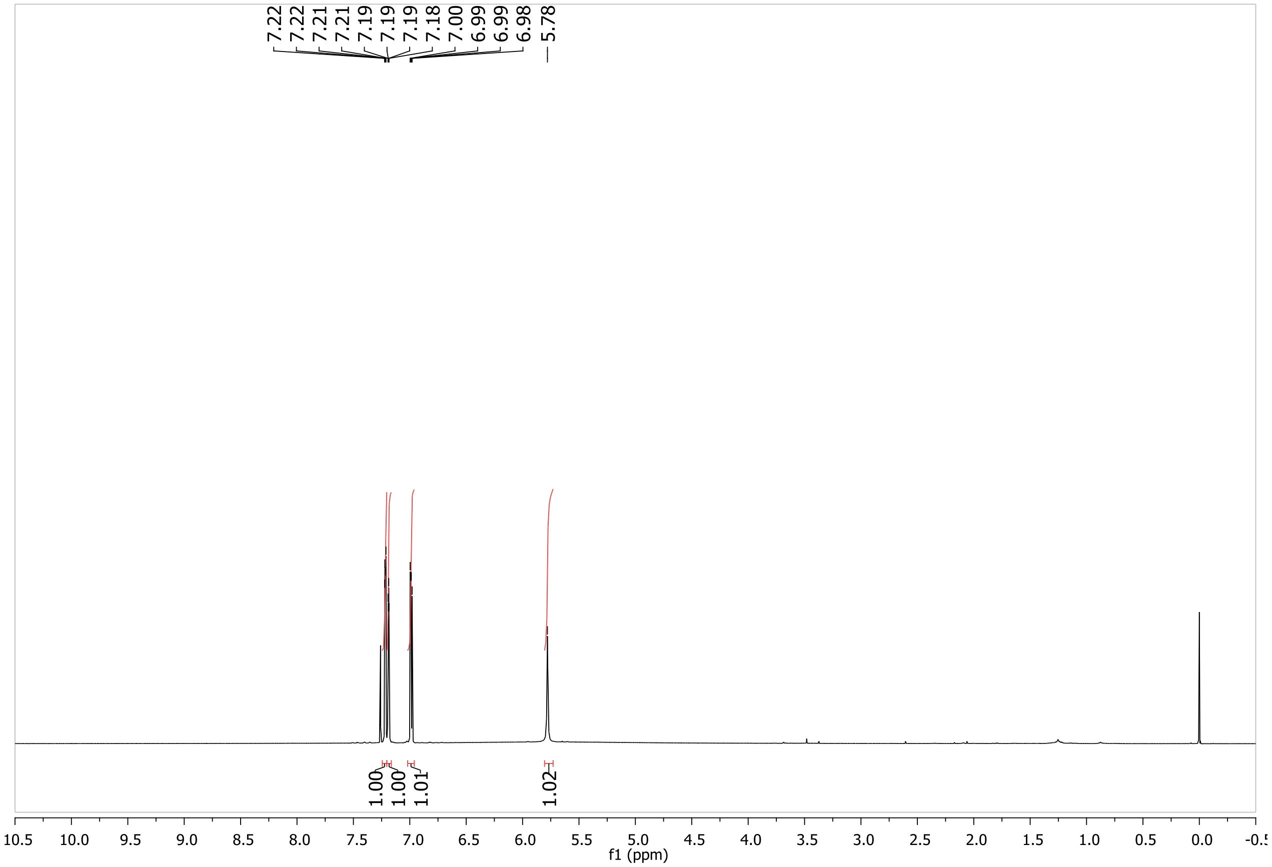

Figure S20. ^{1}H NMR spectrum of 3-(thiophenyl-2-yl)-1*H*-pyrazol-5-amine (**2l**)

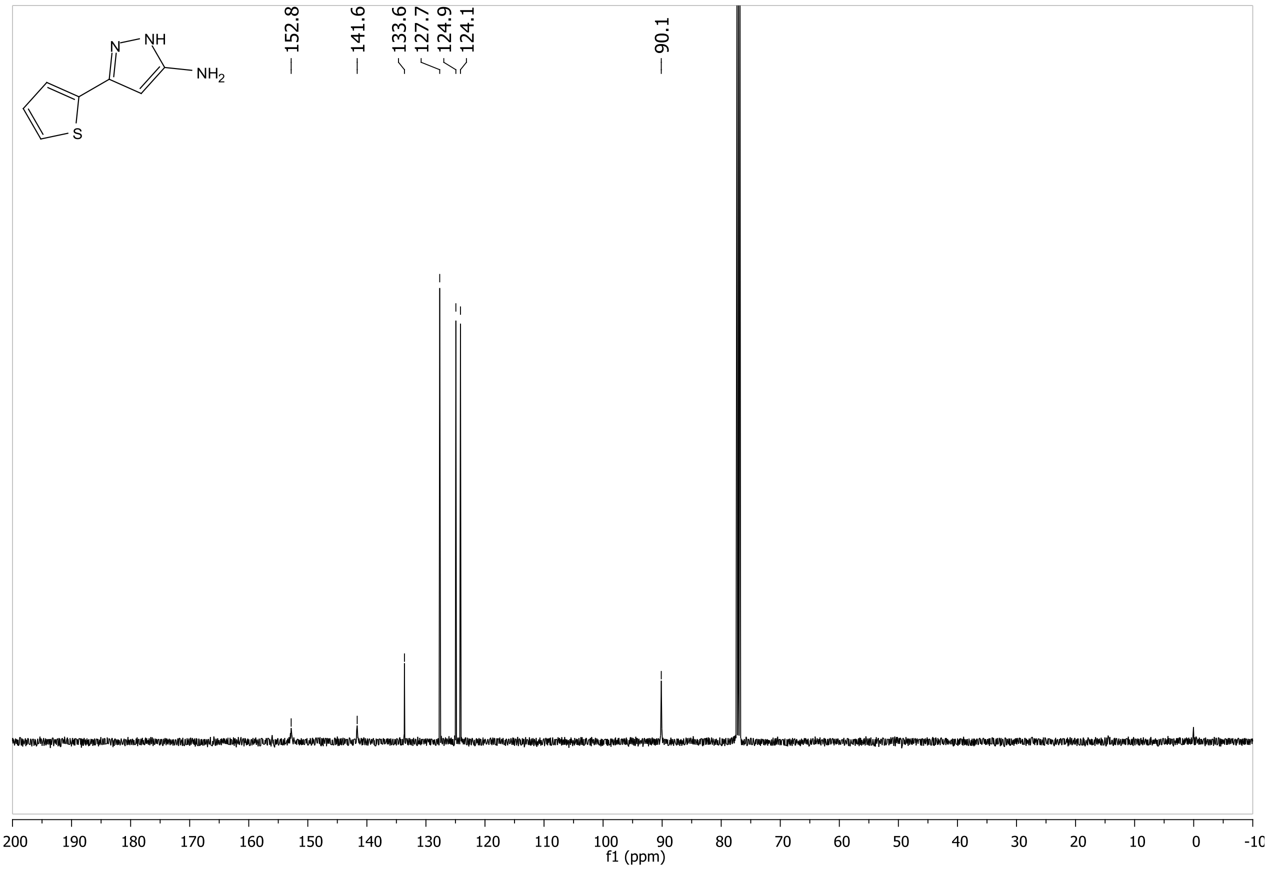

Figure S21. ^{13}C NMR spectrum of 3-(thiophenyl-2-yl)-1*H*-pyrazol-5-amine (2l)

1.2.13. 3-Methyl-1*H*-pyrazol-5-amine (2m)

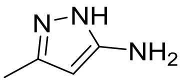

3-Methyl-1*H*-pyrazol-5-amine (**2m**) was prepared as per general procedure and purified by column chromatography methanol in DCM (0-10%). Light brown oil; Yield (0.097 g, 50%); R_f 0.25 (9:1 DCM:MeOH); ^1H NMR (500 MHz, $CDCl_3$) δ 6.14 (bs, 2H, NH_2), 5.36 (s, 1H, CH), 2.13 (s, 3H, CH_3). ^{13}C NMR (126 MHz, $CDCl_3$) δ 153.9 (quaternary), 141.8 (quaternary), 92.0, 11.4. HRMS calcd for CH_8N_3 $[M + H]^+$: 98.0713, found 98.0717. Matches literature data[7].

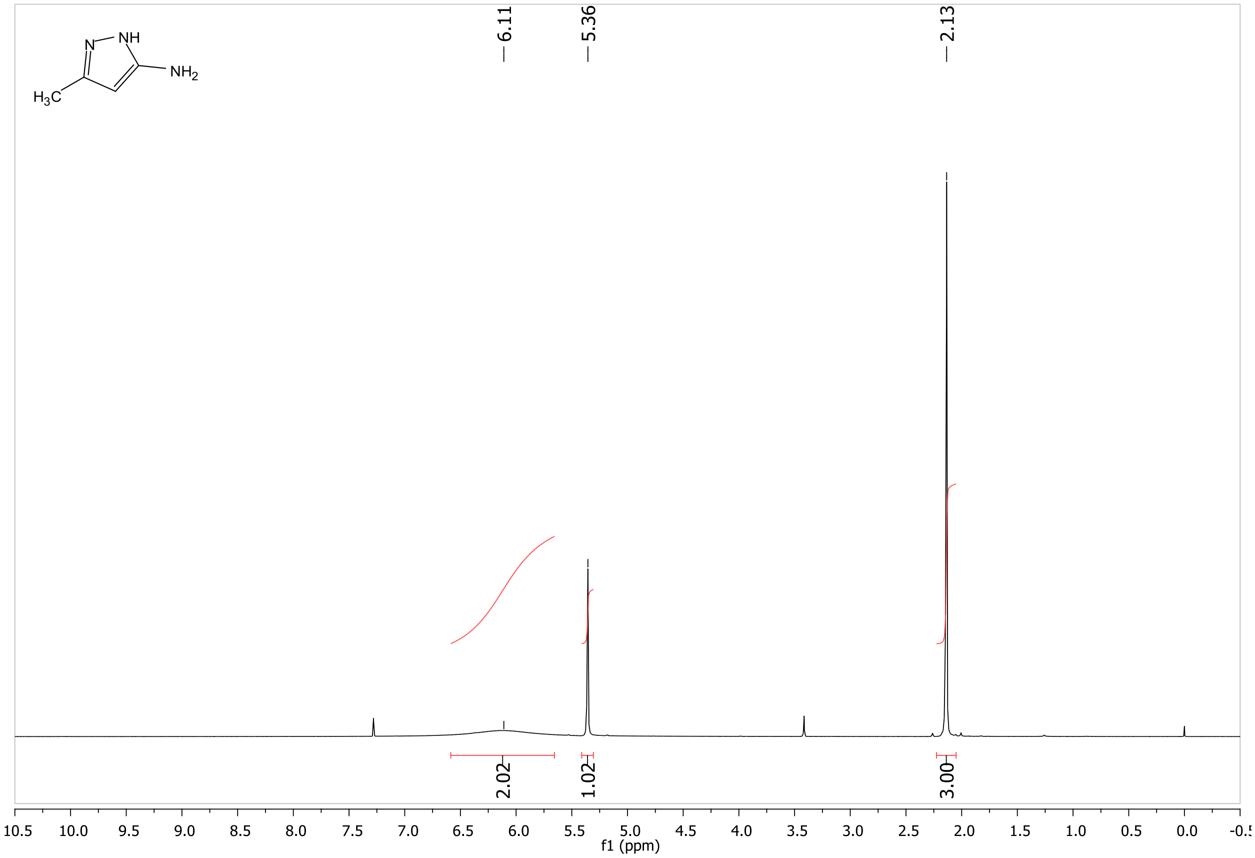

Figure S22. ^1H NMR spectrum of 3-methyl-1*H*-pyrazol-5-amine (**2m**)

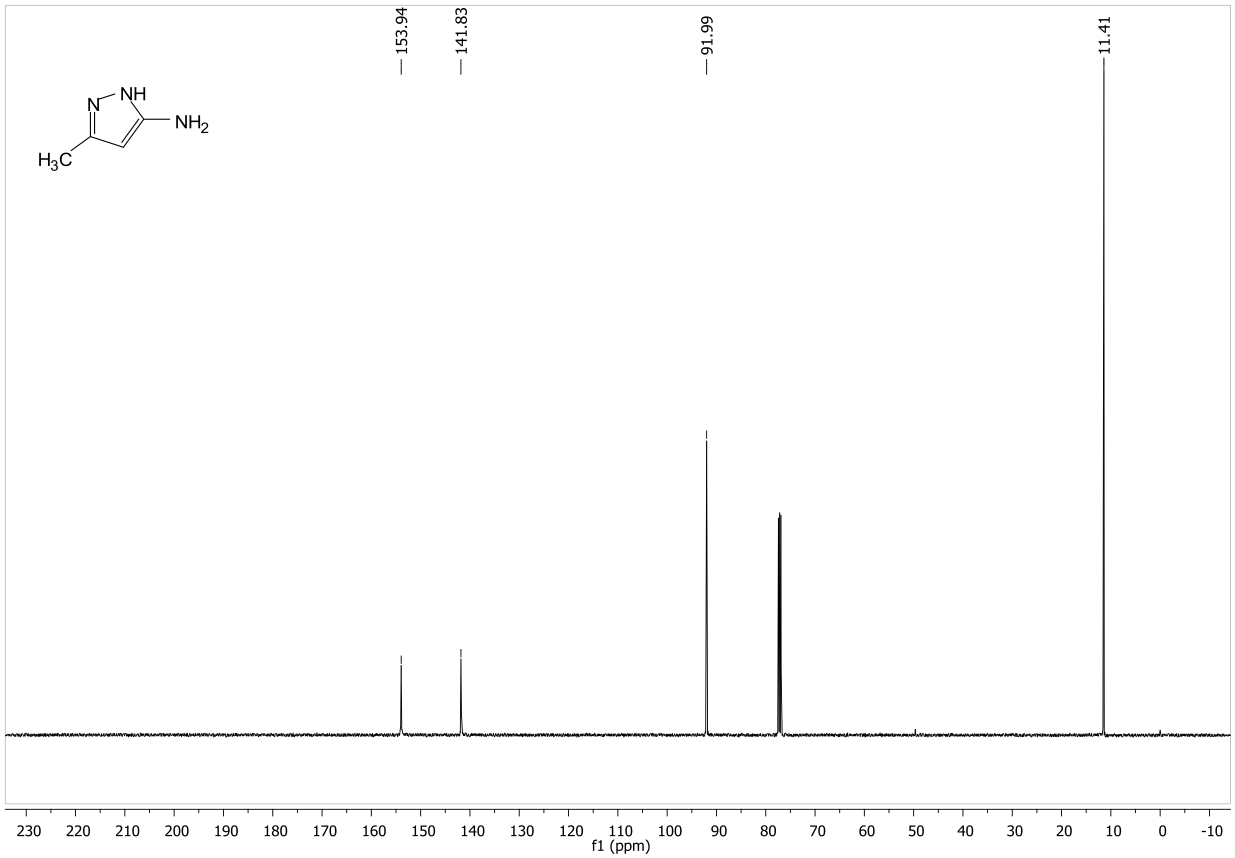

Figure S23. ^{13}C NMR spectrum of 3-methyl-1*H*-pyrazol-5-amine (**2m**)

1.2.14. 3-Ethyl-1*H*-pyrazol-5-amine (2n)

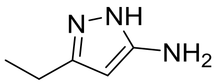

3-Ethyl-1*H*-pyrazol-5-amine (**2n**) was prepared as per general procedure and purified by column chromatography (3:2 EtOAc:Petroleum Ether). Red solid; Yield (0.109 g, 49%); R_f 0.13 (3:2 EtOAc:Petroleum Ether); ^{1}H NMR (500 MHz, $CDCl_3$) δ 5.45 (s, 1H, CH), 2.57 (q, J = 7.5 Hz, 2H, CH_2), 1.23 (t, J = 7.5 Hz, 3H, CH_3). ^{13}C NMR (126 MHz, $CDCl_3$) δ 154.7 (quaternary), 147.3 (quaternary), 91.2, 19.4, 13.1. HRMS calcd for $C_5H_{10}N_3$ $[M + H]^+$: 112.0869, found 112.0874. Matches literature data[8].

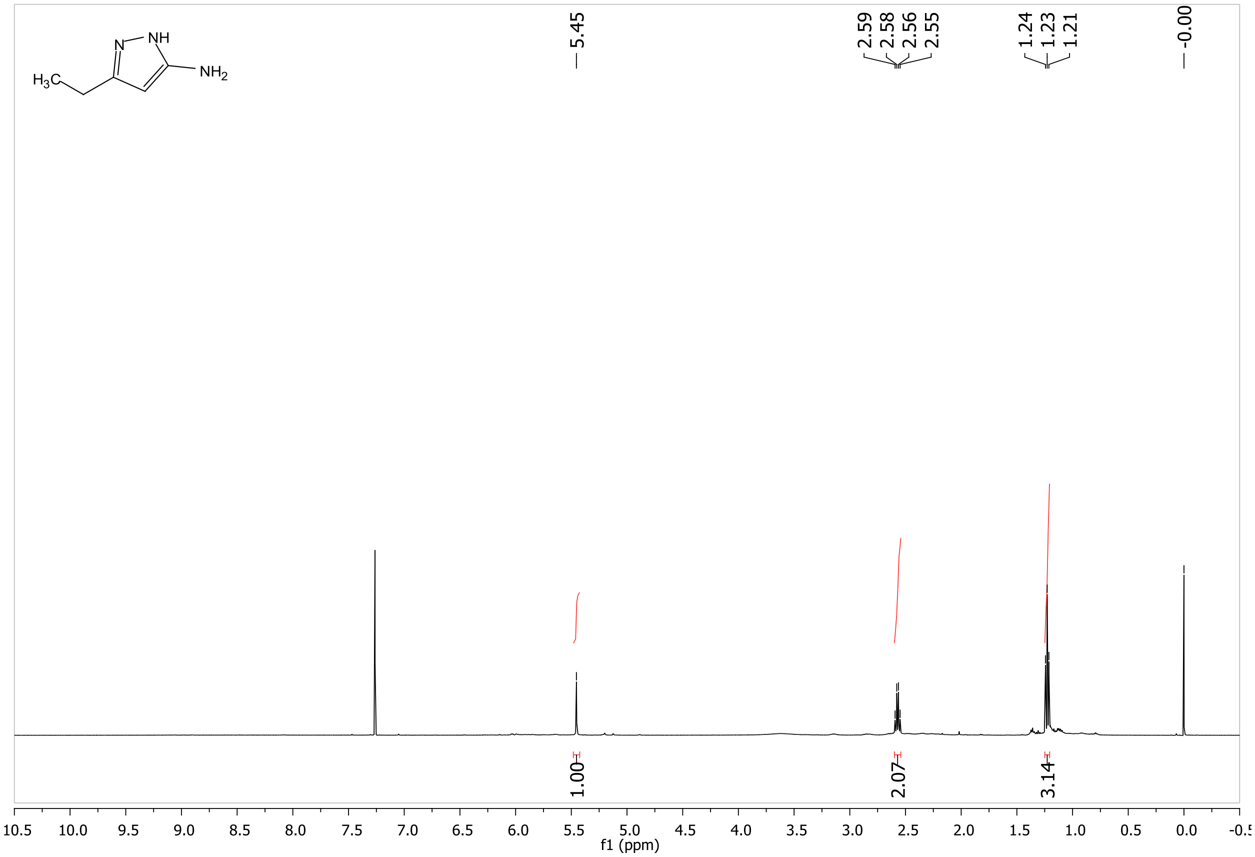

Figure S24. ^{1}H NMR spectrum of 3-ethyl-1*H*-pyrazol-5-amine (**2n**)

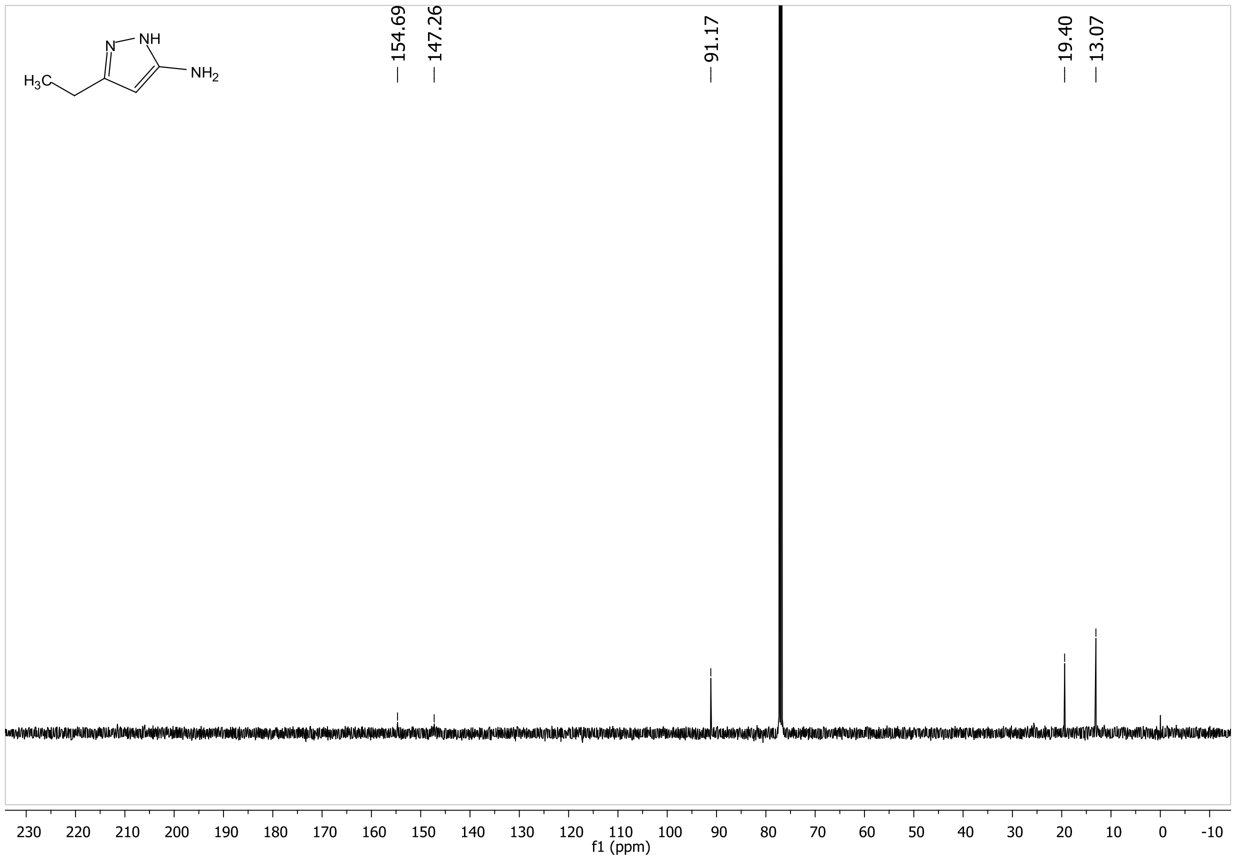

Figure S25. ^{13}C NMR spectrum of 3-ethyl-1*H*-pyrazol-5-amine (2n)

1.2.15. 3-Propyl-1*H*-pyrazol-5-amine (2o)

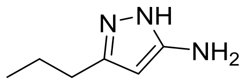

3-Propyl-1*H*-pyrazol-5-amine (**2o**) was prepared as per general procedure and purified by column chromatography (9:1 DCM: MeOH). Yellow oil; Yield (0.091 g, 36%); R_f 0.41 (9:1 DCM:MeOH); ^{1}H NMR (500 MHz, $CDCl_3$) δ 5.45 (s, 1H, CH), 2.51 (t, *J* = 7.5 Hz, 2H, CH_2), 1.76 – 1.43 (m, *J* = 7.5 Hz, 2H, CH_2), 0.96 (t, *J* = 7.5 Hz, 3H, CH_3). ^{13}C NMR (126 MHz, $CDCl_3$) δ 154.7 (quaternary), 145.8 (quaternary), 91.7, 28.2, 22.3, 13.8. HRMS calcd for $CH_{11}N_3$ $[M + H]^+$: 126.1026, found 126.1026. Matches literature data[8].

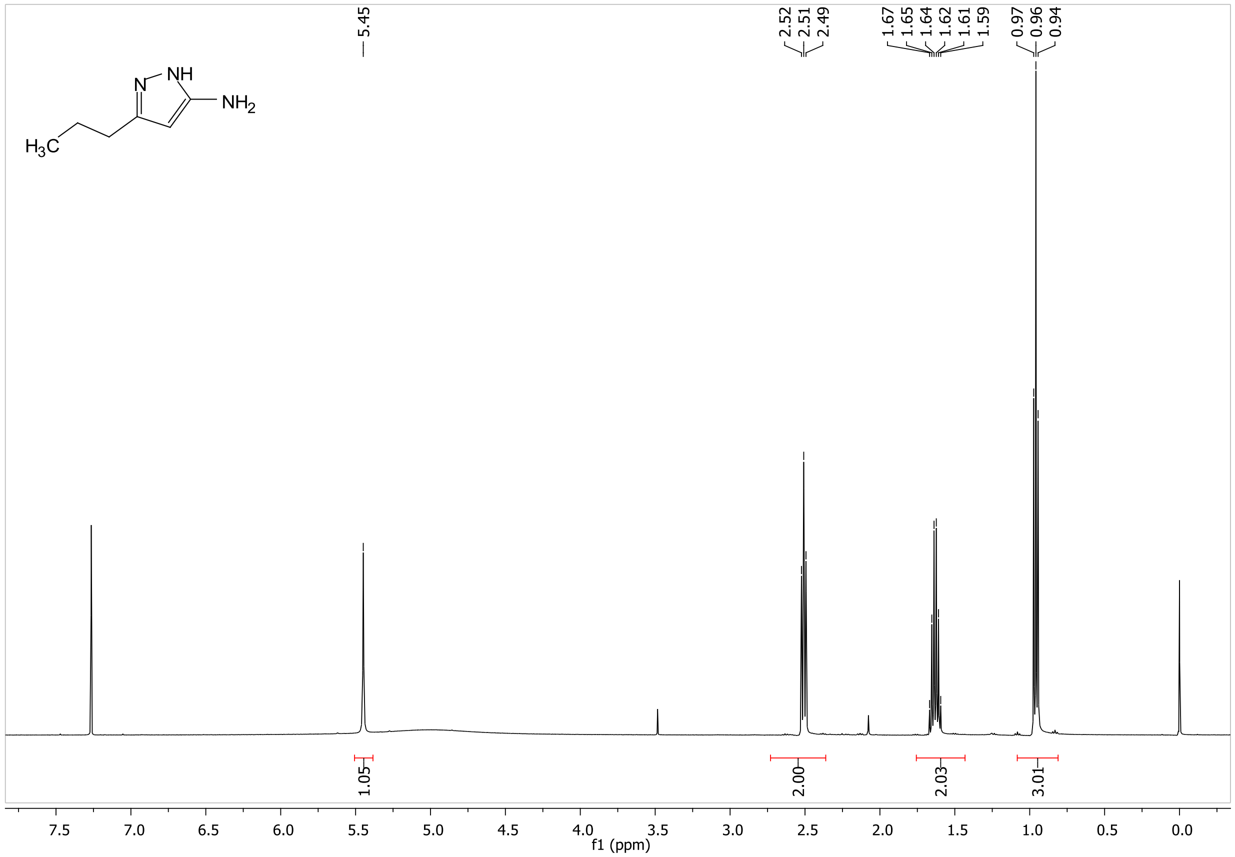

Figure S26. ^{1}H NMR spectrum of 3-propyl-1*H*-pyrazol-5-amine (**2o**)

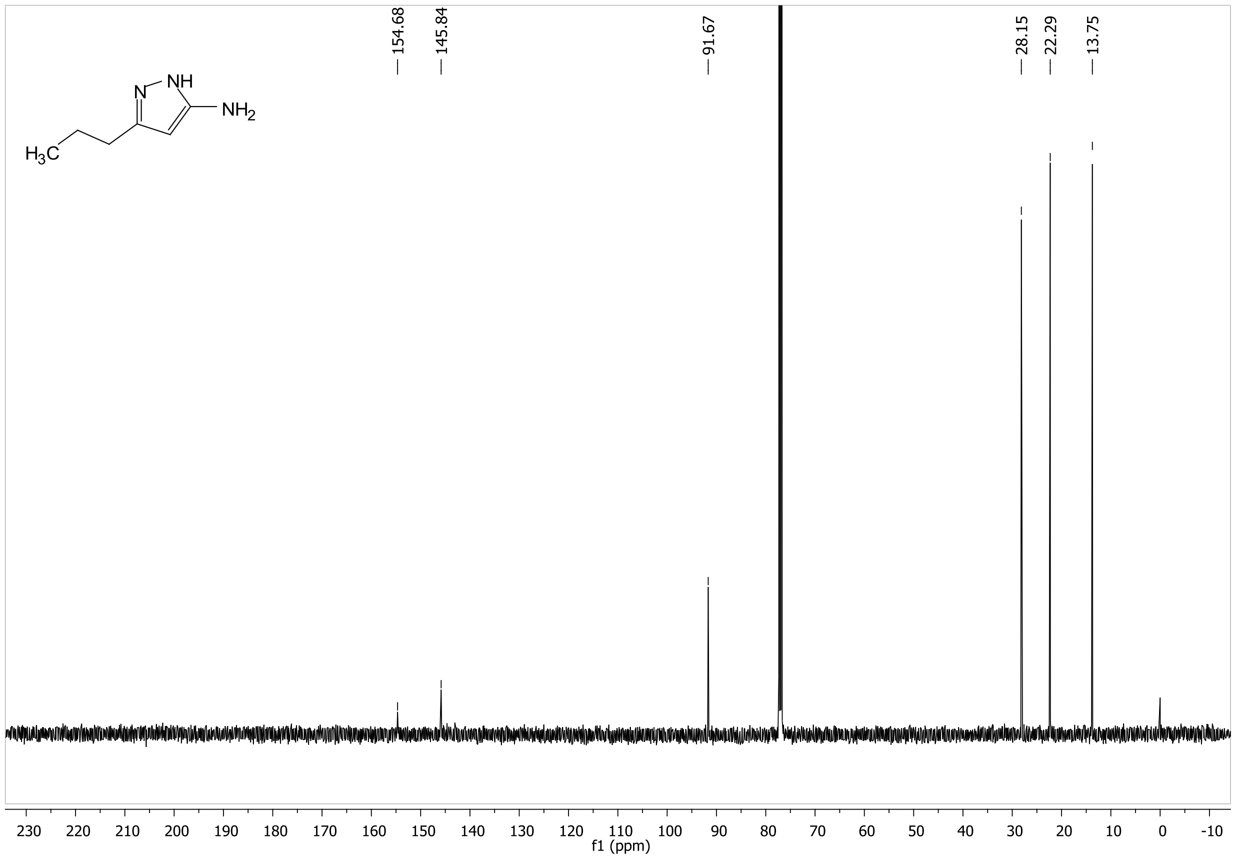

Figure S27. ^{13}C NMR spectrum of 3-propyl-1*H*-pyrazol-5-amine (**2o**)

1.2.16. 3-(*tert*-Butyl)-1*H*-pyrazol-5-amine (2p)

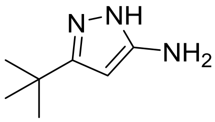

3-(*tert*-Butyl)-1*H*-pyrazol-5-amine (**2p**) was prepared as per general procedure and purified by column chromatography (3:2 EtOAc:Petroleum Ether). Red solid; Yield (0.216 g, 77%); R_f 0.12 (3:2 EtOAc:Petroleum Ether); ^1H NMR (500 MHz, $CDCl_3$) δ 5.42 (s, 1H, CH), 1.26 (s, 9H, 3 x CH_3). ^{13}C NMR (126 MHz, $CDCl_3$) δ 155.2 (quaternary), 154.1 (quaternary), 89.3, 31.0 (quaternary), 30.0. HRMS calcd for $C_7H_{14}N_3$ $[M + H]^+$: 140.1182, found 140.1185. Matches literature data[7].

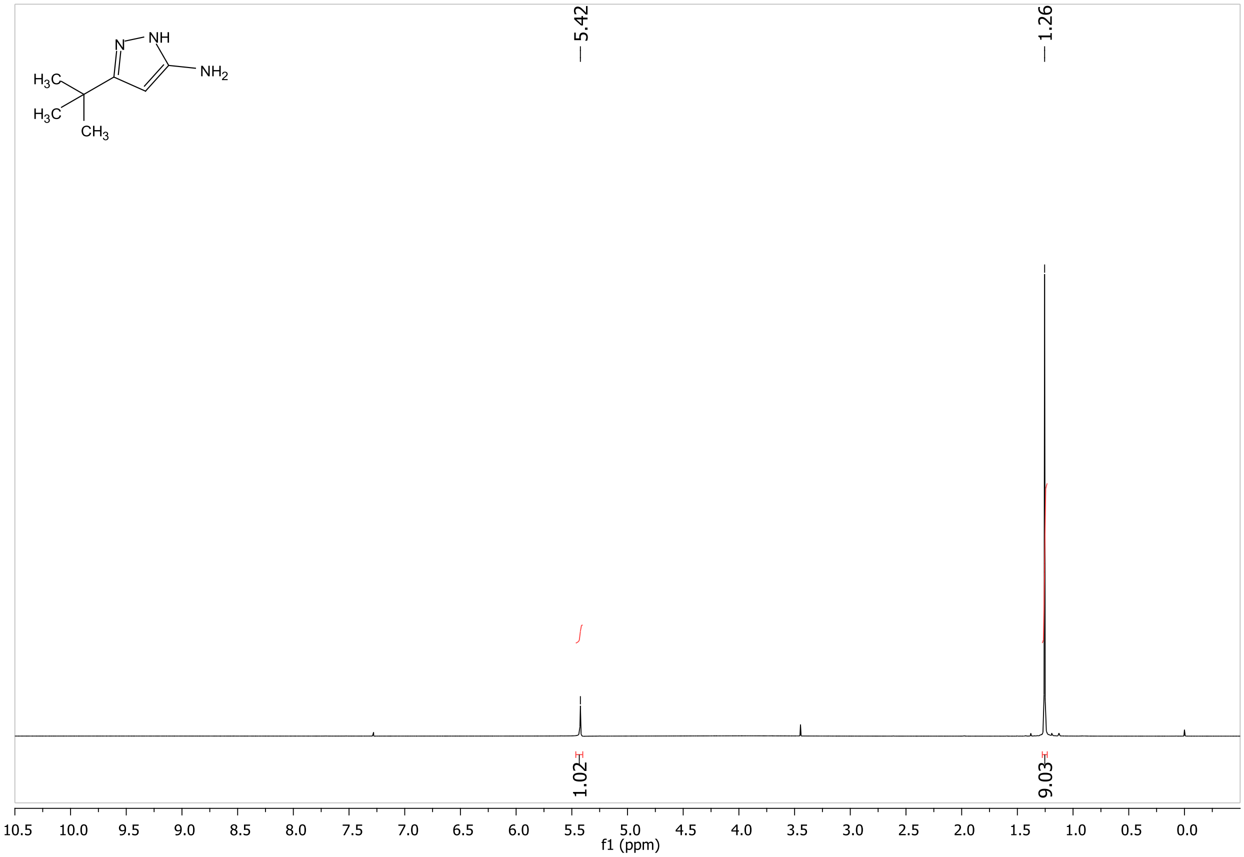

Figure S28. ^1H NMR spectrum of 3-(*tert*-butyl)-1*H*-pyrazol-5-amine (**2p**)

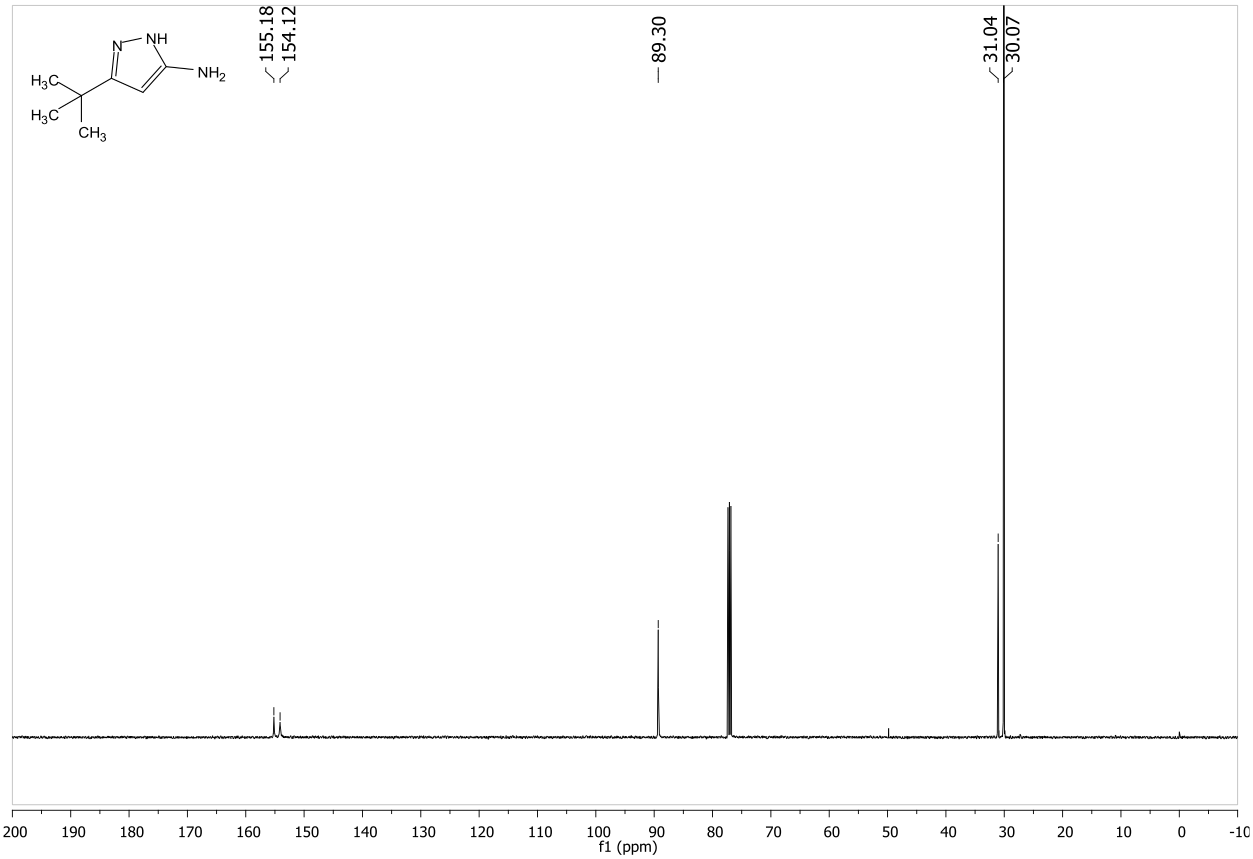

Figure S29. ^{13}**C NMR spectrum of 3-(*tert*-butyl)-1*H*-pyrazol-5-amine (2p)**

1.3. General procedure of one pot synthesis of pyrazolopyrimidones

A microwave tube was charged with ketonitrile (0.9 mmol), methanol (1 mL), and hydrazine monohydrate (1.2 mmol) and subjected to microwave irradiation (100 W, 150 °C) for 5 minutes. Subsequently, to this solution was added ketoester (0.9 mmol) and acetic acid (0.5 mmol), and the mixture was subjected to microwave irradiation (100 W, 150 °C) for 2 hours. Volatiles were removed under reduced pressure. The mixture was purified by either trituration with cold methanol or ethyl acetate, or by using column chromatography.

1.3.1. 2,5-Diphenylpyrazolo[1,5-*a*]pyrimidin-7(4*H*)-one (3a)

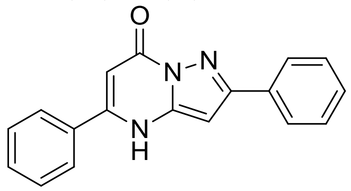

Microwave one pot: 2,5-diphenylpyrazolo[1,5-*a*]pyrimidin-7(4*H*)-one (**3a**) was prepared as per general procedure and purified by trituration with cold MeOH. White solid; Yield (0.135 g, 52%); ^{1}H NMR (300 MHz, DMSO) δ 12.61 (bs, 1H, NH), 8.01 (m, 2H, Ar), 7.87 (m, 2H, Ar), 7.60 (m, 3H, Ar), 7.46 (m, 3H, Ar), 6.67 (s, 1H, CH), 6.10 (s, 1H, CHCO). ^{13}C NMR (75 MHz, DMSO) δ 156.2 (quaternary), 153.3 (quaternary), 149.8 (quaternary), 143.2 (quaternary), 132.4 (quaternary), 132.3 (quaternary), 131.1, 129.0, 128.9, 128.7, 127.2, 126.2, 94.0, 86.6. HRMS calcd for $C_{18}H_{14}N_3O_2$ [M + H]$^+$: 288.1131, found 288.1133. IR (KBr) 3033, 1669 (C=O), 1611, 1448, 768, 692, 548. Matches literature data[9].

Reflux from amino pyrazole, 18 hours: 2,5-diphenylpyrazolo[1,5-a]pyrimidin-7(4*H*)-one (**3a**) was prepared by heating 3-phenyl-1*H*-pyrazol-5-amine (0.143 g, 0.9 mmol), ethyl benzoylacetate (0.173 g, 0.9 mmol), AcOH (0.027 g, 0.5 mmol), and MeOH (2 mL) in a round-bottom flask at reflux for 18 hours. Volatiles were subsequently removed under reduced pressure. The resulting residue was subjected to column chromatography (1:1 EtOAc:Petroleum Ether) to give crude product that was further purified by trituration with EtOAc. The remaining residue, which contained the product, was dissolved in DCM (15 mL) and washed with aqueous $NaHCO_3$ (conc, 15 mL). The resulting organic layer was separated and the solvent removed under reduced pressure to give the final product. Yield (0.064 g, 25%).

Reflux from amino pyrazole, 2 hours: 2,5-diphenylpyrazolo[1,5-*a*]pyrimidin-7(4*H*)-one (**3a**) was prepared by heating 3-phenyl-1*H*-pyrazol-5-amine (0.143 g, 0.9 mmol), ethyl benzoylacetate (0.173 g, 0.9 mmol), AcOH (0.027 g, 0.5 mmol), and MeOH (2 mL) in a round-bottom flask at reflux for 2 hours. Volatiles were subsequently removed under reduced pressure. The resulting residue was subjected to column chromatography (1:1 EtOAc:Petroleum Ether) to give crude product that was further purified by trituration with EtOAc. The remaining residue, which contained the product, was dissolved in DCM (15 mL) and washed with aqueous $NaHCO_3$ (conc, 15 mL). The resulting organic layer was separated and the solvent removed under reduced pressure to give the final product. Yield (0.027 g, 11%).

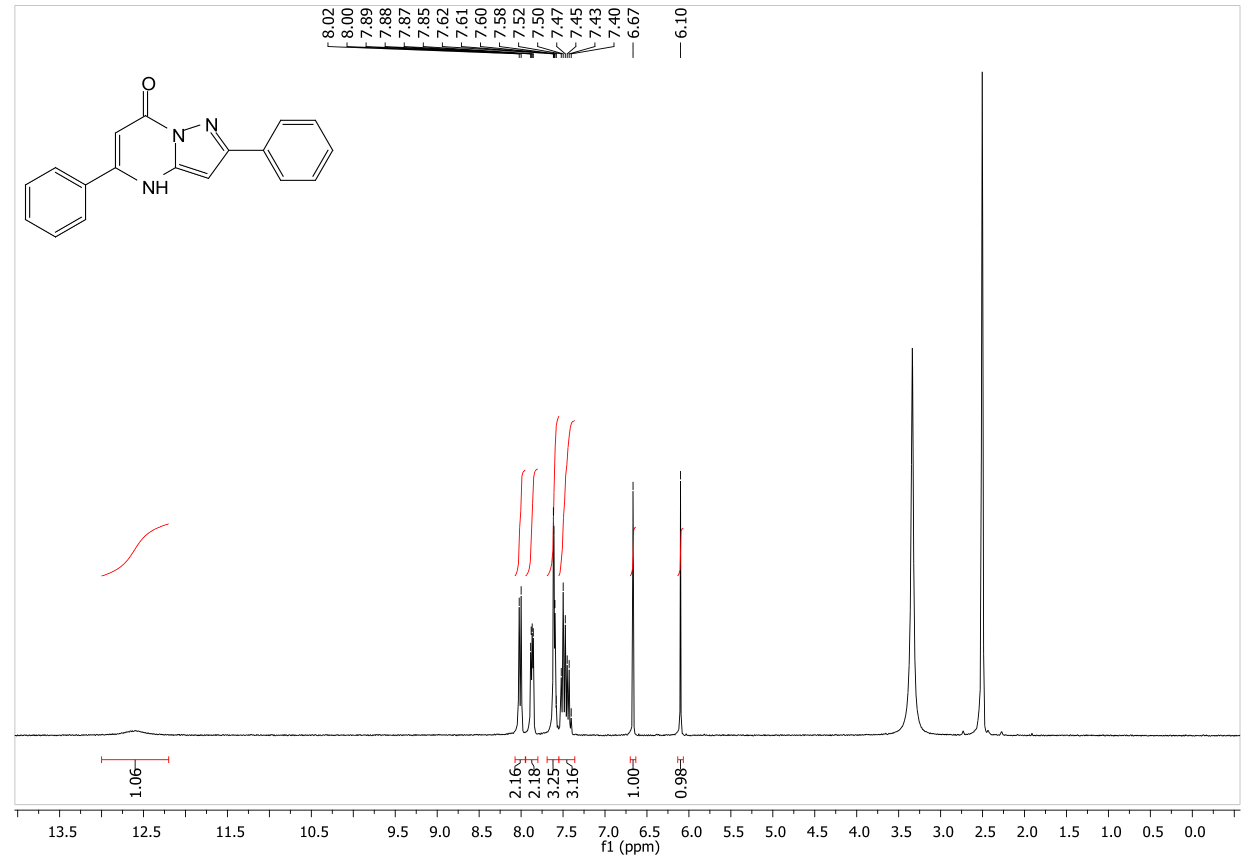

Figure S30. ^{1}H NMR spectrum of 2,5-diphenylpyrazolo[1,5-*a*]pyrimidin-7(4*H*)-one (**3a**)

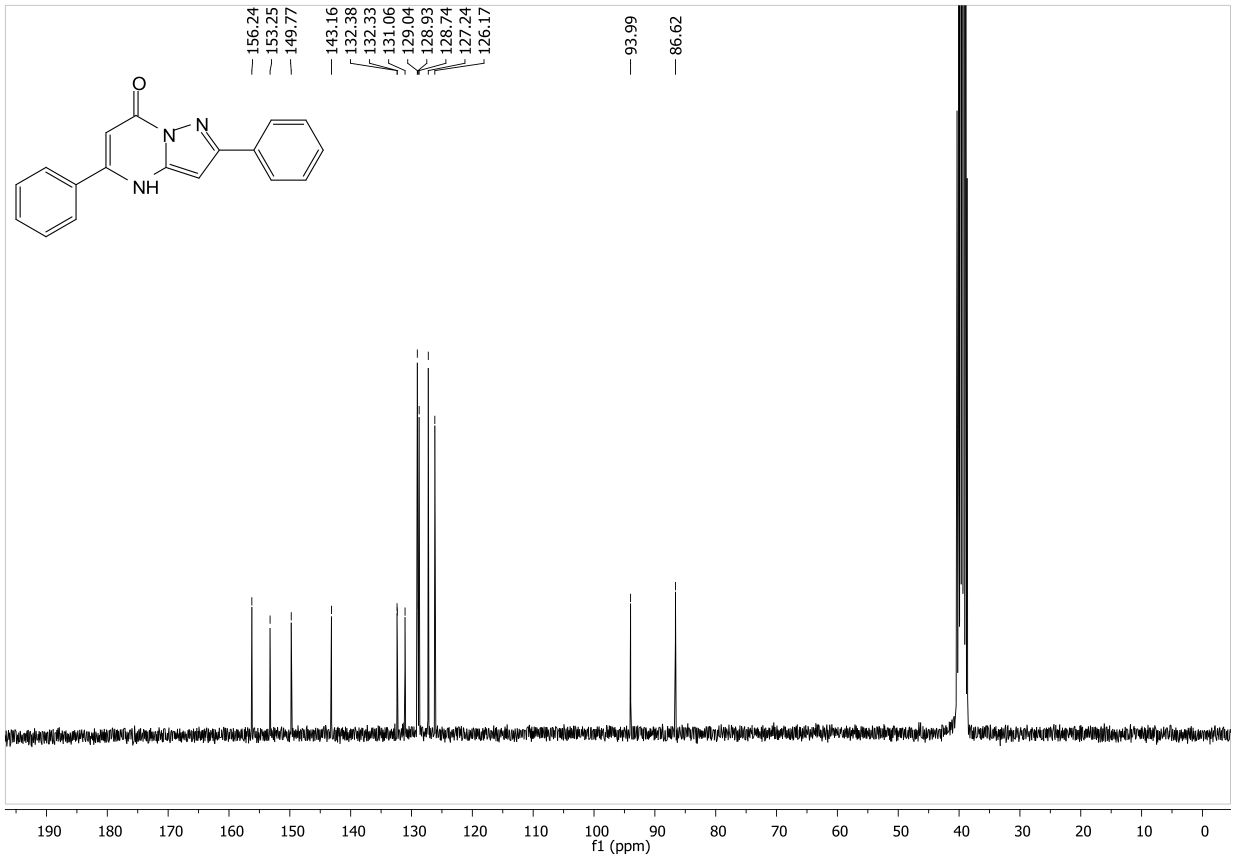

Figure S31. ^{13}C NMR spectrum of 2,5-diphenylpyrazolo[1,5-*a*]pyrimidin-7(4*H*)-one (**3a**)

1.3.2. **5-Methyl-2-phenylpyrazolo[1,5-*a*]pyrimidin-7(4*H*)-one (3b)**

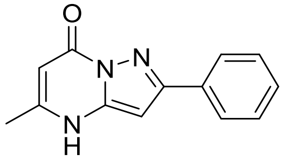

5-Methyl-2-phenylpyrazolo[1,5-*a*]pyrimidin-7(4*H*)-one (**3b**) was prepared as per general procedure and purified by trituration with cold MeOH. Light yellow solid; Yield (0.122g, 55%); ^{1}H NMR (300 MHz, DMSO) δ 7.97 (d, *J* = 7.0 Hz, 2H, Ar), 7.44 (m, 3H, Ar), 6.57 (s, 1H CH), 5.60 (s, 1H, CHCO), 2.30 (s, 3H, CH_3). ^{13}C NMR (75 MHz, DMSO) δ 156.1 (quaternary), 152.8 (quaternary), 150.2 (quaternary), 142.8 (quaternary), 132.5 (quaternary), 128.8, 128.7, 126.1, 95.2, 85.4, 18.6. HRMS calcd for $C_{13}H_{12}N_3O$ $[M + H]^+$: 226.0975, found 226.0977. IR (KBr) 2895, 1667 (C=O), 1623, 1420, 753, 541. Matches literature data[10].

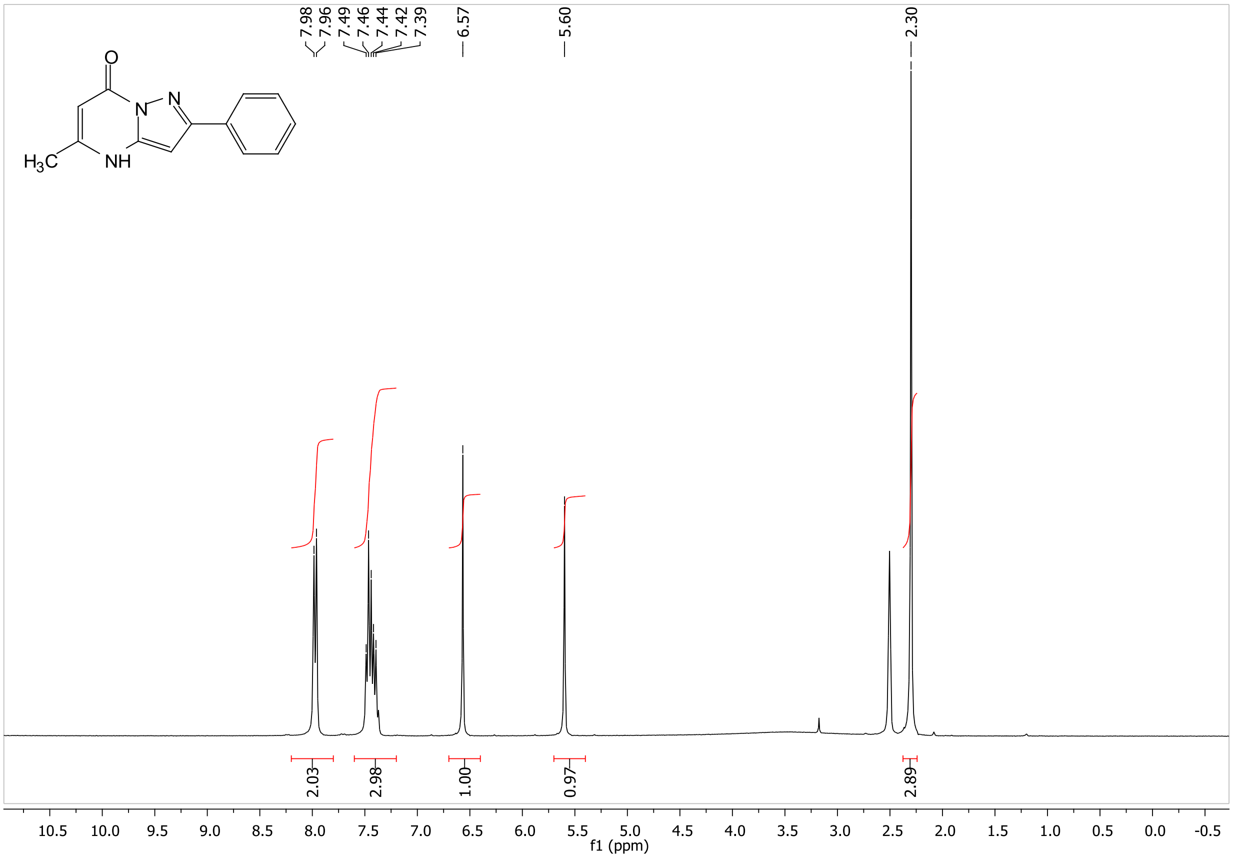

Figure S32. ^{1}H NMR spectrum of 5-methyl-2-phenylpyrazolo[1,5-*a*]pyrimidin-7(4*H*)-one (**3b**)

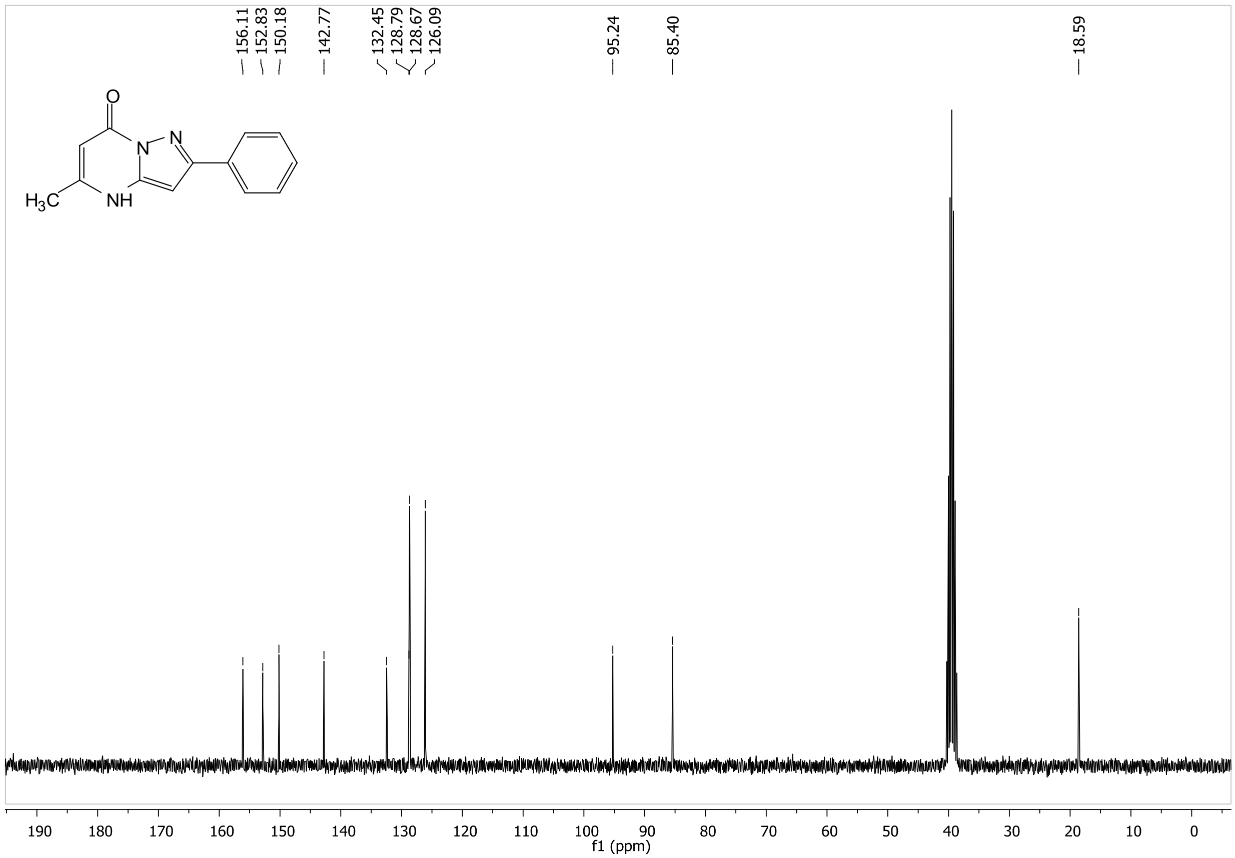

Figure S33. ^{13}C NMR spectrum of 5-methyl-2-phenylpyrazolo[1,5-*a*]pyrimidin-7(4*H*)-one (**3b**)

1.3.3. 5-(4-Nitrophenyl)-2-phenylpyrazolo[1,5-*a*]pyrimidin-7(4*H*)-one (3c)

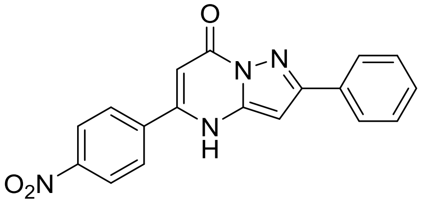

5-(4-Nitrophenyl)-2-phenylpyrazolo[1,5-*a*]pyrimidin-7(4*H*)-one (**3c**) was prepared as per general procedure and purified by trituration with cold MeOH. Yellow solid; Yield (0.087 g, 30%); ^{1}H NMR (300 MHz, DMSO) δ 8.40 (d, *J* = 8.8 Hz, 2H, Ar), 8.14 (d, *J* = 8.8 Hz, 2H, Ar), 8.01 (d, *J* = 6.9 Hz, 2H, Ar), 7.57 – 7.36 (m, 3H, Ar), 6.67 (s, 1H, CH), 6.19 (s, 1H, CHCO).^{13}C NMR (75 MHz, DMSO) δ 155.9 (quaternary), 153.6 (quaternary), 148.9 (quaternary), 147.7 (quaternary), 143.3 (quaternary), 138.6 (quaternary), 132.4 (quaternary), 128.9, 128.8, 128.7, 126.3, 123.9, 95.6, 86.9. HRMS calcd for $C_{18}H_{13}N_4O_3$ [M + H]$^+$: 333.0982, found 333.0993. IR (KBr) 3122, 3032, 1667 (C=O), 1615, 774.

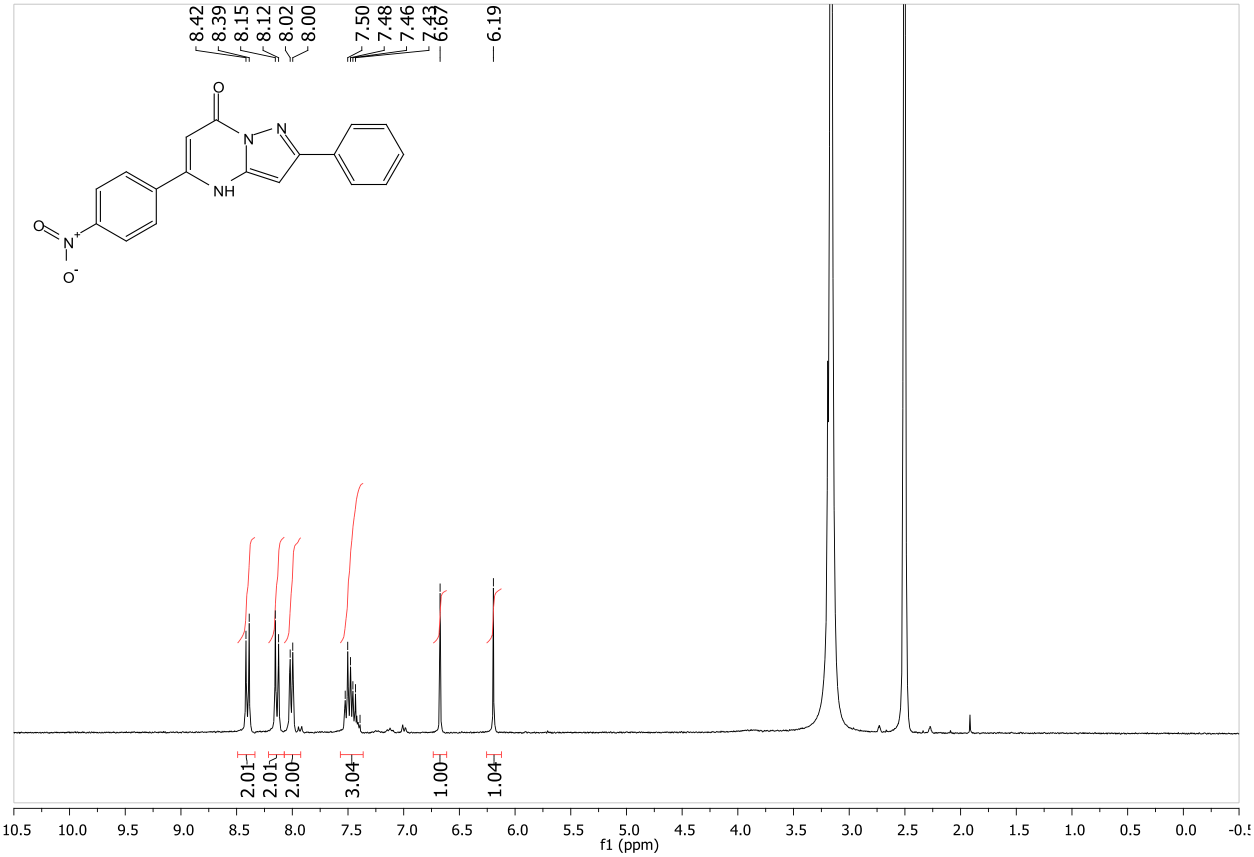

Figure S34. ^{1}H NMR spectrum of 5-(4-nitrophenyl)-2-phenylpyrazolo[1,5-*a*]pyrimidin-7(4*H*)-one (**3c**)

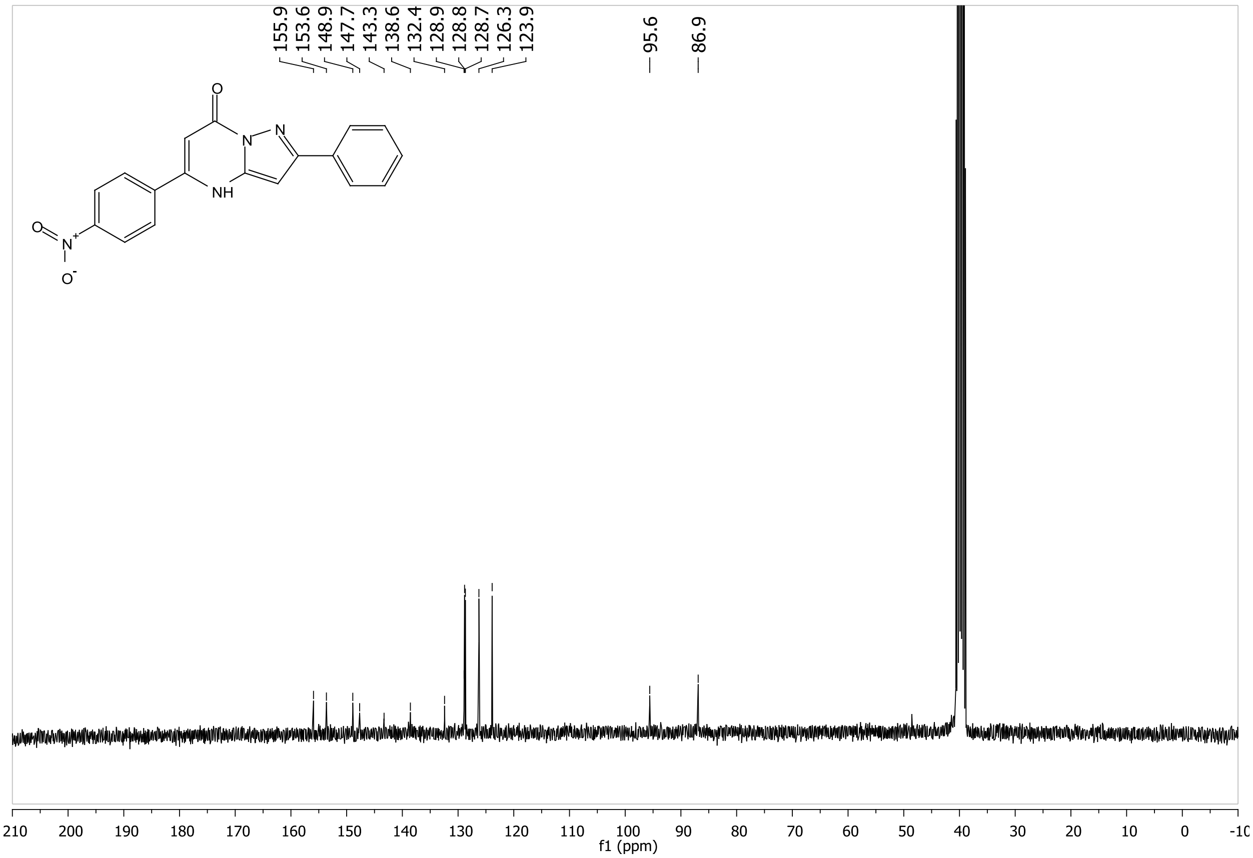

Figure S35. ^{13}C NMR spectrum of 5-(4-nitrophenyl)-2-phenylpyrazolo[1,5-*a*]pyrimidin-7(4*H*)-one (**3c**)

1.3.4. 5-(4-Methoxyphenyl)-2-phenylpyrazolo[1,5-*a*]pyrimidin-7(4*H*)-one (3d)

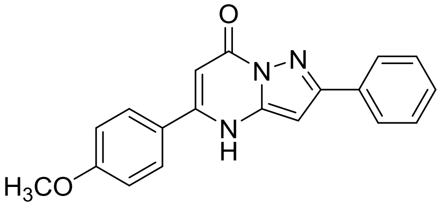

5-(4-Methoxyphenyl)-2-phenylpyrazolo[1,5-*a*]pyrimidin-7(4*H*)-one (**3d**) was prepared as per general procedure and purified by column chromatography (5:5 EtOAc:Petroluem Ether). White solid; Yield (0.047 g, 16%); Rf: 0.59 (5:5 EtOAc:Petroluem Ether); ^{1}H NMR (300 MHz, DMSO) δ 8.00 (m, 2H, Ar), 7.85 (d, *J* = 8.8 Hz, 2H, Ar), 7.47 (m, 3H, Ar), 7.14 (d, *J* = 8.8 Hz, 2H, Ar), 6.63 (s, 1H, CH), 6.05 (s, 1H, CHCO), 3.86 (s, 3H, OCH_3). ^{13}C NMR (75 MHz, DMSO) δ 161.5 (quaternary), 156.3 (quaternary), 153.0 (quaternary), 149.6 (quaternary), 132.5 (quaternary), 130.7 (quaternary), 128.9, 128.8, 128.7, 126.1, 124.4 (quaternary), 114.4, 92.8, 86.5, 55.5 (OCH_3). HRMS calcd for $C_{19}H_{16}N_3O_2$ [M + H]$^+$: 318.1237, found 318.1233. IR (KBr) 3062, 1665 (C=O), 1607, 1248, 770, 543.

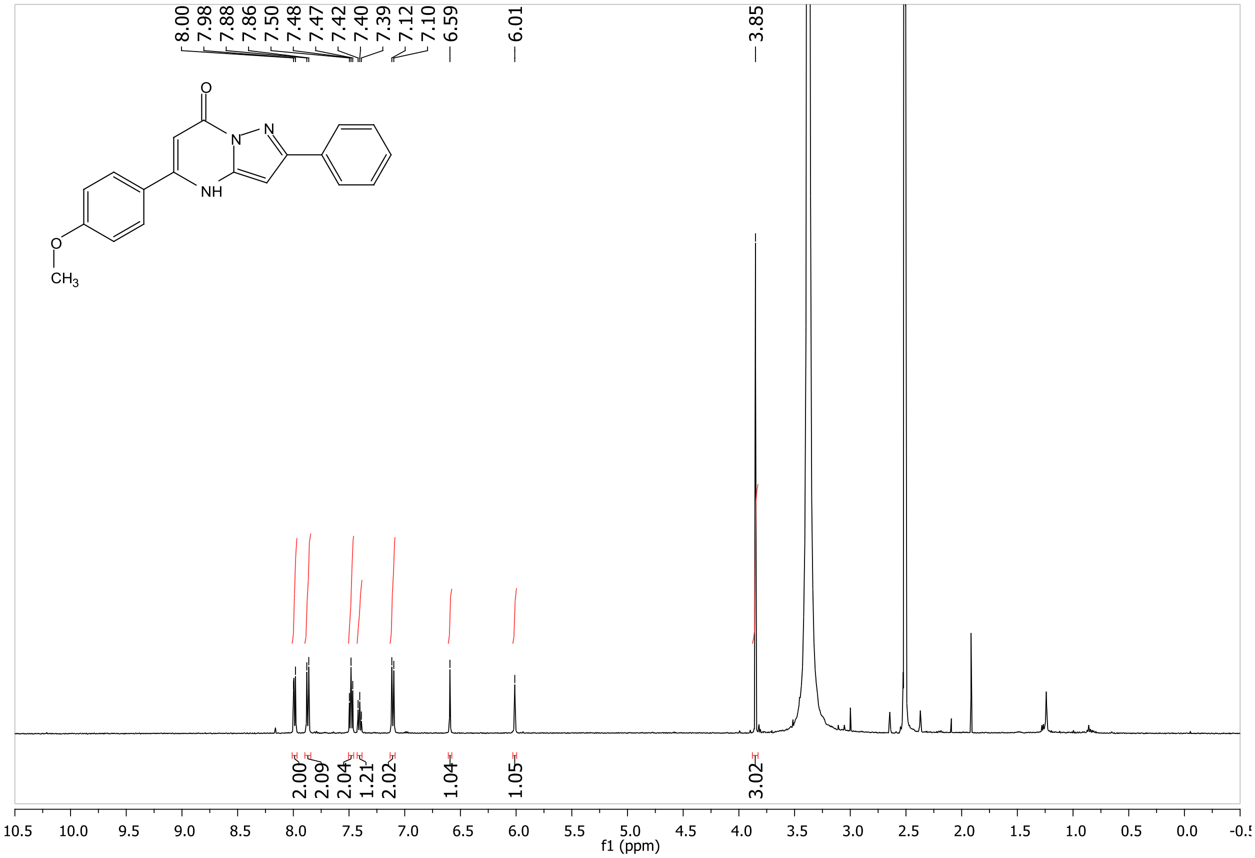

Figure S36. ^{1}H NMR spectrum of 5-(4-methoxyphenyl)-2-phenylpyrazolo[1,5-*a*]pyrimidin-7(4*H*)-one (**3d**)

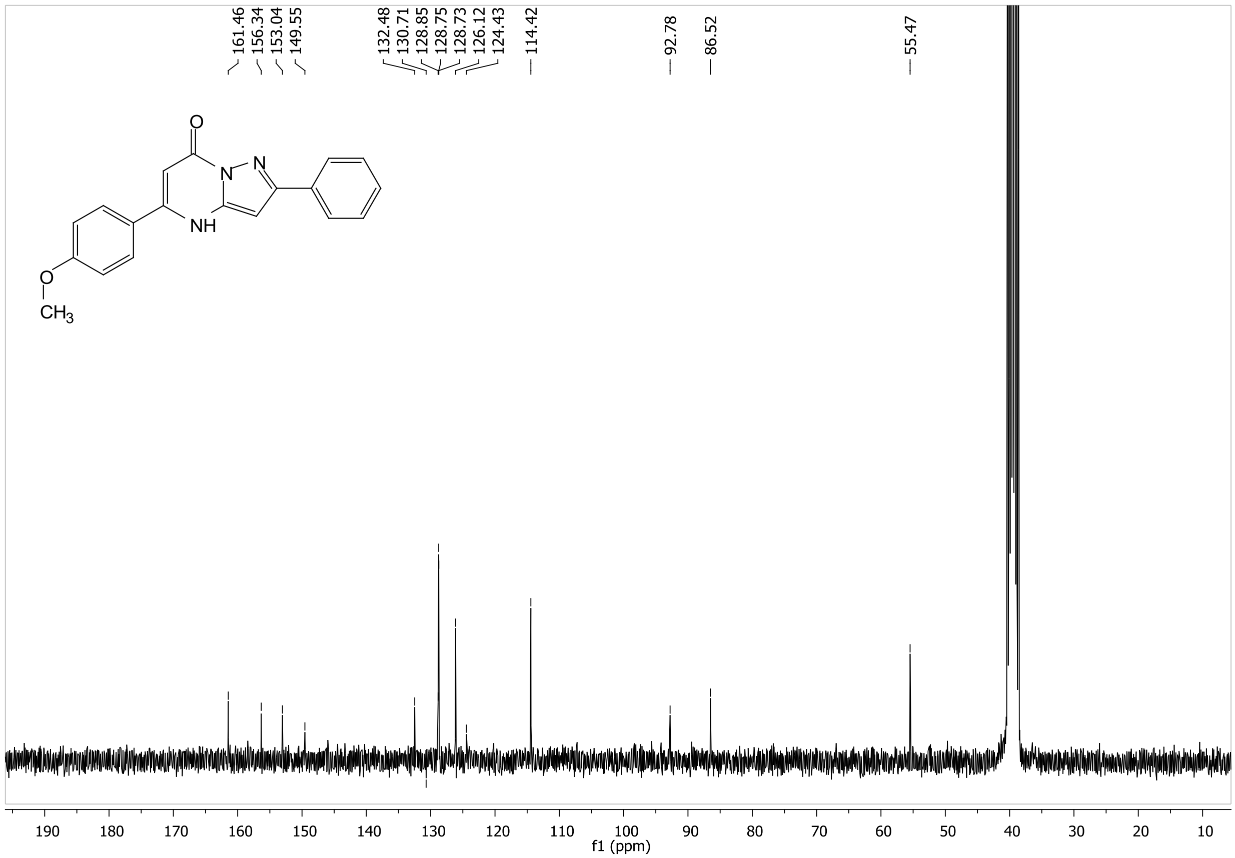

Figure S37. ^{13}C NMR spectrum of 5-(4-methoxyphenyl)-2-phenylpyrazolo[1,5-*a*]pyrimidin-7(4*H*)-one (**3d**)

1.3.5. 2-Phenyl-5-(2,3,4,5-tetrafluorophenyl)pyrazolo[1,5-*a*]pyrimidin-7(4*H*)-one (3e)

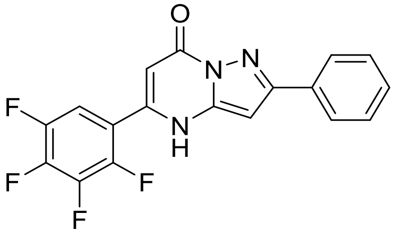

2-Phenyl-5-(2,3,4,5-tetrafluorophenyl)pyrazolo[1,5-*a*]pyrimidin-7(4*H*)-one (**3e**) was prepared as per general procedure and purified by trituration with cold MeOH. Yellow solid; Yield (0.071 g, 22%); ^{1}H NMR (500 MHz, DMSO) δ 8.05 – 8.01 (m, 2H, Ar), 7.94 – 7.87 (m, 1H, Ar), 7.53 – 7.48 (m, 2H, Ar), 7.46 – 7.42 (m, 1H, Ar), 6.74 (s, 1H, CH), 6.01 (d, J_{HF} = 0.8 Hz, 1H, CHCO). ^{13}C NMR (126 MHz, DMSO) δ 156.2 (quaternary), 154.1 (quaternary), 143.5 (quaternary), 142.8 (quaternary), 132.7 (quaternary), 129.6, 129.2, 126.8, 113.3 (d, J_{CF} = 19.3 Hz), 97.9, 87.4. HRMS calcd for $C_{18}H_{10}F_4N_3O$ [M + H]$^+$: 360.0755, found 360.0745. IR (KBr) 3124, 3066, 1664 (C=O), 1608, 769.

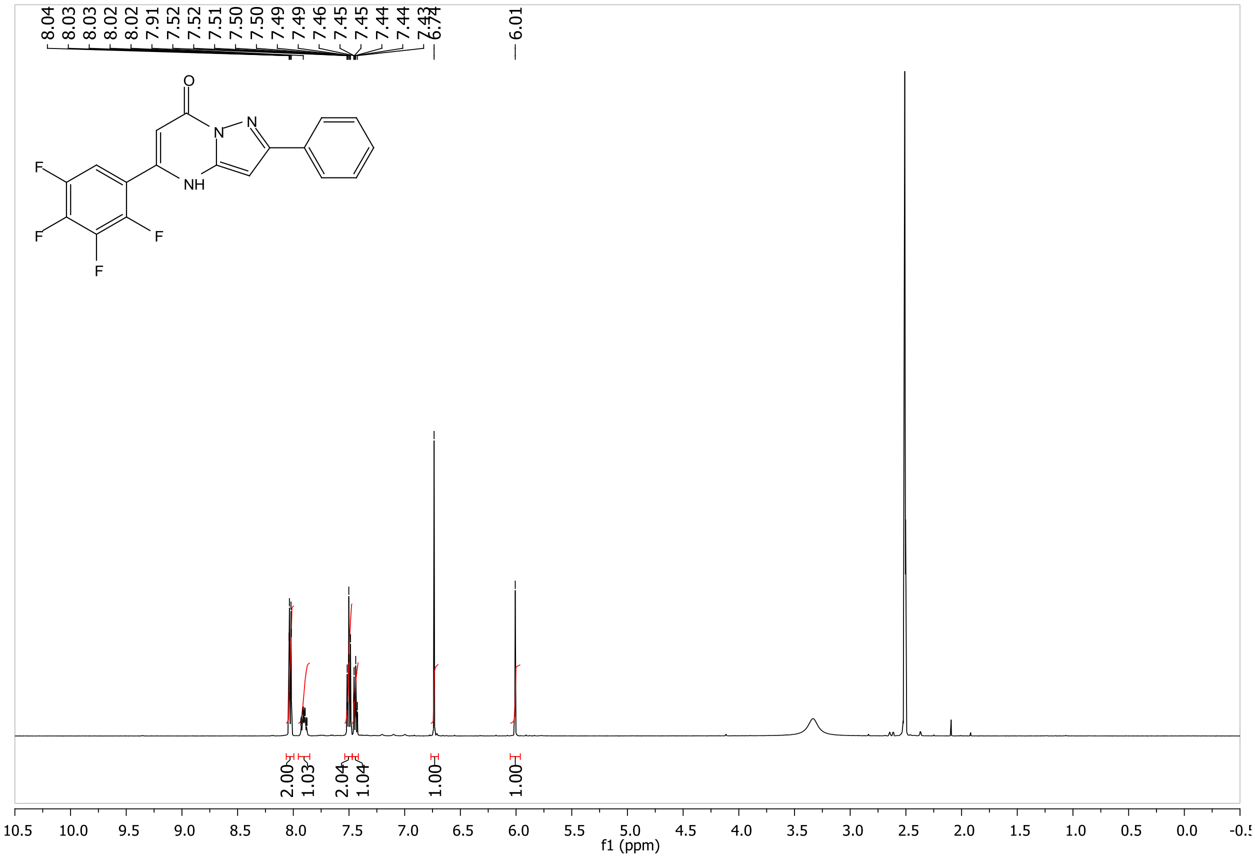

Figure S38. ^{1}H NMR spectrum of 2-phenyl-5-(2,3,4,5-tetrafluorophenyl)pyrazolo[1,5-*a*]pyrimidin-7(4*H*)-one (**3e**)

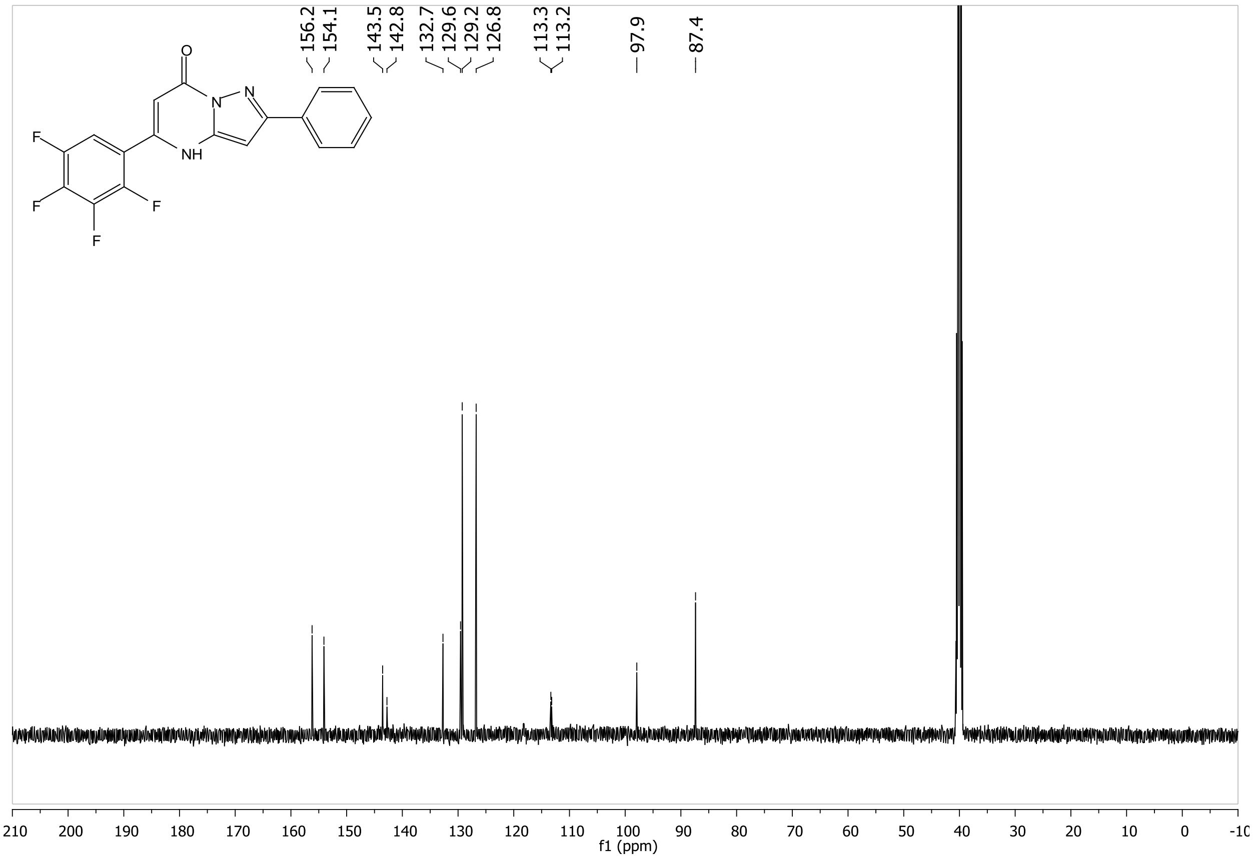

Figure S39. ^{13}C NMR spectrum of 2-phenyl-5-(2,3,4,5-tetrafluorophenyl)pyrazolo[1,5-*a*]pyrimidin-7(4*H*)-one (**3e**)

1.3.6. 2-(3-Chlorophenyl)-5-phenylpyrazolo[1,5-*a*]pyrimidin-7(4*H*)-one (3f)

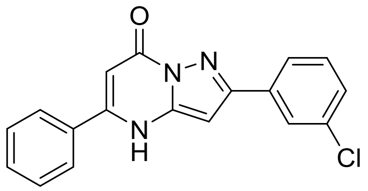

2-(3-Chlorophenyl)-5-phenylpyrazolo[1,5-*a*]pyrimidin-7(4*H*)-one (**3f**) was prepared as per general procedure and purified by trituration with cold MeOH. Green solid; Yield (0.131 g, 45%); ^{1}H NMR (500 MHz, DMSO) δ 12.69 (bs, 1H, NH), 8.07 (s, 1H, Ar), 8.00 (d, *J* = 7.5 Hz, 1H, Ar), 7.91 – 7.84 (m, 2H, Ar), 7.67 – 7.56 (m, 3H, Ar), 7.56 – 7.46 (m, 2H, Ar), 6.76 (s, 1H, CH), 6.12 (s, 1H, CHCO). ^{13}C NMR (126 MHz, DMSO) δ 158.9 (quaternary), 156.8 (quaternary), 152.3 (quaternary), 150.7 (quaternary), 135.0 (quaternary), 134.1 (quaternary), 131.6, 131.2, 129.6, 129.2, 127.8, 126.2, 125.3, 94.5, 87.7. HRMS calcd for $C_{18}H_{13}ClN_3O$ $[M + H]^+$: 322.0742, found 322.0744. IR (KBr) 3062, 1662 (C=O), 1607, 1321, 772, 541.

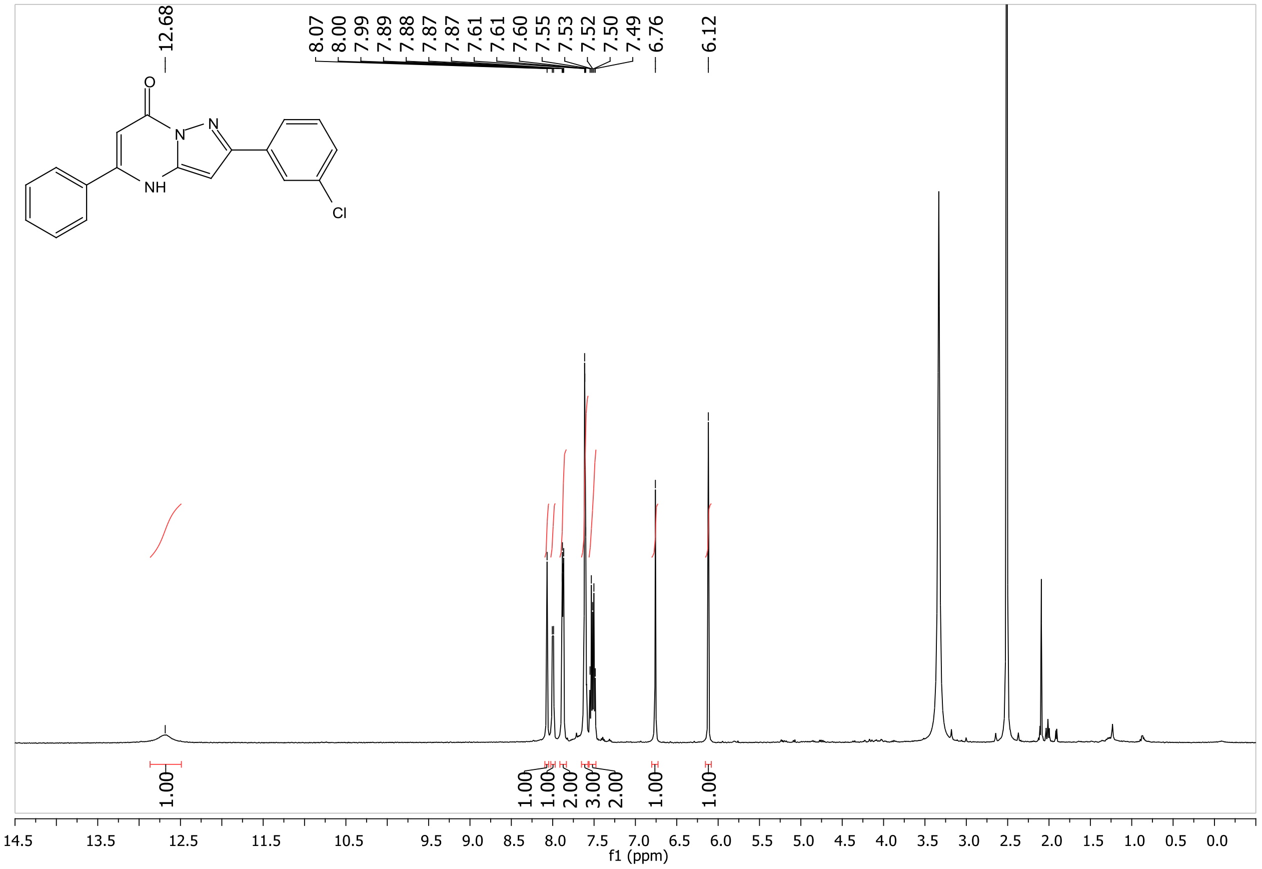

Figure S40. ^{1}H NMR spectrum of 2-(3-chlorophenyl)-5-phenylpyrazolo[1,5-*a*]pyrimidin-7(4*H*)-one (**3f**)

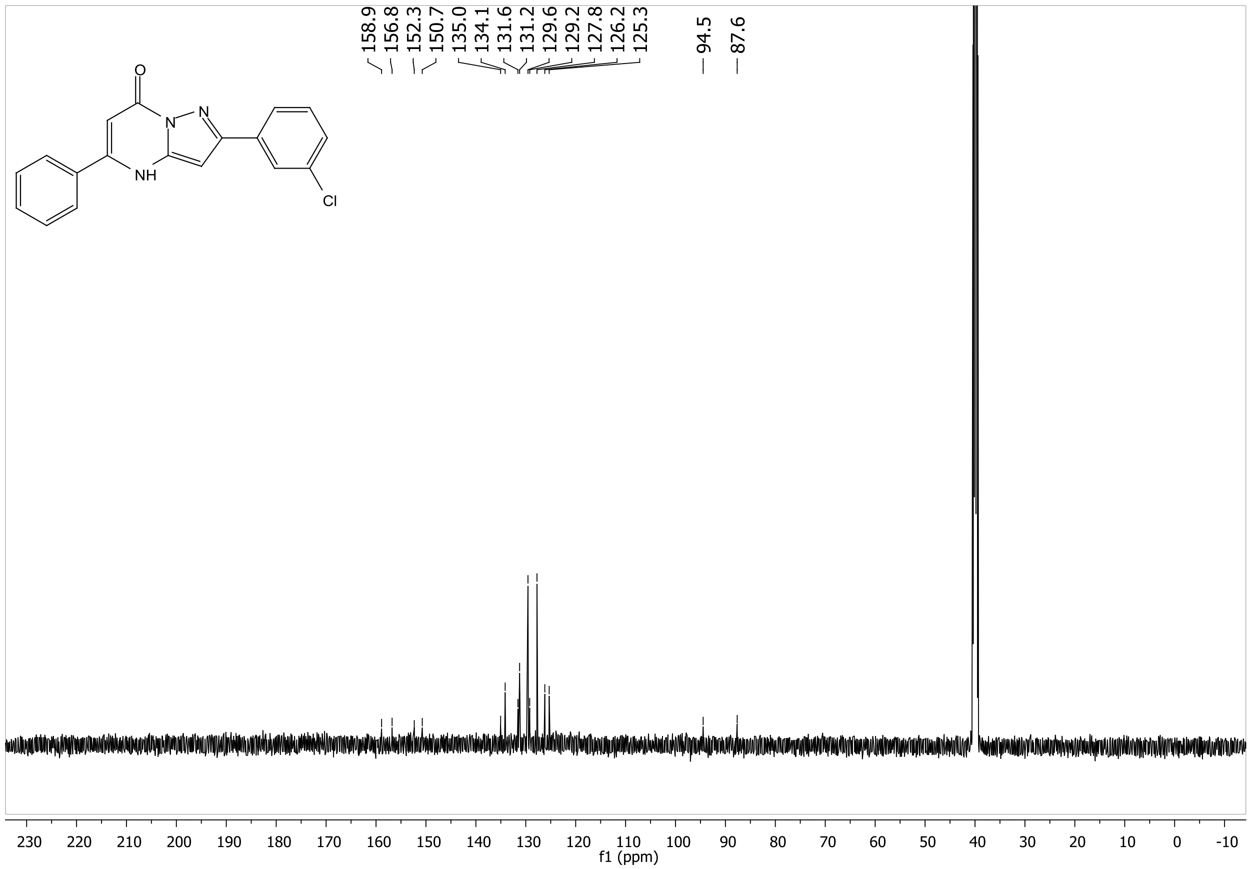

Figure S41. ^{13}C NMR spectrum of 2-(3-chlorophenyl)-5-phenylpyrazolo[1,5-*a*]pyrimidin-7(4*H*)-one (**3f**)

1.3.7. 2-(4-Fluorophenyl)-5-phenylpyrazolo[1,5-*a*]pyrimidin-7(4*H*)-one (3g)

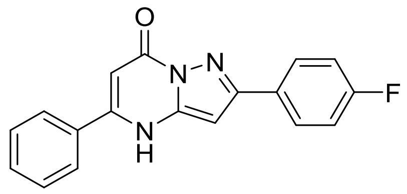

2-(4-Fluorophenyl)-5-phenylpyrazolo[1,5-*a*]pyrimidin-7(4*H*)-one (**3g**) was prepared as per general procedure and purified by trituration with cold MeOH. Orange solid; Yield (0.184 g, 67%); ^{1}H NMR (500 MHz, DMSO) δ 12.61 (bs, 1H, NH), 8.12 – 8.02 (m, 2H, Ar), 7.91 – 7.84 (m, 2H, Ar), 7.66 – 7.56 (m, 3H, Ar), 7.33 (m, 2H, Ar), 6.67 (s, 1H, CH), 6.10 (s, 1H, CHCO). ^{13}C NMR (126 MHz, DMSO) δ 163.1 (quaternary, d, J_{CF} = 246.3 Hz), 156.7 (quaternary), 152.9 (quaternary), 150.3 (quaternary), 143.7 (quaternary), 132.8 (quaternary), 131.6, 129.6, 129.4 (quaternary, d, J_{CF} = 3.0 Hz), 128.8 (d, J_{CF} = 8.4 Hz), 127.8, 116.2 (d, J_{CF} = 21.5 Hz), 94.6, 87.1. HRMS calcd for $C_{18}H_{13}FN_3O$ $[M + H]^+$: 306. 1037, found 306.1043. IR (KBr) 3060, 1667 (C=O), 1609, 1528, 767, 694. Matches literature MS data[11].

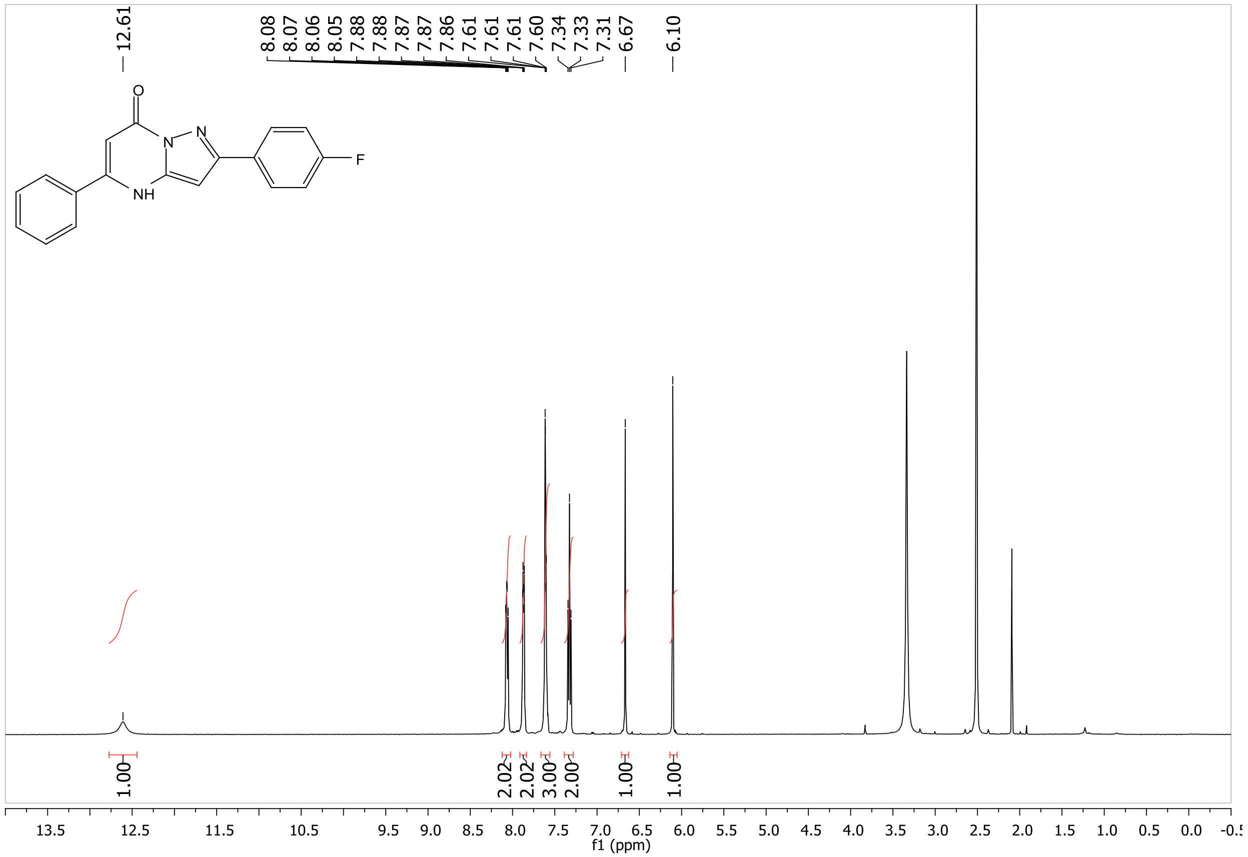

Figure S42. ^{1}H NMR spectrum of 2-(4-fluorophenyl)-5-phenylpyrazolo[1,5-*a*]pyrimidin-7(4*H*)-one (**3g**)

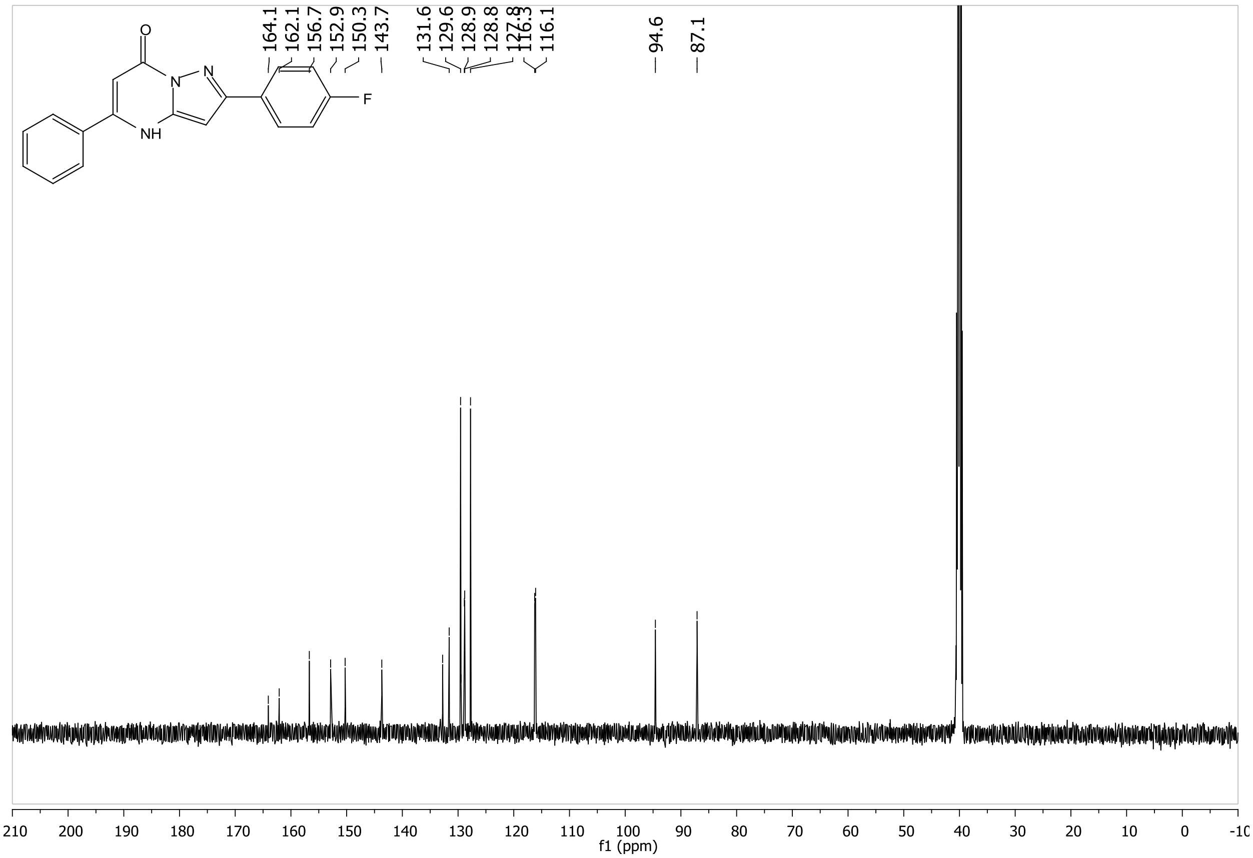

Figure S43. ^{13}C NMR spectrum of 2-(4-fluorophenyl)-5-phenylpyrazolo[1,5-*a*]pyrimidin-7(4*H*)-one (**3g**)

1.3.8. **5-Phenyl-2-(*p*-tolyl)pyrazolo[1,5-*a*]pyrimidin-7(4*H*)-one (3h)**

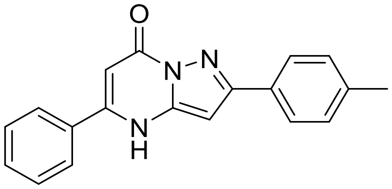

5-Phenyl-2-(*p*-tolyl)pyrazolo[1,5-*a*]pyrimidin-7(4*H*)-one (**3h**) was prepared as per general procedure and purified by trituration with cold MeOH. White solid; Yield (0.122 g, 45%); ^{1}H NMR (500 MHz, DMSO) δ 12.57 (bs, 1H, NH), 7.96 – 7.82 (m, 4*H*, Ar), 7.65 – 7.56 (m, 3H, Ar), 7.30 (d, *J* = 7.8 Hz, 2H, Ar), 6.62 (s, 1H, CH), 6.09 (s, 1H, CHCO), 2.37 (s, 3H, CH_3). ^{13}C NMR (126 MHz, DMSO) δ 156.7 (quaternary), 153.8 (quaternary), 150.2 (quaternary), 143.6 (quaternary), 138.9 (quaternary), 132.8 (quaternary), 131.5, 130.1 (quaternary), 129.8, 129.5, 127.7, 126.6, 94.5, 86.9, 21.4. HRMS calcd for $C_{19}H_{16}N_3O$ $[M + H]^+$: 302.1288, found 302.1291. IR (KBr) 3019, 1667 (C=O), 1609, 1448, 767, 689.

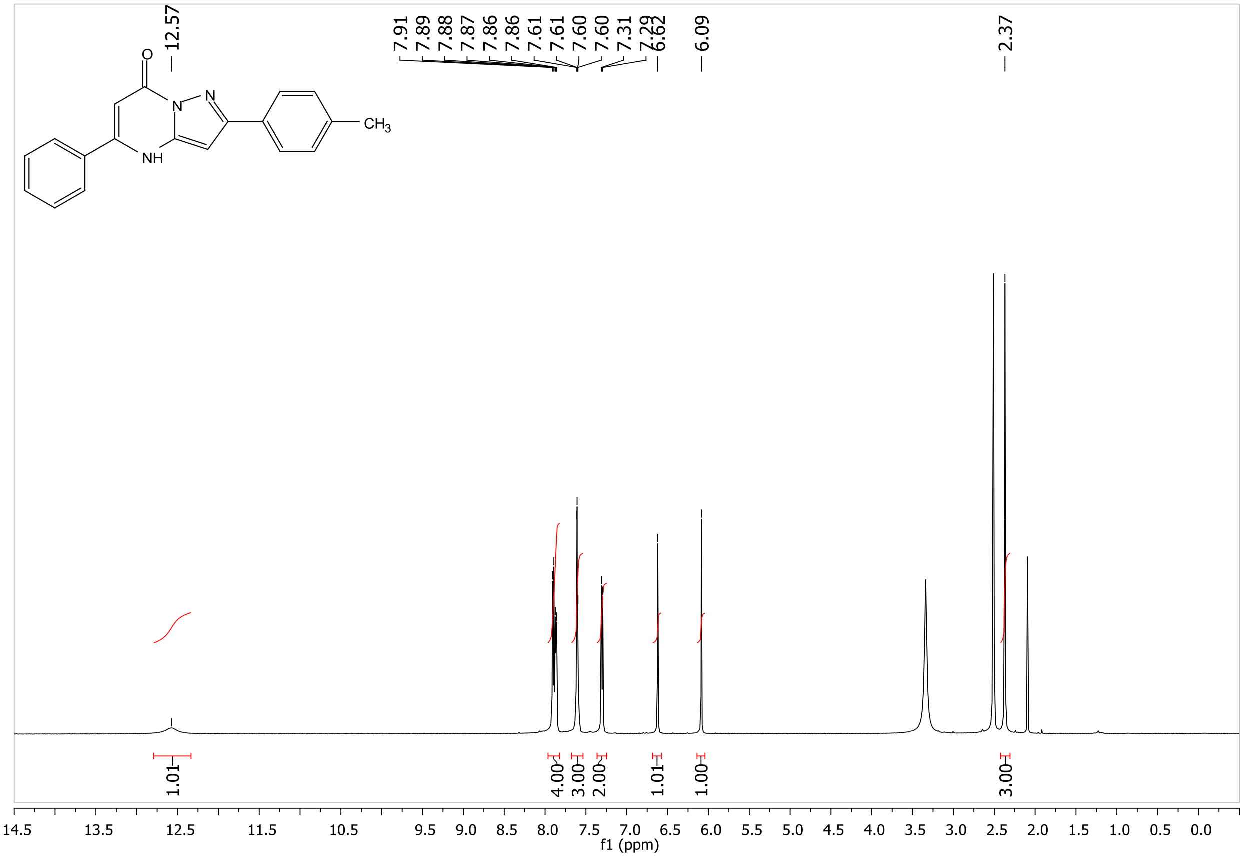

Figure S44. ^{1}H NMR spectrum of 5-phenyl-2-(*p*-tolyl)pyrazolo[1,5-*a*]pyrimidin-7(4*H*)-one (**3h**)

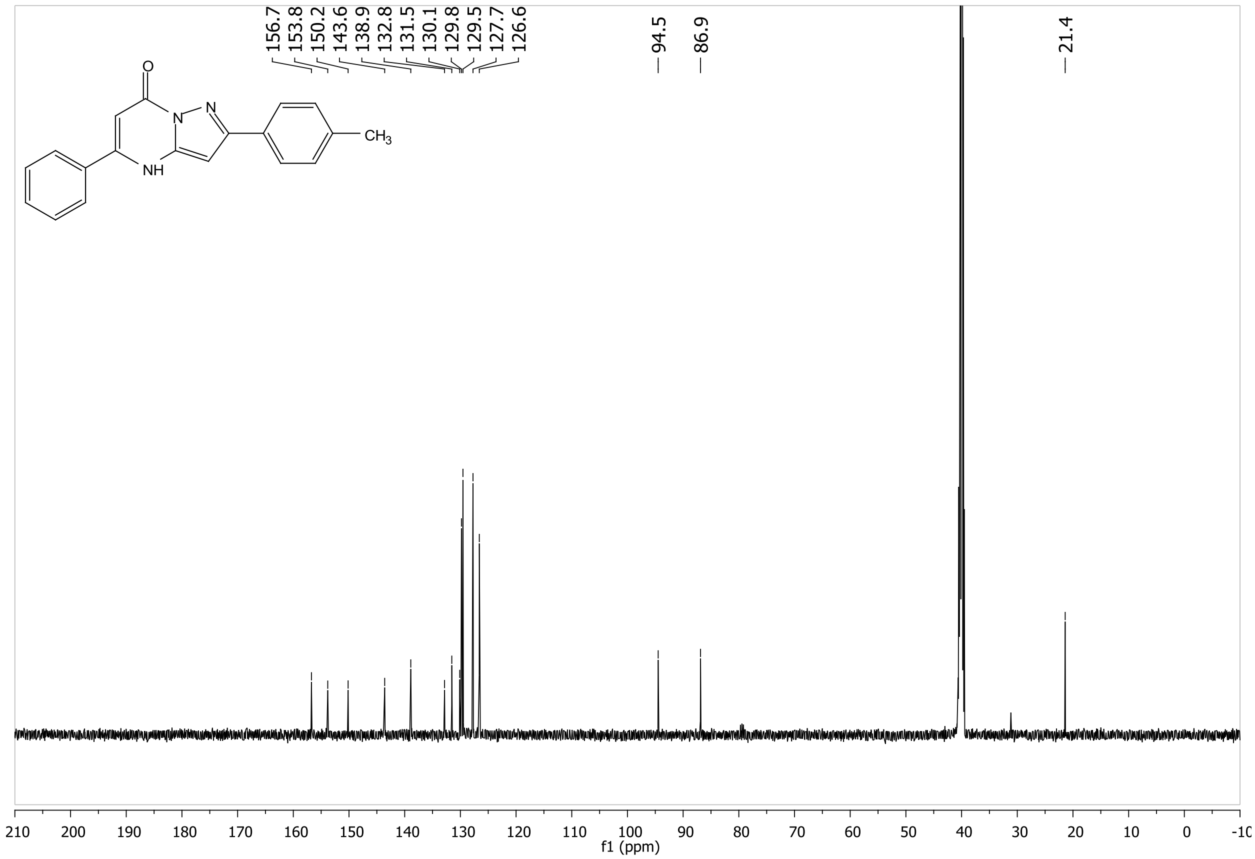

Figure S45. ^{13}C NMR spectrum of 5-phenyl-2-(*p*-tolyl)pyrazolo[1,5-*a*]pyrimidin-7(4*H*)-one (**3h**)

1.3.9. 2-(2-Methoxyphenyl)-5-phenylpyrazolo[1,5-*a*]pyrimidin-7(4*H*)-one (3i)

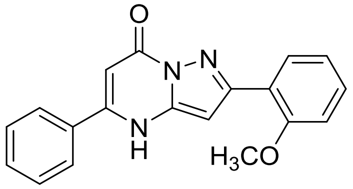

2-(2-Methoxyphenyl)-5-phenylpyrazolo[1,5-*a*]pyrimidin-7(4*H*)-one (**3i**) was prepared as per general procedure and purified by column chromatography (4:1 EtOAc:Petroluem Ether). White solid; Yield (0.092 g, 32%); Rf: 0.43 (3:2 EtOAc: Petroleum Ether); ^{1}H NMR (500 MHz, MeOD) δ 7.99 (bs, 1H, Ar), 7.85 (m, 2H, Ar), 7.48 (m, 3H, Ar), 7.30 (pseudo t, 1H, Ar), 7.04 (m, 1H, Ar), 6.97 (m, 1H, Ar), 6.73 (s, 1H, CH), 6.12 (s, 1H, CHCO), 3.89 (s, 3H, CH_3). ^{13}C NMR (126 MHz, DMSO) δ 159.1 (quaternary), 157.4 (quaternary), 156.4 (quaternary), 152.0 (quaternary), 149.4 (quaternary), 140.1 (quaternary), 129.4, 128.9, 128.7, 127.0, 123.3, 120.8, 112.3, 94.5, 89.0, 56.0.. HRMS calcd for $C_{19}H_{16}N_3O_2$ [M + H]$^+$: 318.1237, found 318.1241. IR (KBr) 3444, 1657 (C=O), 1602, 1419, 1248, 760.

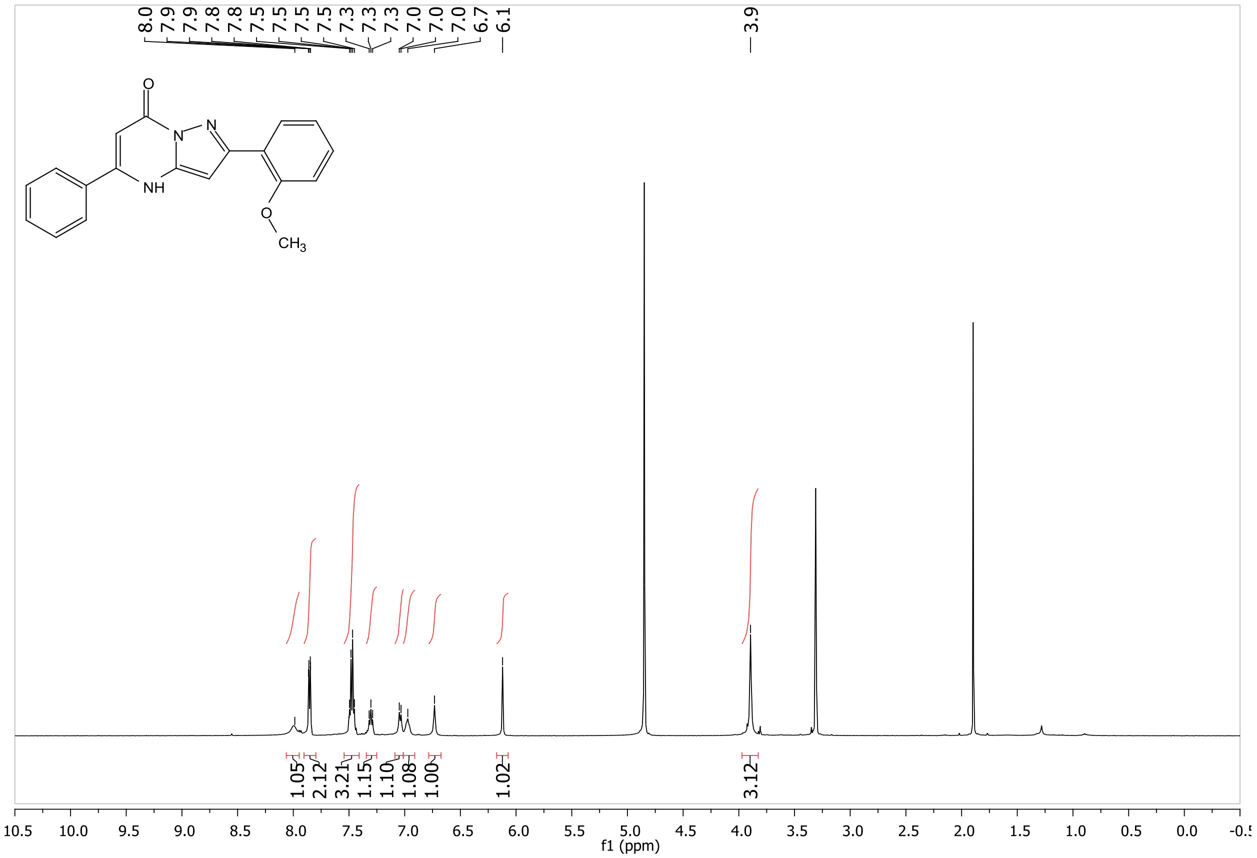

Figure S46. ^{1}H NMR spectrum of 2-(2-methoxyphenyl)-5-phenylpyrazolo[1,5-*a*]pyrimidin-7(4*H*)-one (**3i**)

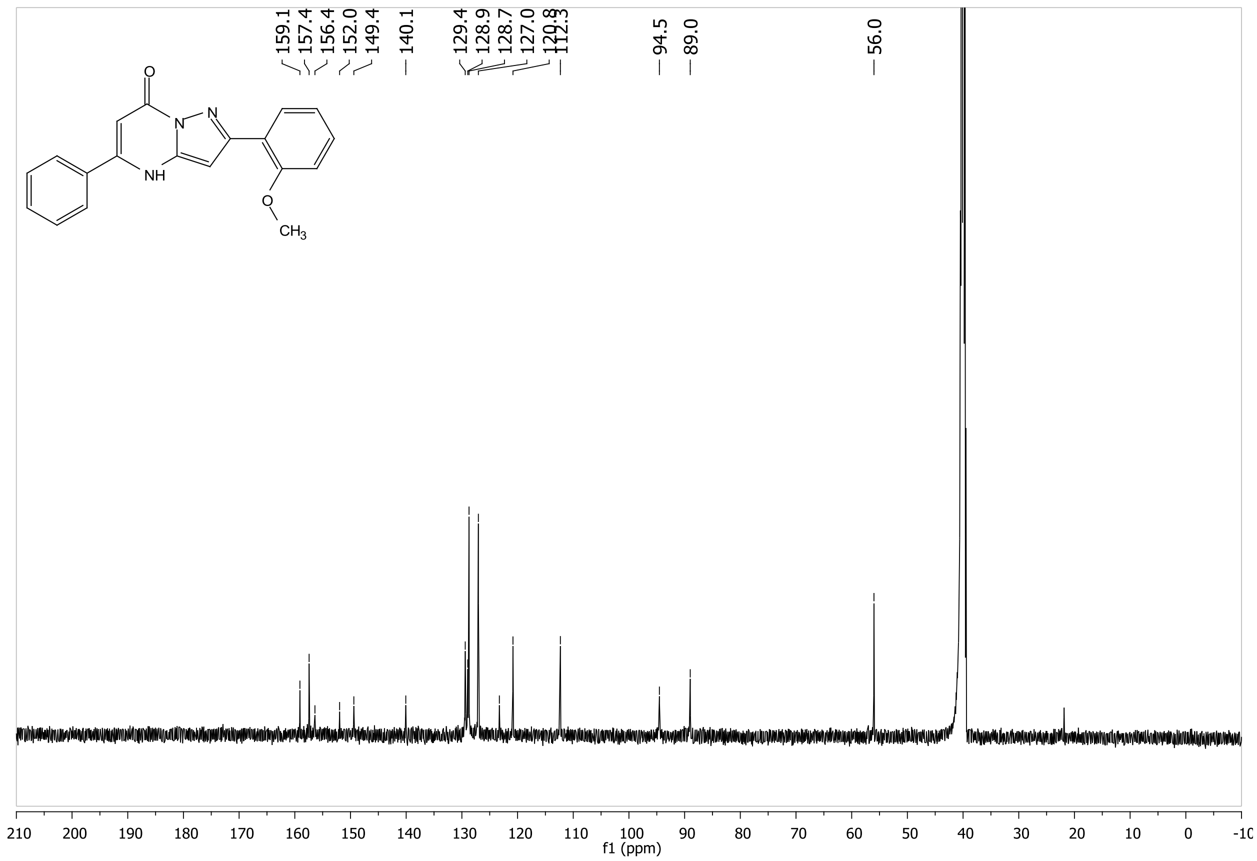

Figure S47. ^{13}C NMR spectrum of 2-(2-methoxyphenyl)-5-phenylpyrazolo[1,5-*a*]pyrimidin-7(4*H*)-one (**3i**)

1.3.10. 2-(*tert*-Butyl)-5-phenylpyrazolo[1,5-*a*]pyrimidin-7(4*H*)-one (3j)

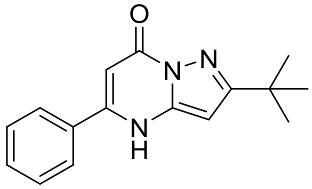

2-(*tert*-Butyl)-5-phenylpyrazolo[1,5-*a*]pyrimidin-7(4*H*)-one (**3j**) was prepared as per general procedure and purified by column chromatography (3:2 EtOAc:Petroluem Ether). White solid; Yield (0.041 g, 17%); Rf: 0.2 (3:2 EtOAc:Petroluem Ether); ^{1}H NMR (500 MHz, DMSO) δ 12.45 (bs, 1H, NH), 7.95 – 7.89 (m, 2H, Ar), 7.72 – 7.64 (m, 3H, Ar), 6.18 (s, 1H, CH), 6.09 (s, 1H, CHCO), 1.42 (s, 9H, 3 x CH_3). ^{13}C NMR (126 MHz, DMSO) δ 165.2 (quaternary), 156.9 (quaternary), 149.9 (quaternary), 142.8 (quaternary), 133.0 (quaternary), 131.4, 129.5, 127.7, 94.1, 86.5, 30.6. HRMS calcd for $C_{16}H_{18}N_3O$ [M + H]$^+$: 268.1444, found 268.1442. IR (KBr) 2966, 1659 (C=O), 1611, 1324, 816, 770.

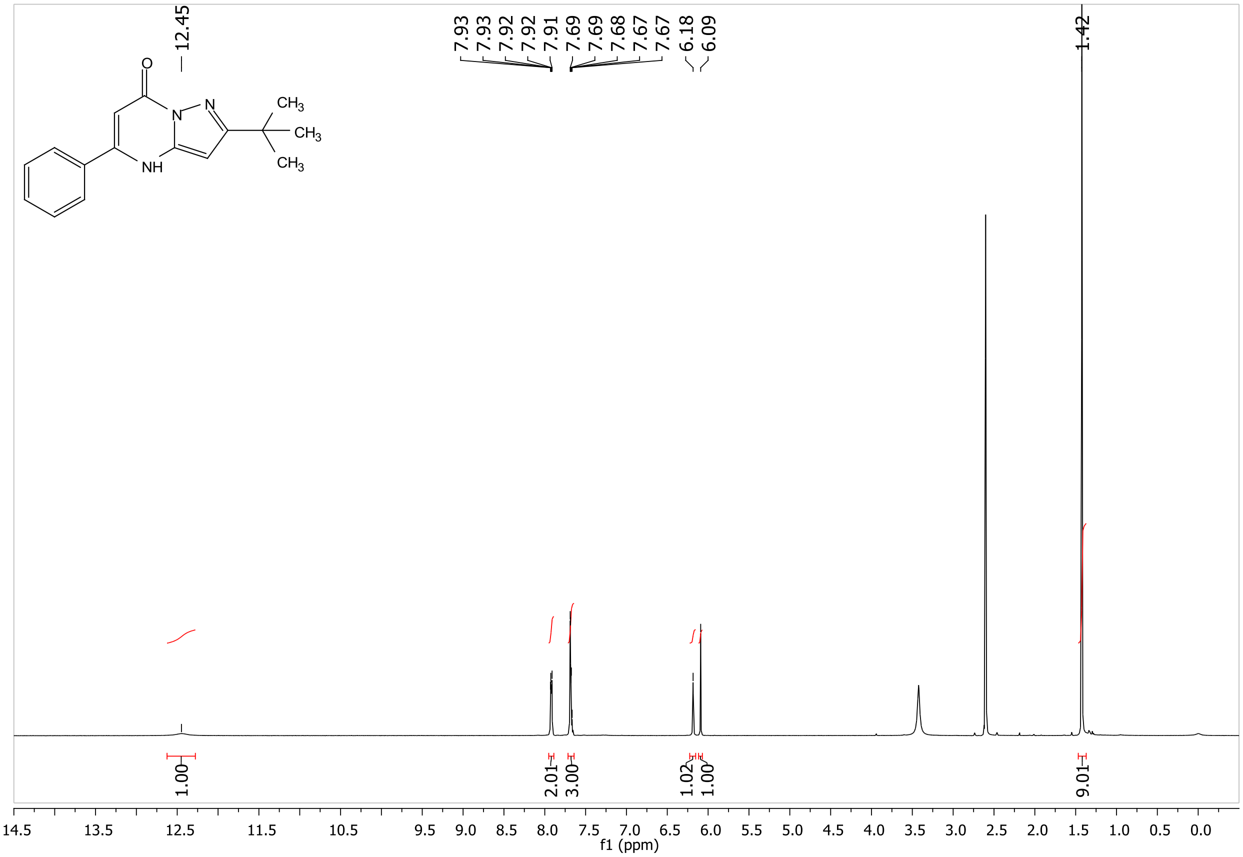

Figure S48. ^{1}H NMR spectrum of 2-(tert-butyl)-5-phenylpyrazolo[1,5-*a*]pyrimidin-7(4*H*)-one (**3j**)

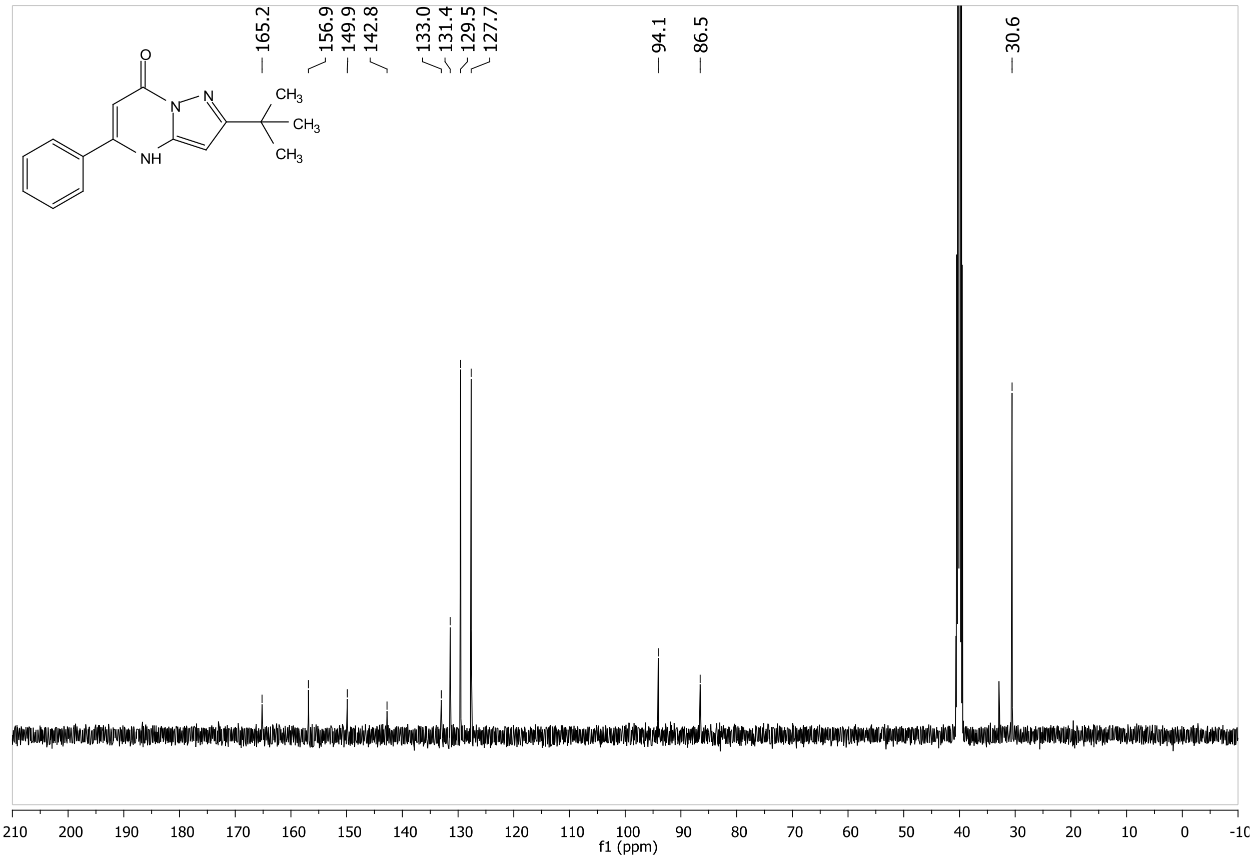

Figure S49. ^{13}C NMR spectrum of 2-(tert-butyl)-5-phenylpyrazolo[1,5-*a*]pyrimidin-7(4*H*)-one (**3j**)

1.3.11. 2-Methyl-5-phenylpyrazolo[1,5-*a*]pyrimidin-7(4*H*)-one (3k)

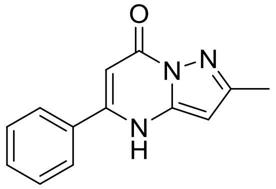

2-Methyl-5-phenylpyrazolo[1,5-*a*]pyrimidin-7(4*H*)-one (**3k**) was prepared as per general procedure and purified by trituration with EtOAc. Off-white solid; Yield (0.066 g, 33%); ^{1}H NMR (500 MHz, DMSO) δ 12.34 (bs, 1H), 7.86 – 7.81 (m, 2H, Ar), 7.62 – 7.55 (m, 3H, Ar), 6.05 (s, 1H, CH), 6.00 (s, 1H, CHCO), 2.32 (s, 3H, CH_3). ^{13}C NMR (126 MHz, DMSO) δ 156.6 (quaternary), 152.6 (quaternary), 149.7 (quaternary), 142.9 (quaternary), 132.9 (quaternary), 131.5, 129.5, 127.6, 94.1, 89.7, 14.6. HRMS calcd for $C_{13}H_{12}N_3O$ $[M + H]^+$: 226.0975, found 226.0979. IR (KBr) 3441, 3088, 1674 (C=O), 771. Matches literature data[10].

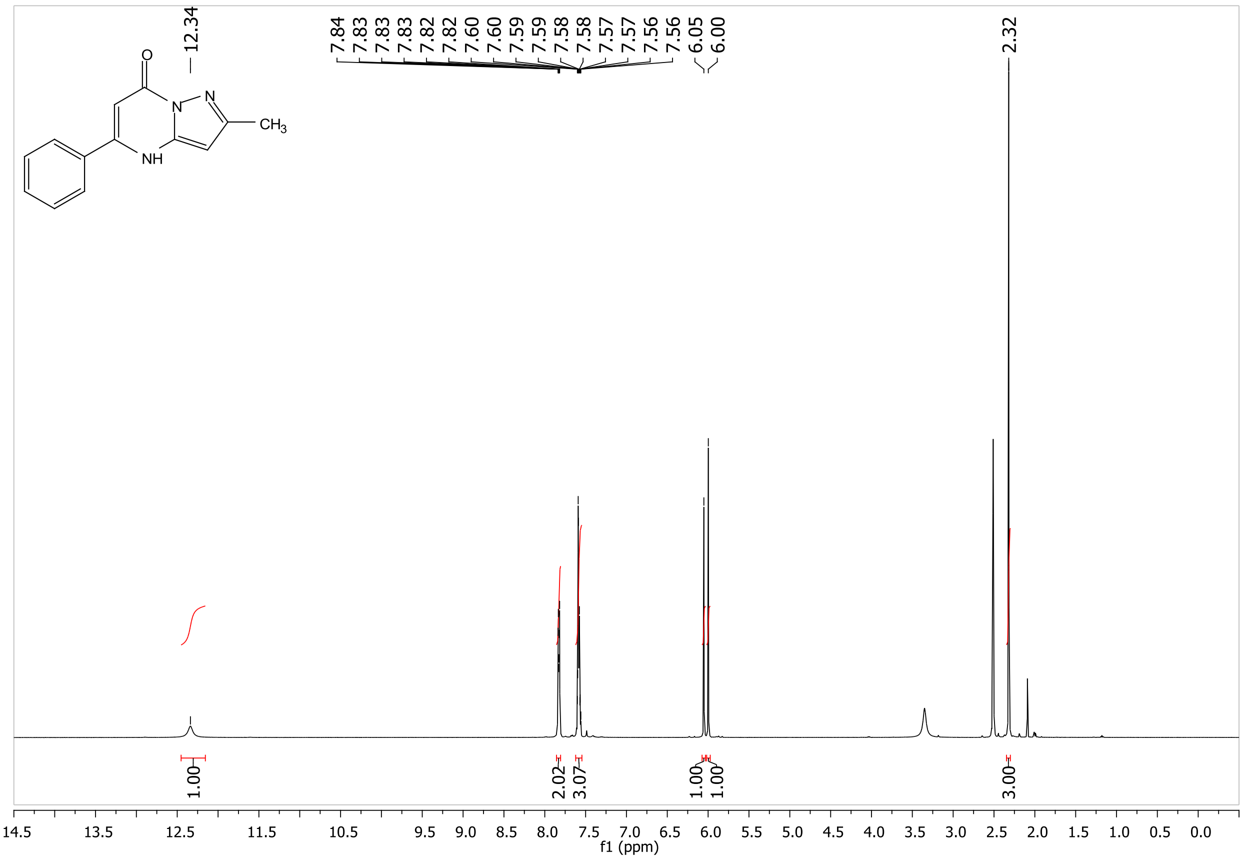

Figure S50. ^{1}H NMR spectrum of 2-methyl-5-phenylpyrazolo[1,5-*a*]pyrimidin-7(4*H*)-one (**3k**)

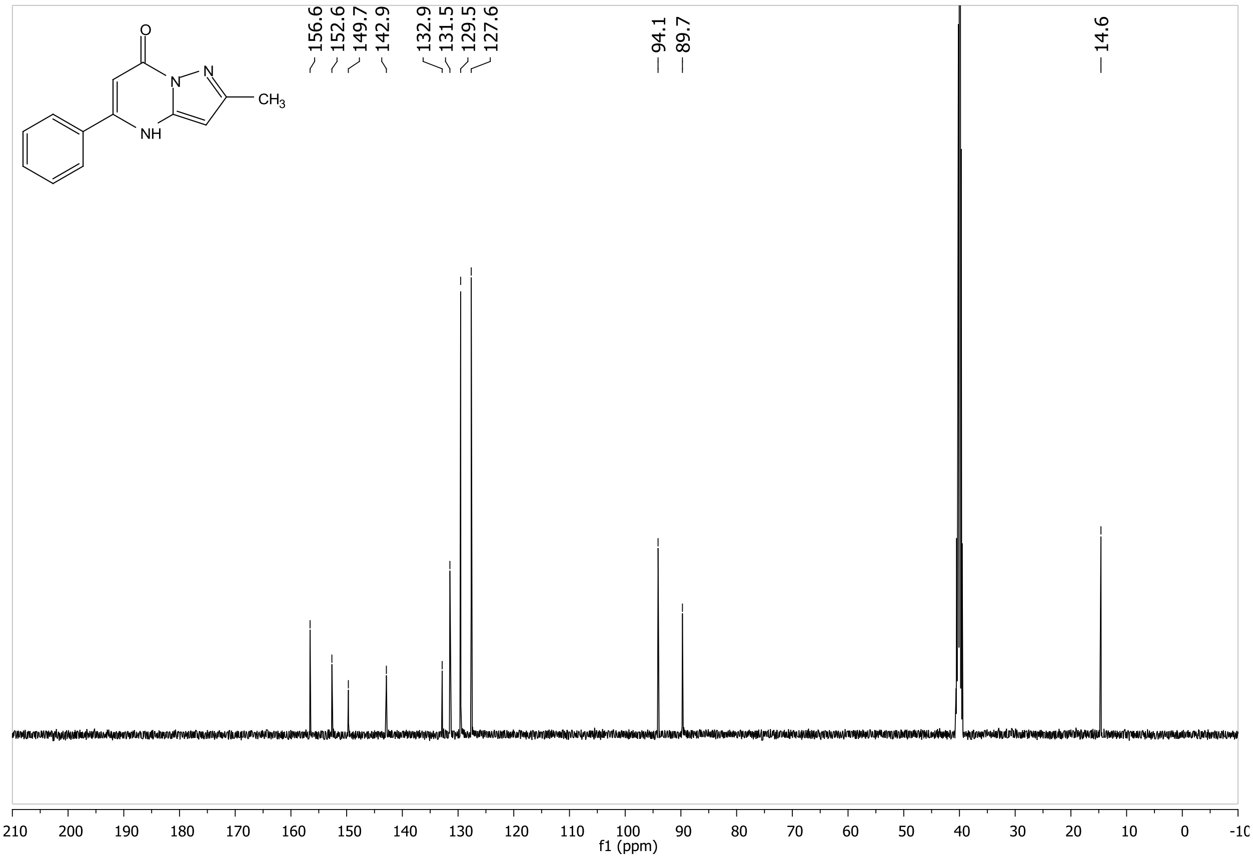

Figure S51. ^{13}C NMR spectrum of 2-methyl-5-phenylpyrazolo[1,5-*a*]pyrimidin-7(4*H*)-one (**3k**)

1.3.12. 5-Phenyl-2-(thiophen-2-yl)pyrazolo[1,5-*a*]pyrimidin-7(4*H*)-one (3l)

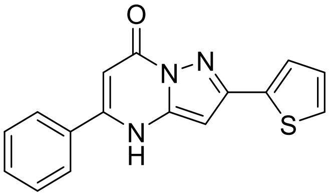

5-Phenyl-2-(thiophen-2-yl)pyrazolo[1,5-*a*]pyrimidin-7(4*H*)-one (**3l**) was purified by column chromatography (3:2 EtOAc: Petroleum Ether). Yellow solid; Yield (0.105 g, 40%); Rf: 0.43 (3:2 EtOAc: Petroleum Ether); ^{1}H NMR (500 MHz, DMSO) δ 7.98 (d, *J* = 6.8 Hz, 2H, Ar), 7.61 (d, *J* = 3.5 Hz, 1H, thiophene), 7.53 (d, *J* = 5.0 Hz, 1H, thiophene), 7.51 – 7.38 (m, 3H, Ar), 7.13 (dd, *J* = 5.0, 3.5 Hz, 1H, thiophene), 6.46 (s, 1H, CH), 6.09 (s, 1H, CHCO). ^{13}C NMR (126 MHz, DMSO) δ 158.4 (quaternary), 155.6 (quaternary), 150.8 (quaternary), 148.3 (quaternary), 138.4 (quaternary), 137.7 (quaternary), 129.6, 128.9, 128.1, 127.2, 126.2, 125.7, 90.7, 89.4. HRMS calcd for $C_{16}H_{12}N_3OS$ [M + H]$^+$: 294.0696, found 294.0696. IR (KBr) 3402, 1667 (C=O), 1661, 768, 693.

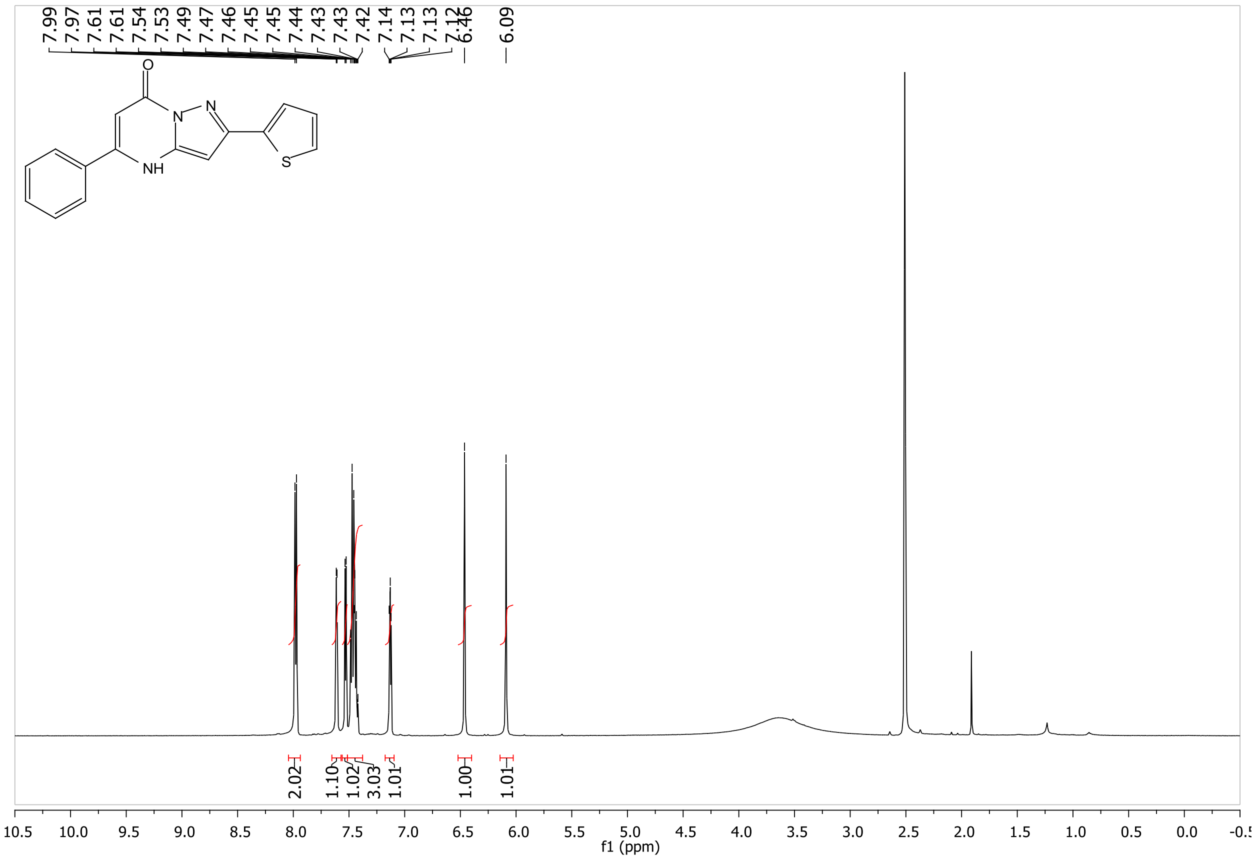

Figure S52. ^{1}H NMR spectrum of 5-phenyl-2-(thiophen-2-yl)pyrazolo[1,5-*a*]pyrimidin-7(4*H*)-one (**3l**)

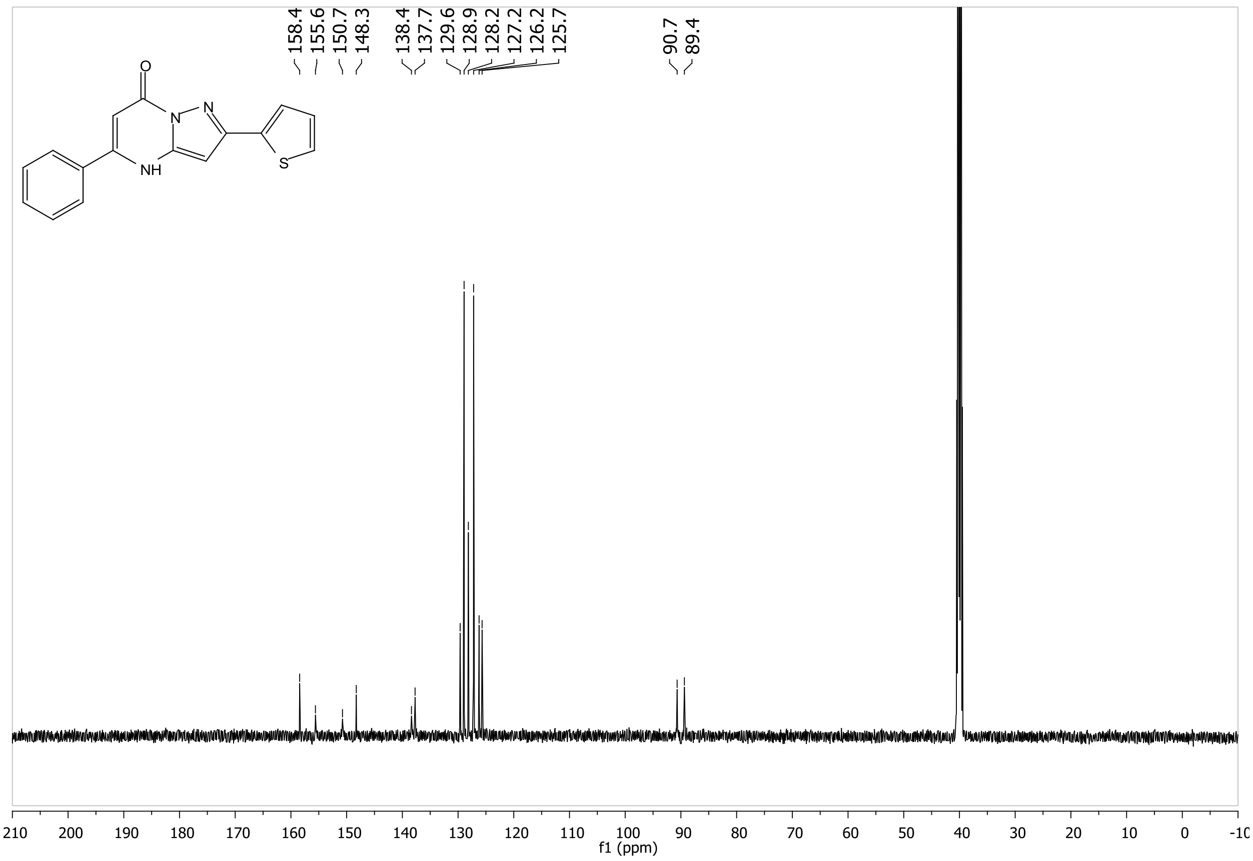

Figure S53. **^{13}C NMR spectrum of 5-phenyl-2-(thiophen-2-yl)pyrazolo[1,5-*a*]pyrimidin-7(4*H*)-one (3l)**

1.3.13. 2-(Furan-2-yl)-5-phenylpyrazolo[1,5-*a*]pyrimidin-7(4*H*)-one (3m)

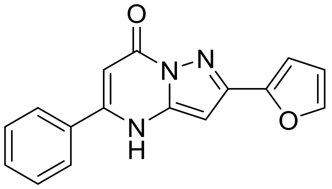

2-(Furan-2-yl)-5-phenylpyrazolo[1,5-*a*]pyrimidin-7(4*H*)-one (**3m**) was prepared as per general procedure and purified by column chromatography (9:1 DCM: MeOH). Brown solid; Yield (0.2 g, 50%); Rf: 0.5 (9:1 DCM: MeOH); ^{1}H NMR (500 MHz, DMSO) δ 12.58 (bs, 1H, NH), 7.90 – 7.85 (m, 3H, Ar), 7.84 (dd, *J* = 1.7, 0.6 Hz, 1H, Ar), 7.64 – 7.56 (m, 3H, Ar), 7.04 (dd, *J* = 3.3, 0.6 Hz, 1H, furan), 6.66 (dd, *J* = 3.4, 1.7 Hz, 1H, furan), 6.45 (s, 1H, CH), 6.10 (s, 1H, CHCO). ^{13}C NMR (126 MHz, DMSO) δ 156.7 (quaternary), 150.5 (quaternary), 148.3 (quaternary), 146.2 (quaternary), 144.2, 143.6 (quaternary), 133.0 (quaternary), 131.5, 129.5, 127.7, 112.4, 109.0, 94.5, 86.8. HRMS calcd for $C_{16}H_{12}N_3O_2$ [M + H]$^+$: 278.0924, found 278.0932. IR (KBr) 3122, 3059, 1669 (C=O), 764.

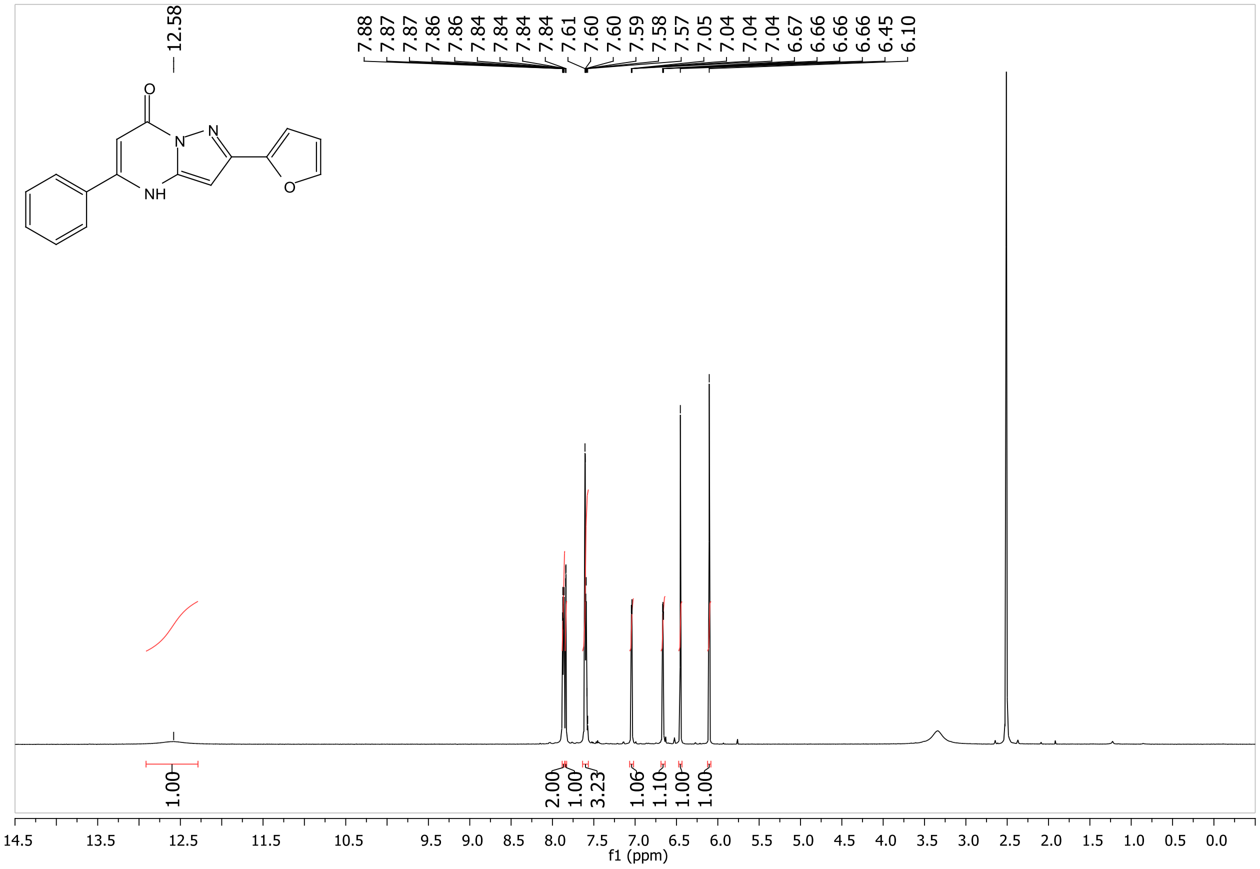

Figure S54. ^{1}H NMR spectrum of 2-(furan-2-yl)-5-phenylpyrazolo[1,5-*a*]pyrimidin-7(4*H*)-one (**3m**)

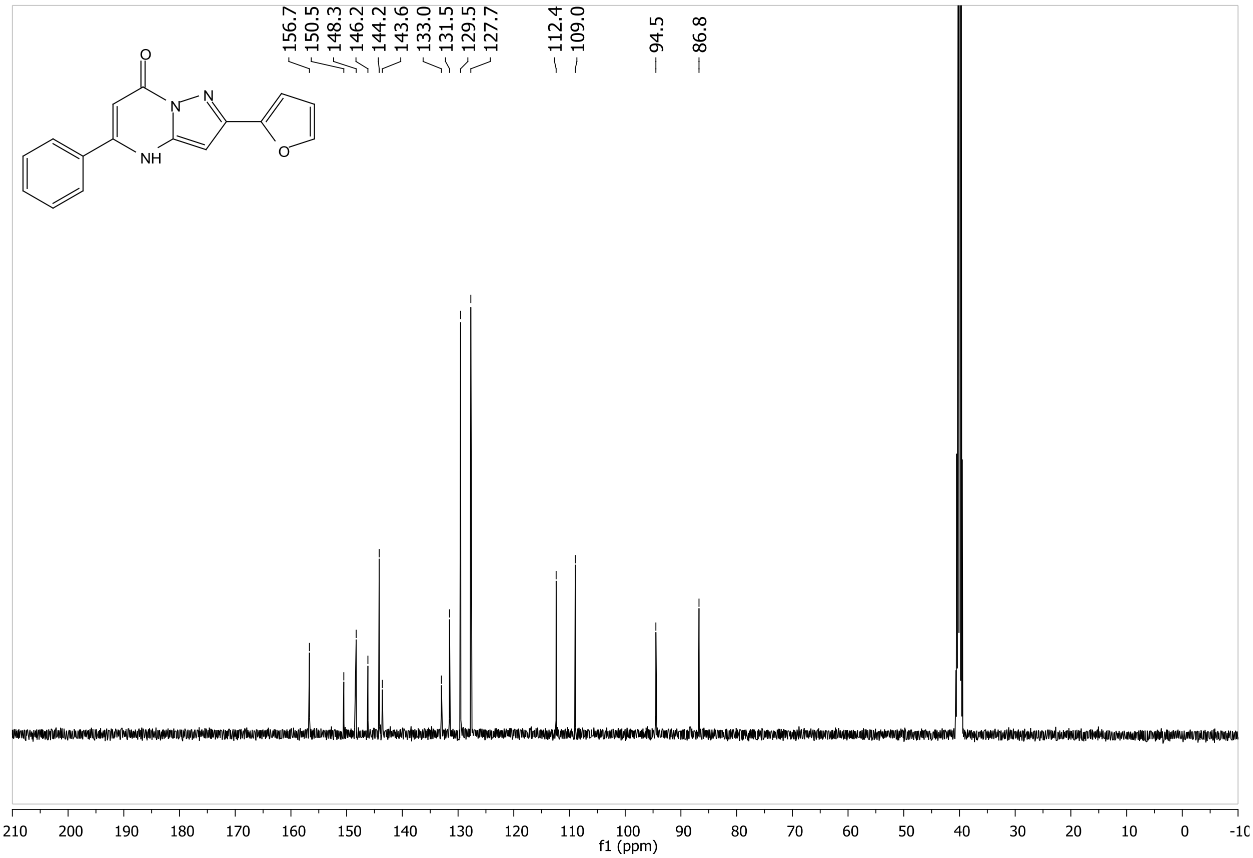

Figure S55. ^{13}C NMR spectrum of 2-(furan-2-yl)-5-phenylpyrazolo[1,5-*a*]pyrimidin-7(4*H*)-one (**3m**)

1.3.14. 5-(3,5-Bis(trifluoromethyl)phenyl)-2-(4-nitrophenyl)pyrazolo[1,5-*a*]pyrimidin-7(4*H*)-one (3n)

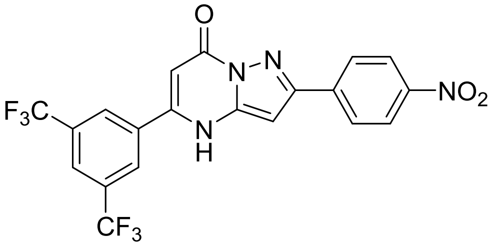

5-(3,5-Bis(trifluoromethyl)phenyl)-2-(4-nitrophenyl)pyrazolo[1,5-*a*]pyrimidin-7(4*H*)-one (**3n**) was prepared as per general procedure and purified by trituration with cold MeOH. Yellow solid; Yield (0.33 g, 78%); ^{1}H NMR (500 MHz, DMSO) δ 12.99 (s, 1H, NH), 8.56 (s, 2H, Ar), 8.46 – 8.24 (m, 5H, Ar), 6.91 (s, 1H, CH), 6.46 (s, 1H, CHCO). ^{13}C NMR (126 MHz, DMSO) δ 156.4 (quaternary), 151.9 (quaternary), 148.0 (quaternary), 147.6 (quaternary), 143.8 (quaternary), 139.1 (quaternary), 135.3 (quaternary), 131.4 (quaternary, q, J = 33.4 Hz, CF_3), 129.1, 127.8, 125.0, 124.5, 122.4, 96.6, 88.5. HRMS calcd for $C_{20}H_{10}F_6N_4O_3Na$ $[M + Na]^+$: 491.0549, found 491.0544. IR (ATR) 3067, 2927, 1667 (C=O), 1612, 1601, 1516 (N-O), 1438, 1364 (N-O), 1129 (C-F), 1112 (C-F), 682.

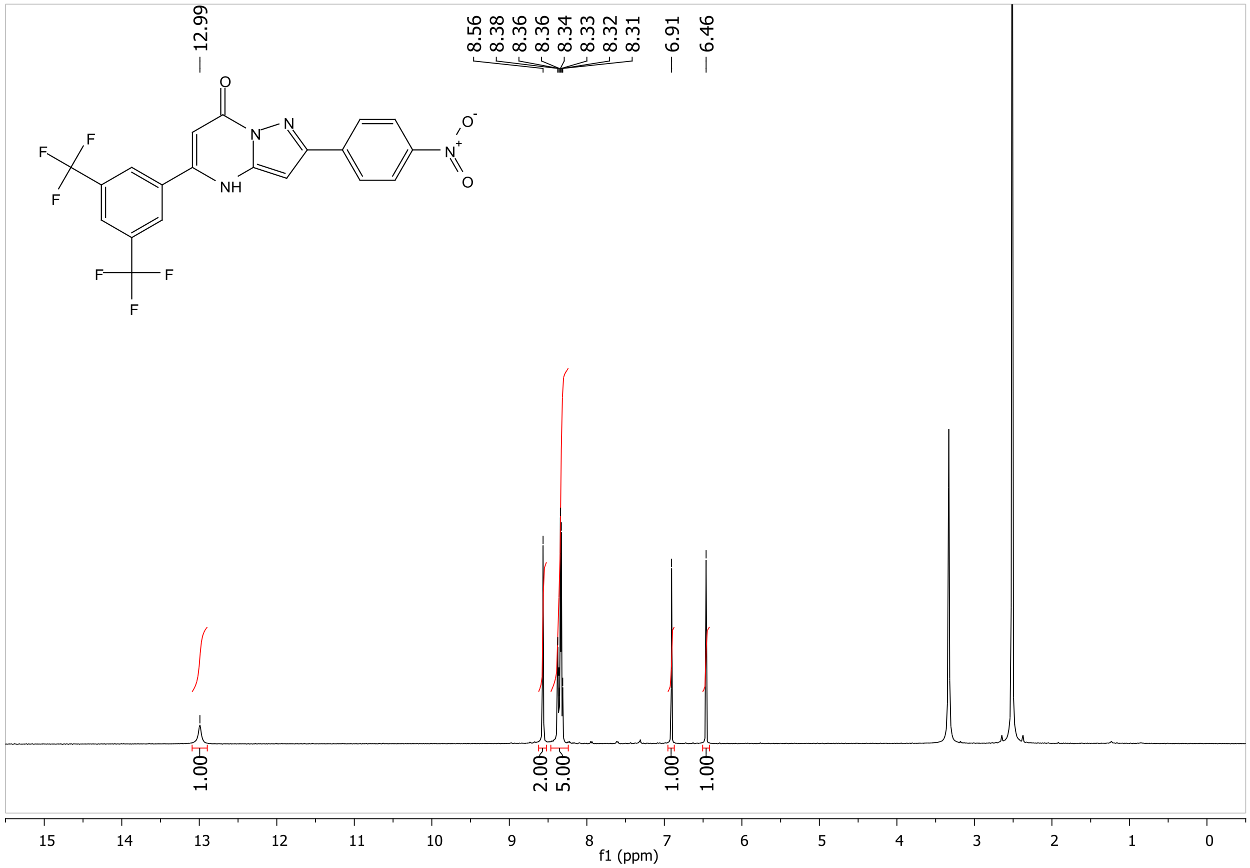

Figure S56. ^{1}H NMR spectrum of 5-(3,5-bis(trifluoromethyl)phenyl)-2-(4-nitrophenyl)pyrazolo[1,5-*a*]pyrimidin-7(4*H*)-one (**3n**)

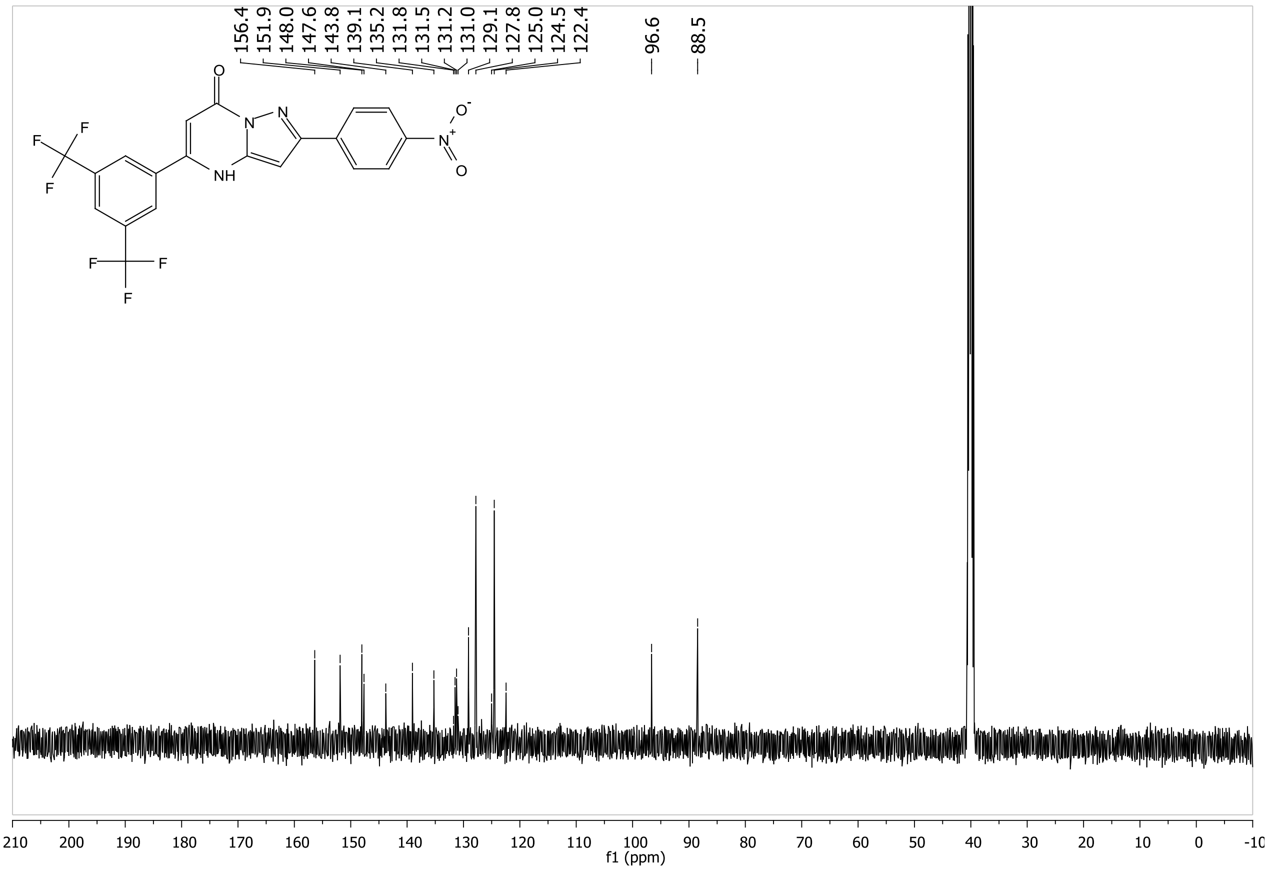

Figure S57. ^{13}C NMR spectrum of 5-(3,5-bis(trifluoromethyl)phenyl)-2-(4-nitrophenyl)pyrazolo[1,5-*a*]pyrimidin-7(4*H*)-one (**3n**)

2. X-ray crystallographic data

An Oxford Diffraction Xcalibur system was used to collect X-ray diffraction data at room temperature. The crystal structures were solved using ShelxT and refined using ShelxL within the Oscail package[12-13].

Crystallographic data (excluding structure factors) for structure **3m** (MK56b) have been deposited with the Cambridge Crystallographic Data Centre as supplementary publication CCDC 1588686. Copies of the data can be obtained, free of charge, on application to CCDC, 12 Union Road, Cambridge CB2 1EZ, UK, (fax: +44-(0)1223-336033 or e-mail: deposit@ccdc.cam.ac.uk).

Table S1. Crystal data and structure refinement for mk56b.

Identification code	mk56b	
Empirical formula	C16 H11 N3 O2	
Formula weight	277.28	
Temperature	298.5(9) K	
Wavelength	0.71073 Å	
Crystal system	Triclinic	
Space group	P-1	
Unit cell dimensions	a = 6.6464(6) Å	α= 82.880(10)°.
	b = 7.0114(9) Å	β= 86.962(8)°.
	c = 15.0626(16) Å	γ = 72.352(10)°.
Volume	663.64(13) Å^3	
Z	2	
Density (calculated)	1.388 Mg/m^3	
Absorption coefficient	0.095 mm^{-1}	
F(000)	288	
Crystal size	0.50 x 0.40 x 0.02 mm^3	
Theta range for data collection	3.723 to 29.168°.	
Index ranges	-8<=h<=7, -9<=k<=9, -16<=l<=20	
Reflections collected	4838	
Independent reflections	3011 [R(int) = 0.0233]	
Completeness to theta = 25.242°	99.7%	
Absorption correction	Semi-empirical from equivalents	
Max. and min. transmission	1.00000 and 0.69643	
Refinement method	Full-matrix least-squares on F^2	
Data / restraints / parameters	3011 / 1 / 195	
Goodness-of-fit on F^2	1.048	
Final R indices [I>2sigma(I)]	R1 = 0.0593, wR2 = 0.1313	
R indices (all data)	R1 = 0.1145, wR2 = 0.1614	
Extinction coefficient	0.010(4)	
Largest diff. peak and hole	0.203 and -0.167 e.Å^{-3}	

Table S2. Atomic coordinates (× 10^4) and equivalent isotropic displacement parameters (Å^{2}x 10^3) for mk56b. U(eq) is defined as one third of the trace of the orthogonalized U^{ij} tensor.

	x	y	z	U(eq)
O(1)	1978(2)	2474(3)	5142(1)	69(1)
N(2)	5084(2)	2450(3)	5702(1)	43(1)
N(1)	8044(2)	2440(3)	4783(1)	42(1)
N(3)	4405(3)	2463(3)	6565(1)	55(1)
O(2)	7624(5)	2616(4)	8381(2)	112(1)
C(1)	6930(3)	2451(3)	4051(2)	42(1)
C(2)	4879(3)	2453(3)	4156(2)	46(1)
C(3)	3826(3)	2448(3)	4985(2)	47(1)
C(4)	6113(4)	2433(4)	7007(2)	56(1)
C(5)	7855(4)	2418(4)	6447(2)	50(1)
C(6)	7159(3)	2425(3)	5612(2)	40(1)
C(7)	8036(4)	2469(4)	3182(2)	50(1)
C(8)	9609(4)	3399(4)	3023(2)	62(1)
C(9)	10656(5)	3352(5)	2210(2)	89(1)
C(10)	10129(7)	2453(6)	1545(2)	110(1)
C(11)	8543(7)	1565(6)	1691(2)	107(1)
C(12)	7509(5)	1561(5)	2504(2)	75(1)
C(13)	5920(5)	2488(4)	7964(2)	74(1)
C(14)	4379(7)	2428(6)	8561(3)	112(1)
C(15)	5162(11)	2539(8)	9408(3)	148(2)
C(16)	7086(11)	2670(8)	9265(3)	153(2)

Table S3. Bond lengths [Å] and angles [°] for mk56b.

O(1)-C(3)	1.233(2)
N(2)-N(3)	1.354(2)
N(2)-C(6)	1.374(2)
N(2)-C(3)	1.400(3)
N(1)-C(6)	1.352(3)
N(1)-C(1)	1.358(3)
N(1)-H(1N1)	0.898(16)
N(3)-C(4)	1.340(3)
O(2)-C(13)	1.356(4)
O(2)-C(16)	1.363(4)
C(1)-C(2)	1.363(3)
C(1)-C(7)	1.468(3)
C(2)-C(3)	1.399(3)
C(2)-H(2)	0.9300
C(4)-C(5)	1.395(3)
C(4)-C(13)	1.444(4)
C(5)-C(6)	1.361(3)
C(5)-H(5)	0.9300
C(7)-C(12)	1.380(3)
C(7)-C(8)	1.387(3)
C(8)-C(9)	1.374(4)
C(8)-H(8)	0.9300
C(9)-C(10)	1.360(4)
C(9)-H(9)	0.9300
C(10)-C(11)	1.373(4)
C(10)-H(10)	0.9300
C(11)-C(12)	1.373(4)
C(11)-H(11)	0.9300
C(12)-H(12)	0.9300
C(13)-C(14)	1.334(4)
C(14)-C(15)	1.422(6)
C(14)-H(14)	0.9300
C(15)-C(16)	1.314(6)

C(15)-H(15)	0.9300
C(16)-H(16)	0.9300
N(3)-N(2)-C(6)	111.99(17)
N(3)-N(2)-C(3)	124.04(16)
C(6)-N(2)-C(3)	123.97(18)
C(6)-N(1)-C(1)	121.26(16)
C(6)-N(1)-H(1N1)	120.1(16)
C(1)-N(1)-H(1N1)	118.6(16)
C(4)-N(3)-N(2)	103.34(17)
C(13)-O(2)-C(16)	106.5(3)
N(1)-C(1)-C(2)	119.2(2)
N(1)-C(1)-C(7)	116.95(18)
C(2)-C(1)-C(7)	123.8(2)
C(1)-C(2)-C(3)	123.7(2)
C(1)-C(2)-H(2)	118.2
C(3)-C(2)-H(2)	118.2
O(1)-C(3)-C(2)	128.1(2)
O(1)-C(3)-N(2)	118.6(2)
C(2)-C(3)-N(2)	113.33(17)
N(3)-C(4)-C(5)	113.1(2)
N(3)-C(4)-C(13)	118.0(2)
C(5)-C(4)-C(13)	128.9(2)
C(6)-C(5)-C(4)	104.43(19)
C(6)-C(5)-H(5)	127.8
C(4)-C(5)-H(5)	127.8
N(1)-C(6)-C(5)	134.34(18)
N(1)-C(6)-N(2)	118.54(18)
C(5)-C(6)-N(2)	107.12(19)
C(12)-C(7)-C(8)	118.9(2)
C(12)-C(7)-C(1)	120.0(2)
C(8)-C(7)-C(1)	121.1(2)
C(9)-C(8)-C(7)	119.9(3)
C(9)-C(8)-H(8)	120.0
C(7)-C(8)-H(8)	120.0

C(10)-C(9)-C(8)	120.9(3)
C(10)-C(9)-H(9)	119.6
C(8)-C(9)-H(9)	119.6
C(9)-C(10)-C(11)	119.6(3)
C(9)-C(10)-H(10)	120.2
C(11)-C(10)-H(10)	120.2
C(12)-C(11)-C(10)	120.4(3)
C(12)-C(11)-H(11)	119.8
C(10)-C(11)-H(11)	119.8
C(11)-C(12)-C(7)	120.3(3)
C(11)-C(12)-H(12)	119.8
C(7)-C(12)-H(12)	119.8
C(14)-C(13)-O(2)	109.8(3)
C(14)-C(13)-C(4)	133.6(3)
O(2)-C(13)-C(4)	116.5(3)
C(13)-C(14)-C(15)	106.5(4)
C(13)-C(14)-H(14)	126.7
C(15)-C(14)-H(14)	126.7
C(16)-C(15)-C(14)	106.6(4)
C(16)-C(15)-H(15)	126.7
C(14)-C(15)-H(15)	126.7
C(15)-C(16)-O(2)	110.5(4)
C(15)-C(16)-H(16)	124.7
O(2)-C(16)-H(16)	124.7

Symmetry transformations used to generate equivalent atoms:

Table S4. Anisotropic displacement parameters ($Å^2$x 10^3) for mk56b. The anisotropic displacement factor exponent takes the form: $-2\pi^2[h^2 a^{*2}U^{11} + ... + 2 h k a^* b^* U^{12}]$

	U^{11}	U^{22}	U^{33}	U^{23}	U^{13}	U^{12}
O(1)	26(1)	87(1)	100(2)	-6(1)	-5(1)	-26(1)
N(2)	29(1)	46(1)	58(1)	-7(1)	2(1)	-15(1)
N(1)	23(1)	48(1)	56(1)	-1(1)	-7(1)	-14(1)
N(3)	52(1)	52(1)	65(2)	-10(1)	11(1)	-21(1)
O(2)	174(3)	130(2)	63(2)	-3(1)	-20(2)	-94(2)
C(1)	34(1)	40(1)	54(1)	-1(1)	-7(1)	-14(1)
C(2)	33(1)	49(1)	59(2)	-3(1)	-12(1)	-14(1)
C(3)	26(1)	42(1)	74(2)	-5(1)	-9(1)	-12(1)
C(4)	65(2)	45(2)	62(2)	-6(1)	0(1)	-23(1)
C(5)	46(1)	49(2)	59(2)	-1(1)	-14(1)	-20(1)
C(6)	27(1)	37(1)	57(2)	-2(1)	-5(1)	-12(1)
C(7)	46(1)	49(2)	54(2)	3(1)	-6(1)	-16(1)
C(8)	54(2)	68(2)	66(2)	6(1)	1(1)	-26(1)
C(9)	88(2)	102(3)	82(2)	11(2)	13(2)	-49(2)
C(10)	143(3)	134(4)	68(2)	-8(2)	34(2)	-72(3)
C(11)	156(4)	128(3)	60(2)	-13(2)	11(2)	-80(3)
C(12)	93(2)	86(2)	59(2)	-3(2)	2(2)	-50(2)
C(13)	111(2)	60(2)	59(2)	-10(1)	6(2)	-36(2)
C(14)	148(4)	120(3)	72(3)	-26(2)	33(3)	-45(3)
C(15)	255(7)	127(4)	71(3)	-25(3)	41(4)	-73(5)
C(16)	295(8)	154(5)	50(3)	-8(2)	-10(3)	-127(5)

Table S5. Hydrogen coordinates (x 10^4) and isotropic displacement parameters ($Å^2$x 10^3) for mk56b.

	x	y	z	U(eq)
H(2)	4140	2458	3649	60
H(5)	9191	2407	6607	65
H(8)	9954	4055	3465	81
H(9)	11739	3942	2114	115
H(10)	10838	2439	996	143
H(11)	8168	963	1235	139
H(12)	6448	943	2599	97
H(14)	3054	2332	8447	146
H(15)	4450	2523	9956	192
H(16)	7958	2783	9706	199
H(1N1)	9390(30)	2460(40)	4707(16)	71(8)

Table S6. Hydrogen bonds for mk56b [Å and °].

D-H...A	d(D-H)	d(H...A)	d(D...A)	<(DHA)
N(1)-H(1N1)...O(1)#1	0.898(16)	1.879(19)	2.7064(19)	152(2)

Symmetry transformations used to generate equivalent atoms:

#1 x+1,y,z

3. Microwave profiles for compound 3m

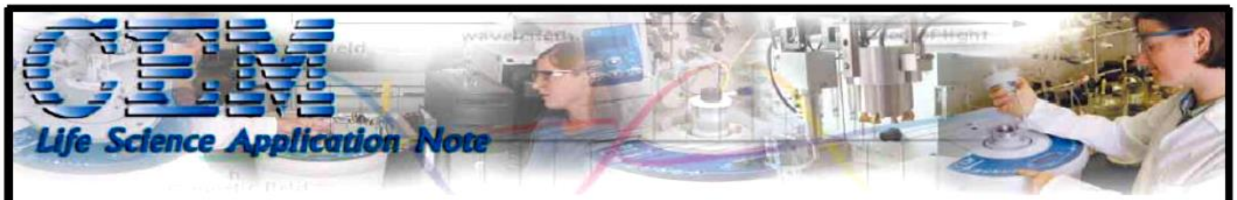

Reaction

MK56 - 5 mins Pyrazole 150 User: Mark Kelada 10mL Vessel Snap Cap

Method Parameters

Name: Pyrazole 150

Prestirring(mm:ss):	00:00

Type: Dynamic

Stage	Temp(C)	Time(mm:ss)	Pressure(BAR)	Power(W)	PowerMAX	Stirring
1	150	05:00	17.0	100	No	High

Graphs

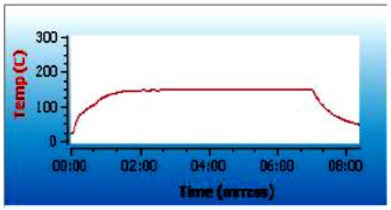

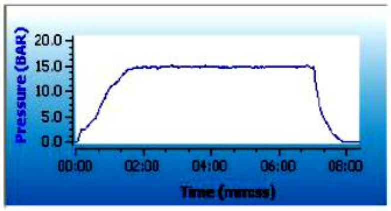

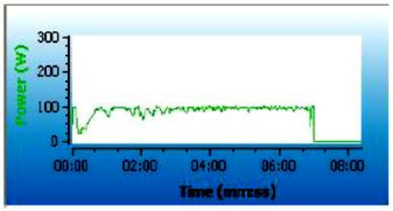

Method Summary

Reaction started:	20/04/2017 14:36:11
Temperature setpoint reached:	20/04/2017 14:38:10
Reaction cooling started:	20/04/2017 14:43:11
Cooling/Reaction ended:	20/04/2017 14:44:33

Reaction Completed Successfully!

Maximum temperature:	150 C
Maximum pressure:	15 BAR
Time to obtain setpoint:	01:59 mm:ss
Time at setpoint:	05:01 mm:ss
Time cooling:	01:22 mm:ss

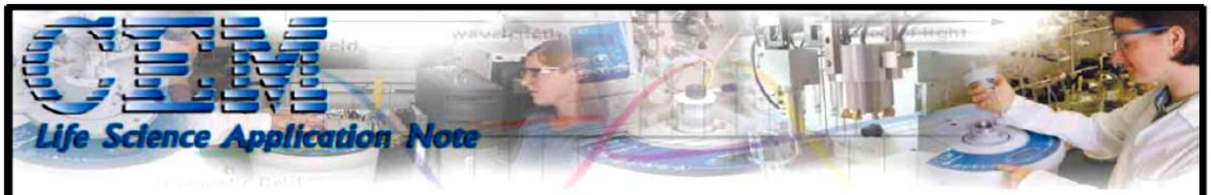

Reaction

MK56- 2hrs RTB69 150 2h   User: Mark Kelada   10mL Vessel   Snap Cap

Method Parameters

Name: RTB69 150 2h

Type: Dynamic

Prestirring(mm:ss):	00:00

Stage	Temp(C)	Time(hh:mm:ss)	Pressure(BAR)	Power(W)	PowerMAX	Stirring
1	150	02:00:00	17.0	100	No	High

Graphs

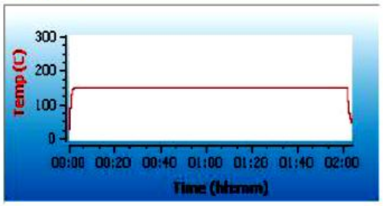

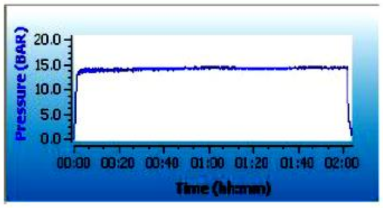

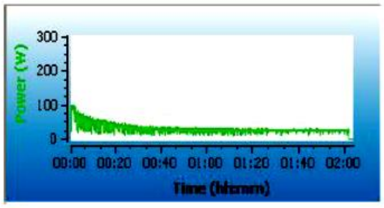

Method Summary

Reaction started:	20/04/2017 15:07:04
Temperature setpoint reached:	20/04/2017 15:09:01
Reaction cooling started:	20/04/2017 17:09:03
Cooling/Reaction ended:	20/04/2017 17:10:56

Reaction Completed Successfully!

Maximum temperature:	151 C
Maximum pressure:	15 BAR
Time to obtain setpoint:	01:57 mm:ss
Time at setpoint:	02:00:02 mm:ss
Time cooling:	01:53 mm:ss

Accelerating the transformation of concept to cure
P.O. Box 200 Matthews, NC 28106 • 800.726.3331 • www.cemsynthesis.com

4. References

1 M. C. Bagley, T. Davis, M. C. Dix, C. S. Widdowson and D. Kipling, *Org. Biomol. Chem.*, 2006, **4**, 4158.

2 S. T. Moe, A. B. Thompson, G. M. Smith, R. a. Fredenburg, R. L. Stein and A. R. Jacobson, *Bioorganic Med. Chem.*, 2009, **17**, 3072–3079.

3 F. Lassagne, K. Snégaroff, T. Roisnel, E. Nassar and F. Mongin, *Heterocycl. Commun.*, 2011, **17**, 139–145.

4 M. Krasavin and I. O. Konstantinov, *Lett. Org. Chem.*, 2008, **5**, 594–598.

5 D. M. Neil J. Kallman, Kevin P. Cole, Thomas M. Koenig, Jonas Y. Buser, Adam D. McFarland, LuAnne M. McNulty, *Synthesis (Stuttg).*, 2016, **48**, 3537–3543.

6 N. Suryakiran, T. S. Reddy, K. A. Latha, P. Prabhakar, K. Yadagiri and Y. Venkateswarlu, *J. Mol. Catal. A Chem.*, 2006, **258**, 371–375.

7 S. Gogoi, K. Shekarrao, P. P. Kaishap, S. Gogoi and R. C. Boruah, *Tetrahedron Lett.*, 2014, **55**, 5251–5255.

8 I. Okazaki, Toshio; Suga, Akira; Watanabe, Toshihiro; Kikuchi, Kazumi; Kurihara, Hiroyuki; Shibasaki, Masayuki; Fujimori, Akira; Inagaki, Osamu; Yanagisawa, *Chem. Pharm. Bull. (Tokyo).*, 1998, **46**, 69–78.

9 K. Senga, T. Novinson, H. R. Wilson and R. K. Robins, *J. Med. Chem.*, 1981, **24**, 610–613.

10 N. L. Nam, I. I. Grandberg and V. I. Sorokin, *Chem. Heterocycl. Compd.*, 2003, **39**, 1210–1212.

11 S. J. Tantry, V. Shinde, G. Balakrishnan et al., , *Med. Chem. Commun.*, 2016, **7**, 1022–1032.

12 G.M. Sheldrick,.Acta Crystallogr. A 2015, 71, 3–8.

13 G.M. Sheldrick, Acta Crystallogr. C 2015, 71, 3–8.

14 McArdle, P. J. Appl. Crystallogr. 2017, 50, 320–326.
